# Pouring Rights Contracts Between Soda Companies and Public Universities: An Institutional Barrier to Sugar-Sweetened Beverage Reduction

**DOI:** 10.34172/ijhpm.8879

**Published:** 2025-11-05

**Authors:** Luc L. Hagenaars, Jennifer Falbe, Gwyneth M. Manser, Laura A. Schmidt

**Affiliations:** ^1^Department of Public and Occupational Health, Amsterdam UMC Location University of Amsterdam, Amsterdam, The Netherlands.; ^2^Philip R. Lee Institute for Health Policy Studies, School of Medicine, University of California San Francisco, San Francisco, CA, USA.; ^3^Department of Human Ecology, University of California Davis, Davis, CA, USA.; ^4^Geography Graduate Group, University of California Davis, Davis, CA, USA.

**Keywords:** Privatization, Commercial Determinants Health, Public-Private Partnerships, Procurement, Students, Institutionalization

## Abstract

**Background:**

Sugar-sweetened beverages (SSBs) contribute to obesity, cardiometabolic diseases, and plastic pollution. International health agencies have called upon public-sector organizations to use responsible procurement policies to reduce SSB consumption. Many US public universities cannot do so because of "pouring rights contracts" (PRCs) with Coca-Cola or PepsiCo that grant companies monopoly rights to sell their beverages on campus.

**Methods:**

We investigated why universities participate in PRCs using participant observation of a consortium conducting beverage research on all 10 University of California (UC) campuses. We also conducted two rounds of interviews with public university staff in California whose work involved PRCs (n = 26 and n = 25).

**Results:**

PRCs were polarizing. University managers in health and sustainability generally opposed them, dining managers held mixed opinions, and managers in athletics, procurement, contracts, and business partnerships were generally supportive, valuing the discretionary funding streams PRCs provide. These supportive managers tended to form small, but influential coalitions who internally supported the continuation of PRCs, benefiting from reduced transaction costs for their departments because PRCs streamlined contracting and procurement. These supportive managers often had a market orientation that valued economic freedom, contradicting opinions held by opponents of PRCs who stressed that PRCs create a monopoly. Supportive managers also assumed that consumers, not soda companies, are responsible for SSB-related harms, despite acknowledging that PRCs "hook" students on soda.

**Conclusion:**

PRCs are an institutionalized barrier to responsible beverage procurement by concentrating interests favorable to PRCs within the university, even when PRCs contradict the broader, but diffuse, interests of the wider campus community. This suggests that health-harming industries only need to target small, strategically positioned groups of stakeholders within public-sector organizations to achieve corporate capture of public procurement. More responsible procurement policies require public-sector organizations to bolster the financial transparency of contracting managers, request for proposals (RFP) processes, and procurement contracts.

## Background

**Key Messages**

**Implications for policy makers**

- Responsible public procurement is an important, but underutilized, policy lever for promoting human and planetary health, including through sugar-sweetened beverage (SSB) reduction.
- “Pouring rights contracts” (PRCs) that grant Coca-Cola or PepsiCo monopoly rights on US university campuses are institutional barriers to doing this.
- Our study found that PRCs had become institutionalized on financial, organizational and cultural levels of US universities, despite contributing less than 0.1% of mean total university revenue and being a subject of debate.
- We found that PRCs create small funding streams controlled by specific university directors or managers, who became advocates within the administration supportive of PRC continuation, even when this competed with the broader health and environmental mission of the university.

**Implications for the public**

Public procurement policies–rules governing how public-sector organizations buy goods and services–have great potential for promoting health and sustainability. In US universities, “pouring rights contracts” (PRCs) have historically granted Coca-Cola or PepsiCo exclusive rights to sell and market their products on campus, potentially limiting healthier options and the university’s ability to reduce single-use plastic. Our research shows these contracts have become embedded in the financial, organizational, and cultural fabric of US public universities in California, explaining why PRCs persist despite generating less than 0.1% of mean total university revenue and being a subject of debate. The case of PRCs suggests that companies selling goods for procurement may only need to engage a few key stakeholders to entrench their interests.

Sugar-sweetened beverages (SSBs) are major contributors to obesity and related diseases.^1,2^ SSBs have been associated with 184 000 deaths per year worldwide^2^ and over 50 000 deaths per year in the US.^3^ In addition, Coca-Cola and PepsiCo, the two largest SSB companies,^4,5^ are the top two corporate contributors to plastic pollution,^6^ with each company generating approximately $40 billion annually in global beverage sales.^7-9^

 Prior research on the commercial determinants of health^10,11^ has investigated how soda companies obstruct and delay the international adoption of evidence-based public health policies that would reduce SSB consumption.^12,13^ There is a large literature showing how Coca-Cola and PepsiCo, often working through trade organizations, fund political donations, “grassroots” activism, trade litigation, and corporate social responsibility campaigns to prevent the passage of soda taxes^12-16^ and front-of-package warning labels^17,18^ in countries around the world.

 In light of these harms and barriers, international health bodies have begun to recommend responsible public procurement policies to reduce SSB consumption.^19-22^ Under responsible procurement policies, taxpayer-funded entities—such as universities, public schools and health systems—establish rules and guidelines for the “responsible sourcing” of foods and beverages that promote health and environmental sustainability.^19^ The World Health Organization (WHO),^20^ International Chamber of Commerce,^21^ the Organisation for Economic Co-operation and Development,^19^ and Intergovernmental Panel on Climate Change^22^ have all identified public procurement as an underutilized policy lever for promoting human and planetary health. Clinical trials^23,24^ and simulation models^25^ show that, when large organizations adopt healthy beverage initiatives and stop procuring SSBs, employees experience improvements in metabolic health and related healthcare spending.

 This study focuses on public university “pouring rights contracts” (PRCs) as a potential impediment to the widespread adoption of responsible procurement policies in higher education in the US. PRCs are procurement contracts in which the public university grants either Coca-Cola or PepsiCo an exclusive right to serve, sell, and market beverages on its campus, and the soda company becomes an official sponsor of the university and/or its student athletic teams.^26-30^ So far, studies of PRCs have been confined to the US, where a 2019 national survey found that at least 87% of large public universities have a PRC with primarily Coca-Cola or PepsiCo.^26^ “Beverages” under PRCs typically include all carbonated and non-carbonated soft drinks, packaged water and fruit juice, bottled coffee and tea drinks, and energy and sports drinks. Tap water, water supplied in multiple gallons, milk, brewed coffee and tea, and freshly squeezed juice are often excluded (eg, Supplementary file 1, p. 1).

 Content analysis reveals the advantages of PRCs for Coca-Cola and PepsiCo are clear.^26^ These agreements grant soda companies monopoly rights to the campus beverage market, including large numbers of campus-dwelling students. By creating an on-campus monopoly, PRCs concentrate student exposure to a single company’s brands through on-campus vending machine logos and other marketing opportunities, allowing companies to build brand loyalty.^26,29,31^ PRCs also help Coca-Cola and PepsiCo attract significant marketing exposure to stadium crowds through college athletics. For instance, the 2021 US college football national championship was viewed by 22 million fans,^32^ while 92 003 fans attended a college volleyball game in Nebraska in 2023.^33^

 While the advantages of PRCs for soda companies are apparent, for universities, they impose limitations on what can be sold in university vending, cafeteria and student dining venues. On average, PRCs last for 8-10 years and confine nearly all non-alcoholic packaged beverage sales to either Coca-Cola or Pepsi products (some contracts allow campuses to sell small amounts of other beverages, so long as this amounts to less than about 10% of sales or options).^26^ Most PRCs include “volume incentive” provisions that require campus stores and dining halls to implement marketing campaigns for flagship sugar-sweetened cola brands.^26,29,34^ When asked, university managers often point to PRC “sponsorship fees” as the main advantage. Sponsorship fees can support a wide range of campus activities, ranging from student athletic scholarships to sustainability projects. However, studies show that these sponsorship fees generate, on average $930 153 per year (range $128, 000-$2 944 385),^26^ constituting only 0.06% of mean total revenues for the average large public university.^28,35^

 The literature suggests that PRCs can have significant downsides for universities. Universities can risk reputational harm^10,19^ because requirements of the PRC conflict with campuswide health and environmental sustainability policies (eg, tap water promotion, single-use plastic bans).^30,36,38,39^ By creating campuswide monopolies, PRCs may lead to higher beverage prices, thereby exacerbating campus efforts to mitigate student food insecurity.^38^ PRCs also raise concerns about student privacy because they allow Coca-Cola or PepsiCo to target students with marketing^37^ and use images of student athletes in soda company marketing.^39^ Finally, surveys suggest that, when made aware of PRCs, majorities of university students, faculty and staff members oppose them due to concerns about higher prices, health, and sustainability.^28,38-42^ In a study of large California public universities, 81% of students opposed PRCs, even while overestimating their university’s PRC sponsorship revenue by 100-1000 fold.^28^ In another study, 62% of students, faculty and staff were unsupportive of PRCs.^30^

 In a small number of universities, tensions about continuing or initiating a PRC have led to organized student opposition, including attempted boycotts, disrupting university events, demonstrations, and other collective action directed towards university Chancellors.^39-41^ To our knowledge, however, student opposition has usually failed, with three exceptions. Students on campuses of the Universities of California (Humboldt)^42^ and Vermont^43^ ended existing PRCs, and students prevented a new PRC on San Francisco State University.^44^ Meanwhile, organized efforts failed to dislodge PRCs on other campuses, including Berkeley, Davis, and Santa Barbara.^5,36,38-41,45^

 In this study, we investigated why most public universities in California continue to participate in PRCs despite their apparent misalignment with universities’ broader health and environmental missions,^46,47^ and in the face of student opposition. We conducted two iterative rounds of key informant interviews with university directors and managers (hereafter referred to as “managers”) who directly work with PRCs. Wave 1 interviews established that most managers in student health and campus sustainability opposed PRCs. Managers with roles in athletics as well as procurement, contracts and business partnerships (hereafter “contracts”) were often favorable to and actively advocated for PRCs. In Wave 2 we interviewed managers who mostly supported PRCs to better understand perceptions of their interests in them. Simultaneously, we conducted participant observation in a case study of the PRC renewal process at University of California (UC) Berkeley.

## Methods

###  Design

 This study draws on a grounded theory methodology^48,49^ and the institutional theory of organizations^50-52^ to understand the dynamics amongst stakeholders within public universities across California concerning PRCs. Grounded theory guided our sampling and data collection, with 26 interviews with university managers in Wave 1 revealing that PRCs are polarizing, prompting interviews with 25 other managers in Wave 2 to explore these tensions more closely. Participant observation further illuminated why student efforts to dislodge PRCs on one large campus, UC Berkeley, failed. During analysis, the institutional theory of organizations provided a conceptual framework for interpreting how PRCs might become institutionalized in public universities, one focused on how material interests and taken-for-granted assumptions combine to make systems resistant to change, or subject to “organizational inertia.”^50-52^ Appealing to the constructivist paradigm, we acknowledge that data collection and analysis were shaped by interactions between the researchers and study participants.

###  Study Setting

 For this study, we focused on undergraduate campuses of the California State University (CSU) and UC systems, which are two of the largest public university systems in the world. California has frequently been an early adopter of public health and sustainability policies. It was home to a sweep of local SSB taxes in the mid 2010s,^53^ was the first US state to ban single-use plastic bags,^54^ and prides itself as a world leader in climate action.^54^

 With 63 000 employees and a total budget of $8.2 billion, the 23-campus CSU system enrolls more than 450 000 students, making it the nation’s largest public university system.^55,56^ The UC system consists of 10 campuses that house almost 300 000 students and almost 250 000 faculty and staff, making it California’s third largest employer, with an annual operating budget of $51.4 billion.^57^ Both the UC and CSU systems have formal policies promoting health, sustainability and support for small businesses.^46,56-63^ The UC president had pledged to take a leadership role on climate change and health promotion,^46,47^ including a commitment to phase out the purchase, sale and distribution of single-use plastic beverage bottles by January 1, 2023.^62^ The CSU’s mission “is focused on cost-effective, equitable climate protection.”^59^ All 9 UCs with undergraduate campuses have a PRC with either PepsiCo or Coca-Cola. To our knowledge, across the 23 undergraduate CSU campuses, only San Francisco State^44^ prevented, and Humboldt State^42^ dislodged a PRC due to student opposition. A study of sponsorship money from PRCs in 3 Northern Californian public universities found that it amounted to 0.01% to 0.04% of their total annual revenue.^28^

 Participant observation for this study occurred in 2022-2023 and included following a failed effort by UC Berkeley students to oppose their campus’s renewal of a 10-year PRC with PepsiCo. UC Berkeley is one of the world’s largest undergraduate campuses, with a ~$3 billion annual budget, almost 46 000 students, an National Collegiate Athletic Association Division 1 athletics program, and a historic association with the free speech movement.^47^ Box 1 describes details of UC Berkeley’s new PRC with PepsiCo,^63^ renewed on September 28, 2023, and obtained through a freedom of information request. The contract contains several earmarked spending allocations (eg, alumni associations, athletics, new programs); commissions to the University over the percentage of vending machine sales; donations of carbonated soft drinks and bottled water with a 2:1 ratio for emergency supplies; a “carve out” that allows the university to use 15% of shelf space to non-Pepsi products; and a selection of detailed branding opportunities, including co-branding with wellness activities.

**Box 1. Selected Elements From Renewed Pouring Rights Contract Between UC Berkeley and PepsiCo, 2023-2033^a^**

- Annual sponsorship fees range from $570 000 in year 1 to $722 059 in year 10 [approximately 0.02% of UC Berkeley total budget in 2023 USD].
- For each percent of decline below 59 863 units sold each year, sponsorship fees will be reduced by the percent of decline. For each percent above this threshold, the university will receive a rebate on the percent increase multiplied by the fees for that year.
- A [non-specified] portion of sponsorship fees will be paid directly to the Cal Alumni Association.
- $30 000 annually to be used by university in furtherance of university’s sustainability endeavors.
- $120 000 annually in support of marketing activities related to this agreement.
- $15 000 annually to be used for mutually agreed upon programming to enhance existing programs or new opportunities each year.
- PepsiCo agrees to provide University with commissions as a percentage of the actual cash collected by PepsiCo from the Vending Machines placed at the Facilities.
- PepsiCo agrees to provide annually up to 2400 cases annually of 12oz carbonated soft drink cans and 1296 cases of 16.9oz Aquafina for university’s emergency water supply.
- University is allowed to allocate 15% of retail shelf space to non-Pepsi products.
- Sponsor designation/placement on all “Wellness Happens Here” printed and digital PepsiCo marketing materials, opportunities to table at the Recreational Center, sponsor select group exercise classes and create customized programming for event.
- Up to 6 PepsiCo logo inclusions on murals throughout the Recreational Sports Facility.
- The rights for PepsiCo to use Cal Athletics’ trademarks and logos.
- 250 000 ad impressions on Calbears.com [official website of UC Berkeley Athletics].
- Two social media posts during football and 2 during basketball season.
- Opportunity for 2 Pepsi-Co designated individuals to travel with football team to away game of PepsiCo’s choice.
- Four Facebook posts highlighting Pepsi’s partnership with the university.
- Exclusive non-alcoholic beverage sponsorship for Homecoming for the entire length of contract.

-----------------

Abbreviation: UC, University of California.

^a^See Supplementary file 1 for the complete contract.

###  Sample and Procedures

####  Participant Observation

 Three coauthors actively followed and participated in PRC-related activities of the UC Research Consortium on Beverages & Health (henceforth “the Consortium”), an interdisciplinary group of faculty members and graduate students conducting research on beverages across all 10 UC campuses.^64^ Authors JF and LAS had been part of the Consortium since its inception in 2018, and studied beverage environments and procurement on their own campuses. LLH, a visiting scholar from Europe, brought an external perspective to the Consortium between August 2022-August 2023, participating in 8 Consortium meetings Consortium and numerous smaller workgroup meetings.

###  Key Informant Interviews

 Study participants were identified through staff directories at UC and CSU undergraduate campuses in California. During Wave 1, universities were purposively sampled to provide variety in geographic distribution, PRC sponsor (PepsiCo vs. Coke), and PRC status (PRC, dislodged PRC, prevented PRC). During Wave 2, staff at a census of UC and CSU undergraduate campuses were invited to participate. Interviews were held by videoconference except for three (Wave 1) and two (Wave 2) in-person interviews. We took notes and transcribed recorded interviews verbatim.

####  Wave 1

 During 2019-2022, JF and GMM conducted a first wave of interviews with 26 university managers who work in departments of student health (n = 7), dining (n = 9), athletics (n = 4), and contracts (n = 6) at 4 CSU and 3 UC campuses.^65^ These interviews focused on details of PRCs such as their duration, sponsorship fees, and how the exclusivity that they establish works, how they are negotiated, and the perceptions of managers towards PRCs (See Supplementary file 2 for interview schedule). We found that PRCs are a polarizing topic and observed consistent patterns in the types of managers who favor and oppose them. Most university managers working on contracts and “business partnerships,” along with athletics departments, were favorable to PRCs, while those in student health offices often opposed PRCs. Campus dining managers appeared to be more divided.^65^ Following a grounded theory approach, we conducted a second wave of interviews focused on university managers in athletics, contract, and dining departments, to better understand their perceptions and the nature of their advocacy for PRCs.

####  Wave 2 

 Interviews with contracts, athletics, and dining managers, who often came from business backgrounds, were held in 2023 by LLH, and focused on the reasons for favoring PRCs and how managers went about managing and advocating for them (See Supplementary file 3 for interview schedule). The invitations for these interviews were either accepted directly or forwarded internally to the individual responsible for PRC oversight. In one instance, the invitation reached a soda company sales representative. One participant declined an interview but provided written responses. Detailed notes were taken for another participant who declined recording. The final sample of 25 managers included staff working in dining and auxiliary student services (n = 7); athletics (n = 2); contracts (n = 15); and soda corporations (n = 1). These managers covered 7 UC and 15 CSU campuses, of which 2 campuses dislodged or prevented a PRC.

###  Data Analysis

 Data collection and analysis were conducted iteratively, and focused on identifying factors that explain why many public universities in California participate in PRCs, and why they PRCs are challenging to change. In weekly meetings led by LLH, the three authors first discussed emerging themes and trends from Wave 1 interviews and participant observations. As inquiry progressed, the project team became more focused on questions about the dynamics between stakeholders favorable and opposed to PRCs, and how PRCs persisted despite polarization and, in some cases, active opposition. At this stage, data from Wave 2 interviews and observations of the PRC renewal process at UC Berkeley stood central. Informed by the institutional theory of organizations,^50-52^ coding and analysis focused on themes related to the financial costs and benefits of PRCs to the university, the organizational arrangements through which PRCs are negotiated and implemented, and the cultural frames used by university managers to interpret these agreements. Coding and analysis used MaxQDA Standard 22.6.1 and specific codes included (1) financial costs and benefits of PRCs, (2) the organizational arrangements through which PRCs are negotiated and implemented on the campus, (3) arguments for and against PRCs, including words and phrases used to defend or criticize them, and (4) relevant contextual factors. We constantly compared new data, codes, and themes, and participant observations, until saturation was reached with no new insights being generated.

## Results

###  Polarized Views on Pouring Rights Contracts

 A key finding from Wave 1 interviews was that PRCs were usually polarizing. Athletics managers, who were mostly favorable to PRCs, often emphasized that payments from soda companies supported athletics financially: “I believe it’s a 50/50 split… We [the athletics department] get $100,000 and they [the campus] get $95,000.” Athletics managers often emphasized the benefits of PRC funds for athletics and to some extent for students, for instance, “to push student attendance [to games] with t-shirt giveaways” and that per year “$35,000 went straight to scholarships… and $20,000 went to scoreboard maintenance.” Contracts managers were also uniformly supportive of PRCs, arguing they “create new revenue for the new university president,” and that without PRCs, in some cases, “there would be no more athletics, it would lay off people.” Contracts managers liked PRCs for streamlining contracting: “Once the contract got implemented it was really a couple hours a month… maybe a day a year of trying to coordinate the campus collaborators.”

 Dining representatives appeared generally divided in their support for PRCs. Some mentioned PRC “funding [was used to] to support different student groups” and how “they [PepsiCo] do a lot of giveaways… product offerings that our students want to buy” and that “they’re [PepsiCo] trying to be good partners here on campus.” However, some noted that “dining services does all the work and other departments get the benefit.” This work encompassed enforcing exclusivity clauses as well as experiencing “pressure to hit [sales] targets,” referring to the volume incentives part of PRCs.^26^ Others noted that PRCs increased wholesale prices and therefore prices students pay for beverages and limited choice, especially for healthy beverages.

 Student health and sustainability managers were almost universally opposed to PRCs. Sustainability concerns involve requirements to have “lots of bottled water everywhere.” Health managers suggested that PRCs were “promoting unhealthy lifestyles,” and that “state institutions shouldn’t be having partnerships with corporate entities.”

 On the UC Berkeley campus, research centers focused on health and sustainability, staff, and faculty partnered with student groups to oppose PRCs, and students formed a “Pour Out Pepsi” campaign that opposed the renewal of UC Berkeley’s PRC, which stated in its campaign pamphlet^66^ that:

 “*The presence of PepsiCo in the Berkeley community ushers in dire concerns on many fronts, including but not limited to climate change, water use, and plastic waste pollution. Additionally, students on the Berkeley campus are confronted with problems of health inequality and food insecurity brought on by the lack of options.” *

 Wave 1 interviews suggested that the benefits and downsides of PRCs did not accrue evenly to all university departments, and that athletics and contracts managers potentially had more power in negotiating a PRC, compared with dining managers, health promotion staff, and students. An athletics managers noted that students’ views could be bypassed by carefully timing contract renewals:

 “*If you want to just be done [negotiating a PRC], do it in summer when no one’s around. I think that’s the problem with the one we had, they were dragging it out to get the number that they wanted and by the time school started and students got wind of it, our RFP [ request For proposals*]* failed. They should have just signed it, and took the money and run.”*

 Dining managers suggested that their unit’s dependency on the PRC funds was a rationale for continuing the contract:

 “*Honestly, I’m a pawn in the game, and you know I try to push back where I can*… *But I have to make it. I have to re-evaluate and renegotiate our contract this year*… *I have too much to lose economically.”*

###  Financial Costs and Benefits of Pouring Rights Contracts

 In Wave 2 interviews, university managers representing contracts, athletics, and dining acknowledged that there were competing interests in PRCs. But they emphasized that attracting Coca-Cola and PepsiCo sponsorship payments to the university was imperative. As one dining manager articulated: “campus just wants the most money.”

 Most managers saw maximizing sponsorship revenue, not health, sustainability, or pricing considerations, as their main objective: “My job gets real simple. Until told otherwise, we’re gonna try to bring in as much money as possible.” University leadership often did not give these managers directives to take non-financial aspects into account: “Put the flag in the ground, and we’ll all follow. But that has to come from [UC system president]. If you leave it up to the individuals, they’re gonna go with what their campus can tolerate.” Many managers emphasized that declining state funding for public universities created this fiscal need: “Our own leadership for the state is telling us to find alternative sources of revenue.”

 Managers overseeing athletics and contracts emphasized the need to promote sales of the sponsor’s beverages to meet PRC volume incentives (ie, minimum amounts of products that universities must sell to receive sponsorship payments), including by putting pressure on dining to sell more of the sponsor’s products:

 “*What happens when you have these PRCs? Pretty soon the dining service goes, ‘I don’t like Pepsi anymore,’ and they try to find ways not to use [Pepsi]… We needed to tell them: ‘You’re an integral part of our volume. So, you need to promote this, and you need to be able to meet those volumes.’”*

 Throughout our Wave 2 interviews, managers emphasized that PRCs generated a small but precious funding stream because these revenues were not subject to spending restrictions: “Pouring rights funding is unrestricted… A dollar unrestricted is worth probably a dollar fifty, because they can decide however they want to spend it.”

 In one case, an informant reported that the chancellor’s office had threatened to retain all PRC revenue for its purposes. Bargaining over which units received earmarked soda company dollars could be heated, with the (re)allocation of those dollars used for appeasing criticism:

 “*The entity that manages the contract could always switch some categories around... So, if they’re being accused of equity washing, having a minimal amount of money going into scholarships, that organization could*… *switch around another category to pump that number up a little bit…. It’s that shell game, right? We just move the little pebble inside the cup.”*

 While at most universities, athletics was earmarked to receive the most sponsorship funds, portions could support occasional capital projects (eg, a concessions pavilion) and other university department:

 “*There’s buckets of money for sustainability, student engagement. There’s a variety of buckets of money in addition to the main bucket that goes to the campus…. All those buckets’ distributions go through me, so that we know that they’re all accounted for every year.”*

 At some universities, PRC sponsorship payments were used to fund the salaries of the contract managers who negotiated them: “Pouring rights revenue is earmarked to help support and fund the [business partnerships coordinator] that’s helping to enforce it*…. *So that position.”

 The perceived financial benefits of PRCs were seen differently by some managers on campuses without PRCs:

 “*[With a PRC], we would have taken away the market. We would have been saying this is a closed market community, and nothing ever flourishes in a closed market community. The person that is close to the market is going to exploit that and make every decision based on what’s best financially. And not on what’s best for the students or the university.”*

###  Organizational Management of Pouring Rights Contracts

 Many of the university managers interviewed for this study described careers as managers in the food and beverage industry prior to joining the university; some in the CSUs had previously run an independent food service operation on their campus. Several dining managers were critical that PRCCs gave Coca-Cola or PepsiCo the ability “to raise prices to us each year,” which they attributed to fact that PRCs prevented them from negotiating with other beverage suppliers. Contracts manager often took a “bottom line” orientation that focused on profit maximization: “I come from more of a business perspective, so if we’re going to be selling products, we should be maximizing what we can be getting in return.” These managers also viewed compliance with public procurement law as their focus:

 “*Are we following the law when we’re entering these contracts? That’s what I’m concerned about. As far as bringing something that’s not healthy onto the campus… I’m not really in the position to [question that].”*

 PRCs typically spanned one decade, and most university managers had never worked in a situation without one. As a result, many managers in this study described well established, close working relationships with Coca-Cola or PepsiCo representatives:

 “*[Pepsi] is more ingrained. They’re going to be there every single week or day. Providing us products… There’s a constant touch point… It’s just a much more intimate relationship in that sense.” *

 Other informants described mutual satisfaction:

 “*The longer you’re working with those types of contracts, it becomes less about managing the money and more about managing the partnership, and how you can make it the best for both.” *

 Trusting relationships with soda company representatives were frequently described as beneficial to the university, in part, because soda companies provided occasional “gifts” such as trash and recycling bins featuring the soda company logo. Coca-Cola and PepsiCo representatives also assisted with installing, stocking and maintaining vending machines, donating beverages to graduation ceremonies or student orientation programs, and helping during major sports events.

 An important factor in university managers’ support of PRCs was their perceived administrative efficiency. Contracts managers preferred PRCs that span around 10 years because setting up a PRC “with the large corporations… always takes much longer than what we hope,” after which “the contract… can kind of manage itself,” while experiencing fewer supply chain issues than with multiple smaller vendors.

 The organizational benefits of PRCs were seen differently by managers with mixed or negative opinions about PRCs, because controlling exclusivity and carve outs was seen as labor intensive:

 “*[The exclusivity] is the downside, because you lose flexibility on what you can provide. One of the issues was that even though [Pepsi] had the most shelf space, one fridge which was non-Pepsi products, was always sold out, because that’s what people wanted to buy more than Pepsi products*… *They had a hard time stocking the non-Pepsi products.”*

 Our participant observation occurred during the contract renewal process at UC Berkeley. The process began with the university’s business partnerships office convening a beverage working group in April 2019 to advise on the next contract(s), following a student hackathon aimed at promoting human and planetary health in beverage procurement. The hackathon, together with PRC-related research on campus, sparked student advocacy against PRCs, including student government legislation calling for the termination of the campus’ existing PepsiCo PRC.

 The beverage working group began by exploring the merits and drawbacks of various beverage procurement models and voted against a campus-wide PRC in February 2022, while recommending the option of confining the new PRC to cover beverage vending for the athletics departments alone. The process became contentious when an advisory committee for the business partnerships office disagreed with the beverage working group, recommending a campus-wide PRC. This led to student demonstrations at the Chancellor’s Sustainability Summit in April 2022, and direct appeals to the Chancellor in a separate meeting. Ultimately, the UC Berkeley Chancellor approved a campuswide PRC renewal in July 2022. The UC Berkeley partnerships office argued that a single contract achieved administrative efficiencies, sponsorship payments, and “[greater] negotiating power with providers to better address campus sustainability and health priorities.”^67^

 Under the Chancellor’s order, the beverage working group began an RFP and process of evaluating bidders. Working group members were required to sign a non-disclosure agreement, a point of contention for some members. Meanwhile, the UC Berkeley student government passed a second resolution calling for the termination of PRCs, students began protests, and some faculty, along with local politicians and community organizations, launched a letter-writing campaign urging the Chancellor to reconsider the PRC. Ultimately, UCB renewed its 10-year contract with PepsiCo in August 2023 (Box 1). In a press release, the campus stated that “PepsiCo was selected… following a seven-month collaborative process that included a competitive RFP, gaining input from an engaged Beverage Working Group, and responding to the areas that the working group identified as most meaningful and beneficial to the campus.”^67^

###  Cultural Framing of Pouring Rights Contracts

 University managers in contracting and athletics saw PRCs as prestigious: “We must be important because Coke wants our business,” and “We like to show partnerships with national type companies. It shows strength.” Managers from smaller university campuses, often with less prestigious athletics departments, saw reputational value for their teams to co-brand with a large soda company. University managers who successfully negotiated PRCs also enjoyed personal prestige: “The [head of procurement] was instrumental in getting the original Pepsi contract. Well, he passed away… so we created a scholarship in his name.”

 Despite this perception of prestige, many informants still noted that PRCs could cause reputational damage to the university and broadly acknowledged that they were contentious, with one noting that heavy student opposition to the PRC “makes the prestige piece go away for the university.”

 Nearly all the managers we interviewed acknowledged that SSBs could have negative health effects and environmental harms. Those advocating for PRCs often qualified these two ways. First, some managers drew comparisons to other commercial products also viewed as causing harms:

 “*Okay, people get diabetes from soda and stuff like that. I get that. Alcohol has destroyed lives. I don’t remember ever reading a story about soda pop destroying anybody’s life or destroying families.” *

 Another informant mentioned: “Tobacco is obviously something that could give you tongue, mouth, throat, lung cancer. I just don’t compare that to sugar or to non-healthy beverages.” Second, many managers argued that any health or environmental burdens were a matter of personal choice:

 “*People have choices to make. Like, I have type 2 diabetes, and I know how I got it. I used to drink Coke… Now, notice that I have a Diet Coke, right? And I’m addicted to that. But you know, people have choices.”*

 While one dining manager argued that it was the responsibility of parents to teach students to avoid soda consumption, another dining manager emphasized individuals should live with the consequences:

 “*So, by the government vilifying Pepsi, Coke and McDonalds… we’re enabling those people, we’re confirming that yes, you’re right, it’s not your fault that you’re fat. It’s their fault... There’s no ownership of the self anymore.”*

 When asked why Coca-Cola and PepsiCo entered into PRCs, most informants believed that it was to promote soda consumption and brand loyalty in students—“to get [students] hooked to enjoy the taste of a Pepsi product, and maybe then continue it the rest of their life.” Some went so far as to say that PRCs are “all about brainwashing.” Informants on campuses without opposition to PRCs linked these brand loyalty effects to the lack of opposition, with one manager noting that “[PRCs] are not widely known among students because they only see Pepsi.” For most informants, however, this did not discount the benefits of PRCs to the university. One informant argued that sponsorship payments were designed to mitigate harms to students:

 “*If we have a more traditional pouring rights relationship, and we’re pushing soda out all over the campus, do we feel better about ourselves? If that incentive money is going to support social justice causes or basic needs or students in crisis, is that where people are at, that they feel like they’re making up for, you know, having these things [soda] available on campus?”*

 Other contract managers noted that Coca-Cola and PepsiCo were mitigating their adverse impacts on health and the environment, with one mentioning they had “pushed Pepsi to reduce plastic use in their bottles” and another adding that: “we want them to do that learning here.” Some suggested that soda company beverage portfolios had become healthier and more sustainable over time:

 “*There’s been definitely a shift in recognizing from both brands that younger generations are moving away from the high-sugar products… So I think that their product lineup has dramatically shown in that respect.”*

 Others felt there was a need to “balance” student opposition on health and environmental grounds with pragmatism and economic realism:

 “*[Student groups] are looking to make a point and demonstrate their beliefs and values in a project without really thinking about, is this practical? Is it going to be costly? Can it be done? That’s our job at the university and the administration to make that balance.”*

 While most managers interviewed in Wave 2 took an overall favorable view of PRCs, it was notable that some volunteered that they, personally, avoided SSBs, although as one qualified, “I wouldn’t be comfortable pointing a finger at the brand, Pepsi, in any kind of campaign.” One manager appealed to their identity as a business professional to explain:

 “*I think that’s totally separate. My personal interests are separate from business interests, and I don’t live on campus. I’m not buying the food on campus. I bring my own food if I’m eating at work, and I see this PRC more as a service to our end users.”*

 These comments reinforced the themes of personal choice, as one informant explicitly noted: “When I hit 21, I decided to give up sugary beverages on a daily basis… ’Cause I’m educated right?… So, it’s all about choice.”

 Beliefs of how exclusive PRCs infringed with personal choice were seen differently by managers with mixed or negative feelings about PRCs:

 “*I think it was also a little bit paternalistic, that we could control choice… And say well choose this rather than try to create a social experiment of our own. Give students resources to make the right choice, by having everything available.”*

 Compared to contracts and athletics managers, PRCs were seen as undesirable especially by health managers: “I don’t think a university is a place where foods or any products that could be detrimental to health should be promoted.” The impact of PRCs on single-use plastic waste was seen as “huge” by students, who on many campuses were seen as part of a more skeptical generation as it relates to “corporate greed and how the corporation is behaving.”

 At UC Berkeley, food insecurity proved to be a major factor in student opposition to PRC renewal, with students arguing that an exclusive PRC was “forcing our campus community to buy [Pepsi] products because they are the only available options” and that “if the University chose to end its contract with PepsiCo, dining halls, cafes, and restaurants would be able to provide students with an affordable, accessible, and healthy selection of food and beverages.”^68^ In interviews, dining managers echoed this criticism, noting:

 “*We had to participate in the contract… but what happens is then Pepsi turns around and charges me a much higher price than if I was able to negotiate with Coke and Pepsi for just products. Competition is all on price.”*

 Another dining manager noted that price differences were not negligible: “The price that we have to charge for a Pepsi compared to a Walgreens, our price is probably 50 cents more.”

## Discussion

 Most large US public universities have PRCs with multinational soda companies that come with relatively small sponsorship fees for the university. By granting monopoly rights to the university’s beverage market, PRCs have clear advantages for Coca-Cola and PepsiCo. But the advantages for public universities are more mixed as these agreements may be at odds with their health, environmental,^38^ and food insecurity goals^38^ and student opinion,^28,30,40,41^ sometimes leading to student protests.^36,39-44^ We aimed to identify institutional dynamics that explain why most public universities in California nevertheless maintain PRCs.

 Our findings suggest that PRCs persist, in part, because their financial and organizational benefits typically accrue to a small subset of university departments—contracts, business partnerships and athletics—where they become valued discretionary funding streams and part of normal budgeting processes. If tensions over the PRC emerge amongst stakeholders within the university, managers in these departments can become a natural political coalition invested in the contract’s continuation. Sponsorship dollars, paid to the university by Coca-Cola or PepsiCo may make a limited contribution to the overall university budget. But for the administrative units earmarked to receive these funds, they often become a valued discretionary funding source. We also observed that in some cases, sponsorship dollars were used to fund the salaries of the managers managing these contracts. And unlike the government tax dollars flowing into the university, soda company sponsorship dollars were unrestricted and thus, could be spent at managers’ discretion.

 PRCs also appeared to reduce transaction costs – the time, effort, and resources needed to process a transaction – for the same administrative departments within public universities. Contracting managers described significant administrative efficiencies resulting from the fact that a single corporation could provide for all campus beverage needs. Contracting managers claimed it was far simpler to negotiate a single contract with one large corporation than to contract with multiple small vendors, though also mentioned that negotiating a PRC was especially resource intensive. And once the PRC was signed, Coca-Cola or PepsiCo representatives often provided equipment and help during graduation ceremonies, student orientation programs, and major sports events, relieving procurement staff. With PRC contract periods lasting, on average, 8-10 years, university managers developed trusting—even “intimate”—day-to-day working relationships with soda company representatives who could troubleshoot problems and supply favors.

 Finally, beyond these financial and organizational incentives, our findings suggest that PRCs persist because they could be defended with cultural assumptions invoked by university managers who favor them. Many athletics and contract managers viewed PRCs through a free market frame that valued efficiency and individual consumer choice, along with personal responsibility.^68^ Many of these university managers had prior careers in the food and beverage industry, and their words and imagery often echoed Coca-Cola and PepsiCo corporate social responsibility campaigns portraying consumers as free to choose what they purchase and, therefore, personally responsible for the health consequences.^69-72^ Framed as marketplace innovations, PRCs brought career prestige to the university managers associated with them. Although many informants acknowledged the health and environmental impacts of Coca-Cola and PepsiCo, these concerns were often considered outside the scopes of their “bottom line” orientation.

 Taken together, these financial, organizational and cultural factors help explain why many public universities agree to participate in PRCs despite their drawbacks. Pouring rights may create an “imbalanced political arena”^73^ within the university where the concentrated interests of university managers who directly benefit outweigh the broader, more diffuse, interests of students, faculty and staff. As we observed in the PRC renewal process at UC Berkeley, even in a case where students were able to mobilize organized opposition to a PRC, the concentrated interests of managers in athletics and business partnerships prevailed in university decision-making. PRCs appear to have become so institutionalized in these cases that they persist even in hostile environments.

 It is common for institutional research to uncover the ironies and internal contradictions within institutions that may, under certain conditions, serve as the fault lines for change.^50,74^ In this study of university pouring rights, these institutional fault lines seemed rather close to the surface. In interviews, many advocates of PRCs expressed the paradoxical view that, even while granting soda companies a campuswide monopoly on beverage marketing and sales, PRCs could still promote consumer freedom of choice. Elements of the market frame seemed to allow them to navigate this inherent contradiction. Most of the university managers in this study understood that, for Coca-Cola and PepisCo, the goal of PRCs was to “hook” young adults on their products while promoting brand loyalty. According to these managers, however, it was up to students to take personal responsibility for their choices in the campus marketplace by becoming “educated” consumers able to choose whether to get “hooked.”

###  Limitations and strengths

 Our approach to identify institutional dynamics that explain why PRCs persist used participant observation and key informant interviews, which are unobtrusive methods well suited to areas of controversy such as PRCs. A resulting limitation was that our sample of in-depth interviews that explicitly aimed to identify these dynamics was confined to public universities in one US state and a relatively small sample size of key informants that all had formal roles in PRCs. Our two-year observation period of PRC negotiations in the UCs only allowed us to observe the PRC renewal process from start to finish on one campus, and during Wave 2, we also did not formally interview students, faculty, health, senior managers, or staff likely to hold negative views on PRCs about why PRCs persist, which groups have been the subject of other reports.^28,30^ Our participant observations and sample thus limit generalizability, although we did conduct these observations and in-depth interviews after 26 interviews that explored PRCs broadly with a wider range of campus stakeholders. Furthermore, the contentious nature of PRCs may have limited the openness of our participants, despite the lead researcher being an outsider studying in the US. This may also explain why managers at 3 of 9 undergraduate UC campuses, and 7 of 23 CSU campuses, did not respond to repeated invitations in Wave 2 interviews. Relatedly, we could not verify the extent to which rationales provided for maintaining PRCs were accurate. For example, the importance of PRCs for student scholarships was mentioned repeatedly, though many PRCs provide relatively little in non-athletic student scholarship funds (eg, at one UC, only $10 000 out of over $7 Million in a year [or 0.01%] of undergraduate scholarship funding was generated by its PRC).^75^

## Conclusions

 Our analysis of PRCs in California public universities has implications for research on and action in responsible public procurement policies, which have been proposed by international agencies as an important, but underutilized, policy lever for human and planetary health. Our findings suggest that institutional barriers to procurement reforms may be deeply rooted within public-sector organizations, making these systems resistant to policy change. As far as we are aware, however, hardly any empirical research exists about the institutionalization of health-harming industry power in other public-sector organizations than US universities, even though public hospitals, schools, museums, sports stadia, government facilities, and other public sector organizations all have public procurement departments. We only came across contracts reminiscent to PRCs between alcohol industry actors and student organizations in Brazil^76^ and the Netherlands.^77^ Future studies should therefore examine the institutionalization of PRCs and other contracts that health-harming industries a monopoly position in public procurement. Transparency of public procurement contract terms is a necessity for such research, and would also help pursue action on responsible public procurement policies, for instance when it comes to prohibiting volume incentives that push universities to increase soda sales as is the case in PRCs.

 Our findings also have implications for research on how the commercial interests of health-harming industries come to drive decision-making within public-sector organizations, a phenomenon known as “agency capture” that describes situations where an organization serves private interests over its public mission.^78^ Recent studies have shown, for example, that through its public-private partnerships with governments in Latin America, Coca-Cola has successfully secured privileged access to dwindling supplies of publicly-owned water.^79^ Similarly, a recent book and scholarly articles have unpacked how a Coca-Cola-funded “exercise as medicine” initiative successfully captured the obesity prevention branch of the Chinese national government.^80-82^ Our study found that PRCs tend to create small coalitions of managers and managers within public universities who function as internal advocates for soda company interests in securing monopoly rights. This suggests that, to achieve capture, health-harming corporations may only need to focus on a small, strategically positioned group of stakeholders within the public-sector organization. The implication is, that to achieve more responsible procurement, public-sector organizations should bolster the financial transparency of contracting managers, RFP processes, and procurement contracts, to assure that they remain free from financial conflicts of interest and consider not only sponsorship payments but wholesale pricing. In addition, organizations should scrutinize procurement practices for non-monetary kickbacks that could drive contracting managers towards selective contracting, and conduct regular and transparent audits with student and faculty participation to align procurement with the organization’s broader public mission.

## Acknowledgements

 We would like to thank all research participants.

## Disclosure of artificial intelligence (AI) use

 Not applicable.

## Ethical issues

 The University of California San Francisco Human Research Protection Program Institutional Review Board qualified this research as exempt on December 6, 2022 (Reference # 362458). Before each interview, LLH, JF, or GMM explained the structure of the interviews and obtained verbal consent for participation and audio recording for transcription purposes.

## Conflicts of interest

 Authors declare that they have no conflicts of interest.

## Supplementary files


Supplementary file 1. Renewed Pouring Rights Contract Between UC Berkeley and PepsiCo, 2023-2033.

Supplementary file 2. Wave 1 Interview Questions for University Managers Who Work in Departments of Student Health, Dining, Athletics, and Contracts at University of California and California State University Campuses.

Supplementary file 3. Wave 2 Interview Questions for University Managers Who Work in Departments of Dining, Athletics, and Contracts and Who Are Involved in Negotiating and/or Implementing Pouring Rights Contracts at University of California and California State University Campuses.
