# Supplementary material for: Pouring Rights Contracts Between Soda Companies and Public Universities: An Institutional Barrier to Sugar-Sweetened Beverage Reduction
Source: Int J Health Policy Manag. 2025 Nov 5;14:8879. doi: 10.34172/ijhpm.8879 (PMC12958230; doi:10.34172/ijhpm.8879)
Supplement: Supplementary file 1 — Renewed Pouring Rights Contract Between UC Berkeley and PepsiCo, 2023-2033. [file ijhpm-14-8879-s001.pdf]

BEVERAGE SALES AND SPONSORSHIP AGREEMENT

This Sponsorship Agreement ("**Agreement**") is effective as of August 1, 2023 ("**Effective Date**") and is by and between and The Regents of the University of California, a California public corporation, on behalf of its Berkeley campus ("**University**"), and PepsiCo Beverage Sales, LLC ("**Pepsi**"), a Delaware limited liability company, and its affiliates and/or respective subsidiaries collectively comprising Pepsi Beverages Company with an office located at 29000 Hesperian Blvd., Hayward, CA 94545. Each party is referred to individually as "Party" or collectively as "Parties".

WHEREAS, University operates the UC Berkeley campus (meaning all academic, business and operational units of the University over which the Chancellor of the UC Berkeley campus has authority, formally conferred through delegations of authority from the Board of Regents or the President of the University), referred to hereinafter as the "**Campus**";

WHEREAS, Pepsi manufactures and distributes cold Beverages, and desires the right to be an exclusive promotional and marketing sponsor to the Campus in the specific areas identified in this Agreement;

WHEREAS, Pepsi wishes to identify itself with the University (on the terms set forth herein) and to promote, market, and distribute its Beverages with the University (on the terms set forth herein) and further wishes to receive the other promotional and marketing benefits provided for by the University in this Agreement; and

WHEREAS, Pepsi has competed for such opportunity, and University has determined that as of the date of this Agreement, it is in the best interests of the University to contract with Pepsi to provide for the sponsorship, marketing, and other relationship opportunities described herein.

NOW, THEREFORE, the Parties agree as follows:

DEFINITIONS

As used in this Agreement, the following capitalized terms have the respective meanings assigned thereto below.

"**Beverage** or "**Beverages**" means all carbonated and non-carbonated, non-alcoholic drinks, however dispensed during the Term of the Agreement, including but not limited to (i) colas and other flavored carbonated drinks; (i) fruit juice, fruit juice containing and fruit flavored drinks; (ii) chilled bottled coffee drinks; (iv) chilled bottled tea products; (v) hypertonic, isotonic and hypotonic drinks (sports drinks and fluid replacements), except as set forth in future Team agreements, as more fully described in Exhibit A; (vi) energy drinks; (vii) packaged carbonated or still water (including spring, mineral or purified); (viii) liquid concentrate teas ("LCT"); (ix) frozen carbonated and non-carbonated beverages ("FB"), and excluding : tap water, water supplied in 3-5 gallon containers, water dispensed from University water sources, meal replacement beverages, milk (including flavored and plant-based milk), coffee or tea, hot chocolate, juice squeezed fresh, bulk juice, smoothies and milkshakes blended on premises, and any products used for academic research.

"**Cases**" means cases of Packaged Products (as defined herein) purchased by University from Pepsi during the Term, initially delivered in quantities of 24 plastic bottles, aluminum cans, glass bottles (or equalized 24 pack cases, e.g., two 12-pack cases), eight 2-liter plastic bottles, or such other size, quantity and type of containers as Pepsi may make available from time to time during the Term and per the Campus-related requirements. For the purposes of measuring the Annual Units Threshold only, 1 Case of Packaged Product equals 1 Gallon of Postmix Product, and Case will refer to both traditional and non-traditional case packs.

"**Competitive Products**" means any and all Beverages other than the Products.

"**Designations**" refers to University's granting to Pepsi the right to promote the fact that Beverages are official beverages of University and that Pepsi is a sponsor of University, as set forth in Exhibit A.

"**Equipment**" means equipment loaned by Pepsi to University to sell or dispense, store or cool Products, full-service vending machines ("Vending Machines"), retail single-serve food service equipment and fountain service equipment (as defined below), as more fully described in Exhibit A, herein.

"**Facilities**" means buildings and other facilities owned and operated by the University.

"**Gallons**" means gallons of the Postmix Products purchased by University from Pepsi during the Term.

"**Packaged Products**" means beverages that are sold or distributed by Pepsi in pre-packaged form (e.g. bottles and cans). A current list of Pepsi's Packaged Products is listed in attached Exhibit A, which may be amended by Pepsi from time to time with University's approval, which shall not be unreasonably withheld.

"**Participating Food Service Locations**" are those locations at the Facilities, identified in Exhibit K, in which Beverages are sold and that are bound by the stipulations of this Agreement.

"**Permitted Exceptions**" refers to Competitive Products that the University may sell or distribute on Campus as per exclusivity provisions of Exhibit A of this Agreement. For the avoidance of doubt, except as explicitly outlined in this Agreement or in Exhibit A, these Competitive Products may not be advertised or promoted. Nothing herein will be construed to authorize the University to serve, dispense, or otherwise make available or permit the availability of, or in any way advertise, display, represent or promote, beverage products licensed by, or produced by bottlers licensed by, The Coca-Cola Company or any affiliate thereof, except as set forth in future Team agreements, as more fully described in Exhibit A.

"**Postmix Products**" means Beverages sold and/or distributed by Pepsi and used to create and prepare fountain beverages, frozen carbonated or non-carbonated beverages. A current list of Pepsi's Postmix Products is listed in attached Exhibit A which may be amended by Pepsi from time to time.

"**Products**" means Postmix Products, and Packaged Products.

"**Shelf Space**" means the retail display shelf footage dedicated to Beverage sales at the Participating Foodservice Locations identified in Exhibit K.

"**Teams**" means any and all NCAA varsity sports teams that use locations of the Facilities as their home stadium or field.

"**Units**" means the total combined Gallons and Cases and Packaged Products during any applicable time period. For the purposes of measuring total Units only, 1 Case of Packaged Products equals 1 Gallon of Postmix Product.

"**University Marks**" means (i) the Designations (as defined in this Agreement) and (ii) University Marks including the Campus specific and Campus-wide characters, colors, emblems, designs, identifications, logos, mascots, name, service marks, symbols, trademarks, trade names, trade dress, domain names, social media or other handles, uniforms, and other indicia of origin of University and, if applicable, the Team(s) at the beginning of the Term or which will be created during the Term, if any, but only to the extent included and for the purpose(s) specified, and according to the stipulations described in this Agreement.

"**Year**" means each 12-month period during the Term commencing on the first day of the Term or an anniversary thereof.

1. **DURATION OF TERM**. The Agreement commences on the Effective Date and will continue in full force and effect through July 31, 2033, a period of ten (10) years (the "**Term**"), unless the Agreement is earlier terminated in accordance with its terms.

2. **CONSIDERATION BY PEPSI**. Pepsi shall provide the University the monetary and non-monetary consideration described in Exhibit E during the Term of this Agreement. If Pepsi is more than sixty (60) days delinquent from the date of receiving written notice of the matter in providing any of the consideration provided in Exhibit E, and fails to cure, such delinquency shall be considered a material breach of the Agreement and may, in University's sole discretion, subject Pepsi to immediate termination pursuant to Section 20.

3. **SPONSORSHIP RIGHTS**. For purposes of this Agreement, the Products offered by Pepsi are specifically set forth in Exhibit A. Pepsi shall have the exclusive right to promote itself with regard to the Beverages described in Exhibit A. Pepsi may use the "Approved Designations" set forth in Exhibit A, and such other designations approved by University in writing from time to time. Except as set forth herein, University hereby grants to Pepsi a limited, non-exclusive, royalty-free, non-transferable, revocable, license to use and display the University Marks in the United States under the terms and conditions set forth in this Agreement, including without limitation those set forth in Exhibit C. For online uses of University Marks permitted under this Agreement, Pepsi must undertake best efforts to limit the uses in a manner directed toward only United States audiences. All promotional information, signage, and other materials, including

DocuSign Envelope ID: 5DB6E2D2-8865-4C2F-9B02-F38647AC4CB4

those listed in Exhibit B and Exhibit C, as well as all uses of the University Marks listed in Exhibit D, shall be subject to University's prior review and written approval, in accordance with written permission from a delegated authority of University of an authorized representative. Pepsi expressly acknowledges that any promotional materials that have not been pre-approved by University may not be published, distributed, publicized or otherwise used by Pepsi (or anyone on Pepsi's behalf) in any way.

4. **UNIVERSITY MARKS**. Nothing in this Agreement or in Pepsi's use of the University Marks confers upon Pepsi any right, title or interest in or to the University Marks, or in any similar marks beyond the rights granted in this Agreement. University retains all right, title, and interest in and to the University Marks except as otherwise explicitly stated herein. Nothing in this Agreement grants by implication, estoppel, or otherwise any rights to University's intellectual property, except as explicitly set forth herein. Pepsi acknowledges and agrees that, except as may be provided elsewhere in this Agreement, the license to use the University Marks granted here is provided "as is" without any warranties or representations of any kind.

5. **PEPSI MARKS**. University may use Pepsi's trademarks, marks and trade names listed in the attached Exhibit G (the "**Pepsi Marks**"), subject to a limited, nontransferable, revocable, royalty-free right and license which Pepsi grants to University solely for use in connection with, and during the Term of this Agreement. Nothing in this Agreement or in University's use of the Pepsi Marks confers upon University any right, title or interest in or to the Pepsi Marks, or in any similar marks beyond the rights granted in this Agreement. Pepsi retains all right, title, and interest in and to the Pepsi Marks except as otherwise explicitly stated herein. Nothing in this Agreement grants by implication, estoppel, or otherwise any rights to Pepsi's intellectual property, except as explicitly set forth herein. University acknowledges and agrees that, except as may be provided elsewhere in this Agreement, the license to use the Pepsi Marks granted here is provided "as is" without any warranties of representations of any kind.

6. **APPROVAL OF SPECIFIC USE OF UNIVERSITY MARKS REQUIRED**. Pepsi shall submit to University's contact identified in Section 27 for review and written approval in advance of any use, samples of Pepsi's proposed uses of the University Marks, including any and all uses in advertising marketing materials. Pepsi's use of the University Marks shall be consistent with the Trademark Use Guidelines attached hereto as Exhibit C and made a part of this Agreement; University's then-current brand guidelines; or any other guidelines or standards as communicated in writing by University from time to time. University will make reasonable efforts to review samples submitted by Pepsi and to approve or suggest necessary modifications to such samples within fifteen (15) business days after receipt of the submission. If University does not respond to Pepsi's sample within fifteen (15) business days, then such sample will be deemed rejected. Pepsi shall not materially deviate from the approved uses unless otherwise agreed in writing by University. Upon University's written request, Pepsi shall certify annually that its use of the University Marks conforms to the pre-approved samples. Changes made by Pepsi to any approved uses of the University Marks or use of the University Marks without University's approval shall be considered a material breach of the Agreement. In such case, University may, among other things, require Pepsi to modify, at its sole cost and expense, any unapproved changes and/or to discontinue any use of the University Marks or samples that were not pre-approved. To denote University's ownership of the University Marks, Pepsi shall include as required and provided by University, appropriate trademark and/or copyright notices in marketing/communication materials, in media, such as websites or on products.

7. **LIMITATIONS UPON SPONSORSHIP RIGHTS**. Pepsi shall use the University Marks only as specifically provided pursuant to the terms and conditions of this Agreement. Pepsi shall not use the University Marks for any other purpose or in combination with any other trademark or service mark without the prior advance written consent of University. Pepsi shall not manufacture or cause to be manufactured any promotional items, products or merchandise using or including the University Marks, or any marks that are confusingly similar thereto ("Licensed Products"), without the prior advance written consent of University, which may be withheld in University's sole discretion. Pepsi acknowledges that the University is currently a party to a certain license agreement pursuant to which the University has granted to a licensing agent ("Licensing Agent") a license to act as the University's exclusive representative to license the use of University Marks for certain Licensed Products. In accordance with the University's systemwide policy, "Trademark Licensing Code of Conduct" (available at http:/policy.ucop.edu/doc/3000130/CodeTrademark Licensees) and as may be amended during the Term of the Agreement, Pepsi must use a vendor licensed by University when the University's name, trademarks, or co-branding image depicting either are placed on goods to be given away or sold, or execute a separate agreement with University's Licensing Agent, and Pepsi acknowledges that Licensed Products shall be royalty bearing. Lists of licensed vendors are currently available at https://calbears.com/sports/2017/8/30/licensing.aspx, and such lists may be amended or replaced from time to time. Pepsi acknowledges that University's published requirements must be complied with in regard to the conditions under which any apparel or other material bearing the University Marks must be manufactured. The failure to observe these requirements shall be considered a material breach of the Agreement and may, in University's sole discretion, subject Pepsi to immediate termination pursuant to Section 20. Pepsi acknowledges that this Agreement is founded on the significant goodwill and positive reputation that University has developed in connection with its operations, as well as the value of that goodwill and reputation of the University and the University Marks in the minds of educational

communities, the public, and other constituencies over many decades. Pepsi agrees that it is critical that such goodwill and reputation be protected and enhanced and, toward this end, Pepsi shall not misuse the University Marks, apply to register or maintain any application or registration of a confusingly similar service mark, trademark, or logo, or use the University Marks in any way or for any purpose except as specifically authorized by this Agreement and approved by University. Pepsi agrees that the goodwill associated with and derived from Pepsi's use of the University Marks shall inure to the benefit of, and belong to, University. Pepsi further acknowledges that, other than the rights specifically granted in this Agreement, it does not now have – and in the future will not assert – any right, title or interest of any kind in or to the University Marks, nor will it challenge or contest any of the rights therein.

8. **CESSATION OF USE OF UNIVERSITY MARKS UPON EXPIRATION OR TERMINATION OF AGREEMENT**. Except as otherwise agreed in writing by University, upon the expiration of the Term or other termination of this Agreement, Pepsi shall cease use of the University Marks within ten (10) business days following such expiration or termination and, during such period, shall remove said marks from display, including without limitation all outdoor advertising, all electronic, digital, and social media, and all internet web pages, and destroy promptly all materials held by Pepsi bearing the University Marks, and cease any and all use of the University Marks. Nothing herein will require Pepsi to withdraw from circulation any materials bearing the University Marks previously distributed in compliance with this Agreement.

9. **ALLEGED INFRINGEMENT**. Pepsi shall promptly notify University in writing if any third party asserts a claim against Pepsi that Pepsi's use of the University Marks infringes or otherwise violates the trademarks or other proprietary rights of such third party. Pepsi may not initiate any action with respect to the enforcement of University's rights in or to the University Marks, including, but not limited to, pursuing or settling an infringement action; however, Pepsi shall reasonably cooperate with University (at University's sole cost and expense) in University's pursuit of any legal action designed to protect its rights to the University Marks. For the avoidance of any doubt, each Party will be solely responsible for taking such actions as it deems reasonably appropriate to obtain any trademark, service mark, or copyright protection for its respective marks.

10. **PROMOTIONAL ACTIVITIES**. Subject to the provisions of California Education Code Section 92000, University may promote the sponsorship, and Pepsi's Products and promotions, only as expressly authorized by Pepsi. Each Party shall conduct itself professionally, and not state or imply that it is acting for or on behalf of the other Party. Neither Party shall make unauthorized, false, deceptive or misleading statements or representations about the other Party or its products, services and promotions, or any general or customer-specific representation concerning such other Party's willingness to enter into any agreement. University shall obtain the prior written approval of Pepsi for the production, distribution, publication or other communication of any advertising, promotional or sales materials, or other material, relating to Pepsi, its Services, or the sponsorship, including marketing materials and any depictions of the Pepsi Marks (as defined in Section 5 above).

11. **ON-CAMPUS ADVERTISING RIGHTS**. Subject to any specific exclusion set forth in this Agreement, University hereby grants to Pepsi the exclusive right to engage in marketing and promotional activities advertising the Beverages described in Exhibit A at the Participating Food Service Locations noted in Exhibit K, subject to the prior written consent of University, which consent may be withheld at the sole discretion of University, and as set forth in Exhibit B.

12. **NO COMPETITIVE ADVERTISING OR SPONSORSHIP; EXCLUSIONS.** Subject to any specific exclusions set forth in this Agreement**,** University shall not:

(a) permit permanent or temporary advertising, signage or trademark visibility at the Participating Food Service Locations for third-party products that compete commercially with those Beverages described in Exhibit A. If Pepsi becomes aware of such unpermitted advertising, Pepsi shall inform University, and University shall employ reasonable measures to abate such advertising. Pepsi understands and acknowledges that the Campus is a facility open to the public, and that the University may require a longer period of time to abate such unpermitted advertising when messages are displayed by persons who are not employed by University.

(b) advertise, promote, depict, or merchandise commercially competing Beverages to those described in Exhibit A at Participating Food Service Locations, except as specifically and expressly provided elsewhere in this Agreement. Further, during the Term, University agrees that it will not accept or receive sponsorship or advertising revenue from any commercial seller of Beverages similar to those described in Exhibit A, except as specifically and expressly provided elsewhere in this Agreement or otherwise agreed by the Parties. Notwithstanding anything herein to the contrary, Pepsi understands and acknowledges that a university is an open environment in which members of the community who do not speak on behalf of the institution in an official capacity may advocate for their own interests and points of view in accordance with their First Amendment rights. Pepsi further understands and acknowledges that University's students, including student-athletes, are not employees and are permitted by law and University policies to exercise broad free speech rights, including student-

DocuSign Envelope ID: 5DB6E2D2-8865-4C2F-9B02-F38647AC4CB4

athletes' rights to pursue name, image, and likeness opportunities in compliance with applicable University policies, conference and NCAA regulations, and state and federal law, .

Notwithstanding the prohibitions imposed upon University described above, the following University activities and/or Facilities are expressly not subject to the requirements of this Section 12 or any other term of this Agreement:

i. The activities of any campus(es) or operational unit(s) of the University of California other than the Campus.

ii. Incidental public exposure of trademark-bearing products that may compete with Pepsi's Products. For example, the University need not restrict from public view the ordinary business use of letterhead received from competing Beverage providers.

iii. On-site advertising, merchandising of products, or point-of-sale displays located in real property leased by University to tenants who are not, as of the date of this Agreement, prohibited from advertising and displaying products that compete with products subject to University's exclusive sponsorship agreements.

iv. Activities pursuant to University's obligations to Peet's Coffee, Inc. ("Peet's") as set forth in University's July 1, 2017 agreement with Peet's;

v. Activities of the Cal Alumni Association, The Faculty Club, and The Greek Theatre;

vi. Gifts to the University, including naming rights conferred to competing service providers in connection with any gift;

vii. Research activities, including research sponsorships or gifts and use of competing products or services in connection with academic research; and

viii. All University activities at locations other than the Participating Food Service Providers. For the avoidance of doubt, activities at Anchor House are expressly not subject to the requirements of this Section 12 or any other term of this Agreement.

13. **MARKETING AND PROMOTIONAL OPPORTUNITIES**. Pepsi shall facilitate the marketing and promotional opportunities described in Exhibit B, in consultation with University.

14. **DISCLOSURE OF AGREEMENT.** Unless specifically authorized by Pepsi in writing, University shall not affirmatively publicize the business terms of this Agreement, but University may comply with any obligations imposed by law, including but not limited to the provisions of the California Public Records Act, with regard to the disclosure of such business terms and this Agreement. The Parties acknowledge that this Agreement would not be exempt from disclosure should University receive a request to disclose this Agreement pursuant to the California Public Records Act.

15. **CUSTOMER PRIVACY**. Pepsi shall not provide information regarding the identity of, or personal information regarding, individual Pepsi customers to University. If such information is provided by Pepsi to University, either intentionally or through an error, University shall not utilize such information for any purpose and shall not disclose such information to any third party unless such disclosure is compelled by law or process of a court. University shall not provide information regarding the identity of, or personal information regarding, any individual, including without limitation students, staff, faculty, alumni, and parents of students. If such information is provided by University to Pepsi, either intentionally or through an error, Pepsi shall not utilize such information for any purpose and shall not disclose such information to any third party unless such disclosure is compelled by law or process of a court.

16. **REPRESENTATIONS AND WARRANTIES**. Each Party (the "**Representing Party**") hereby warrants and represents to the other Party as of the Effective Date that: (a) the Representing Party's execution, performance, or delivery of this Agreement, the representations and warranties contained herein, and any agreement or action of the Representing Party contemplated by this Agreement, will not constitute a material breach, or be inconsistent with any law, rule or regulation of any governmental entity as presently interpreted by appropriate judicial or regulatory authority to which the Representing Party is subject or presently bound, or order of any court to which the Representing Party is subject or presently bound, or the material provisions of any contract, agreement, obligation, representation, or warranty to which the Representing Party is presently bound or under which it is presently obligated; (b) this Agreement is binding

and enforceable against such Representing Party in accordance with its terms except as limited by applicable bankruptcy, insolvency, or other laws of general application relating to or affecting the enforcement of creditors' rights generally; (c) any materials the Representing Party provides in connection with this Agreement, including but not limited to any trademarks or other intellectual property, will not infringe upon the copyright, patent, trademark, trade secret, or other intellectual property rights of any third party; and (d) each of the individuals who executes this Agreement on behalf of the Representing Party represents and warrants that they have the required authority to do so, and that this Agreement is a valid and binding obligation of the Representing Party.

17. **INDEMNITY**. Pepsi shall defend, indemnify, and hold the University, its officers, employees, and agents harmless from and against any and all liability, loss, expense, including reasonable attorneys' fees, or claims for injury or damages arising out of the performance of this Agreement (collectively, "Claim") but only in proportion to and to the extent such Claims are caused by or result from the negligent or intentional acts or omissions of Pepsi, its officers, agents, or employees, or any person or persons under Pepsi's direction and control. The University agrees to provide Pepsi with prompt notice of any such claim or action and to permit Pepsi to defend any claim or action, and to cooperate fully in such defense. Pepsi will not settle or consent to the entry of any judgment in any claim without the consent of the University, and such consent will not be unreasonably withheld, conditioned, or delayed.

18. **PEPSI'S INSURANCE**. Pepsi, at its sole cost and expense, shall insure its activities in connection with this Agreement and obtain, keep in force, and maintain insurance during the Term as follows:

(a) Commercial Form General Liability Insurance (contractual liability included) with minimum limits as follows:

Each Occurrence $1,000,000
Products/Completed Operations Aggregate $2,000,000
Personal and Advertising Injury $1,000,000
General Aggregate $2,000,000

(b) Workers Compensation as required by applicable law
(c) If a representative of Pepsi will be driving on campus, Business Automobile Insurance with insurance coverage amount of $1,000,000 per occurrence.

If such insurance is written on claims made from, coverage shall survive for a period of three (3) years following termination of this Agreement. Coverage shall coincide with the effective date of this Agreement.

Pepsi shall name The Regents of the University of California as an additional insured with respect to the coverage required above. Such provision shall apply in proportion to and to the extent of the negligent acts or omissions of Pepsi, its officers, agents, or employees, or any person or persons under Pepsi's direct supervision and control.

This insurance shall be primary in respect to The Regents of the University of California, any insurance or self-insurance maintained by The Regents of the University of California shall be excess of and non-contributory with this insurance.

Upon execution of this Agreement, Pepsi will furnish the University with a Certificate of Insurance ("Certificate of Insurance") evidencing compliance with the insurance provisions of this Agreement. Pepsi's Certificate of Insurance will be delivered to University's representative specified in Section 27 (Notices). Pepsi is required to give University thirty (30) days' advance written notice of any modification, change, or cancellation of any of the insurance coverages.

The insurance requirements set forth in this Section 18 shall not limit Pepsi's liability.

19. **LIMITATION OF LIABILITY**. Neither Party shall be liable for any indirect, consequential, incidental, special, punitive, or exemplary damages of any kind arising out of or in any way related to this agreement, whether in warranty, tort, contract, or otherwise, including, without limitation, loss of profits or loss of good will, whether or not the other Party has been advised of the possibility of such damages and whether or not such damages were foreseeable.

20. **TERMINATION**. A Party may terminate this Agreement immediately upon written notice to the other Party:

(a) in the event of the material breach of any term of this Agreement, which breach remains uncured for a period of thirty (30) days following written notice by the non-breaching Party; or

(b) if the other Party notifies any governmental body of insolvency or pending insolvency, or suspension or pending suspension of operations, or files a voluntary petition in

DocuSign Envelope ID: 5DB6E2D2-8865-4C2F-9B02-F38647AC4CB4

bankruptcy, or is adjudicated as bankrupt or insolvent, or dissolves, winds up or otherwise ceases to actively conduct its business; or

(c) if performance by either Party of its obligations under this Agreement is in violation of any order of court in any proceeding.

In the event of termination of this Agreement pursuant to this Section 20, University shall retain all payments made by Pepsi prior to the date of such termination, and may request additional payments or acceleration of the amounts due, as appropriate.

All provisions which, by their nature, extend beyond the Term will survive termination of this Agreement.

21. **ADDITIONAL RIGHT TO TERMINATE**. Without limiting the rights set forth elsewhere in this Agreement, either Party may terminate this Agreement on sixty (60) days' written notice if: i) the other Party materially changes the nature, purpose or scope of its activities from the nature, purpose or scope of activities as defined in Exhibit A as of the Effective Date in a manner that causes harm or ill repute to the other Party; or ii) either Party materially changes the nature, purpose or scope of its activities from the nature, purpose or scope of its activities as of the Effective Date in a manner that is not consistent with the educational, research and public service mission of University as of the Effective Date; or iii) if any senior executive officer of either Party is formally charged with, or pleads guilty or no contest to a felony or is otherwise involved in a scandal that receives significant media coverage and reflects unfavorably upon the other Party; or (iv) if either Party violates UC labor policies and/or specifically Pepsi is dropped from membership in the FLA. In the event either Party terminates this Agreement pursuant to this Section 21, Pepsi will pay the University for all Services rendered, expenses incurred and non-cancellable obligations as of the date the notice of termination was sent. University will refund to Pepsi any prepaid amounts (a) not earned by University prior to the date of such termination, (b) not applicable to expenses incurred by University prior to the date of such termination and/or (c) not applicable to non-cancellable obligations of University made prior to the date of such termination. In the case of termination by University pursuant to Section 21, University's liability will be limited to the amount of any such refund.

22. **COMPLIANCE WITH LAW**. Each Party shall comply with all applicable laws and regulations in connection with its performance of this Agreement.

23. **PRESS RELEASES AND ANNOUNCEMENTS**. Except as agreed otherwise in writing, neither Party shall issue a press release or other announcement regarding this Agreement or the rights and obligations contemplated by this Agreement without the advance written consent of the other Party.

24. **NO THIRD PARTY BENEFICIARIES.** This Agreement inures to the benefit of the University and Pepsi only, and no third party shall have any rights under this Agreement except as is expressly provided in this Agreement.

25. **RECITALS**. The provisions of the Recitals above are fully incorporated into this Agreement by this reference.

26. **AUTHORITY**. Each Party represents and warrants that it has the necessary power and authority to enter into this Agreement and perform its obligations hereunder.

27. **NOTICES**. Except as otherwise provided in this Agreement, any notice or other communication required or permitted hereunder shall be in writing and shall be deemed to have been duly given (i) on the date of service if served personally, (ii) two (2) business days after deposit with a nationally recognized overnight delivery service, or (iii) five (5) days after mailing if mailed, by first class mail, to the following addresses. Each Party may change its designated address by written notice to the other Party.

If to Pepsi:

Aimee Johns
Workplace Channel Manager
29000 Hesperian Blvd, Hayward CA 94545

Coleen Gravem
Key Account Manager
29000 Hesperian Blvd, Hayward CA 94545

With copy to:

Megan Farr
Pepsi Co, Hayward Facility
Unit General Manager
29000 Hesperian Blvd, Hayward CA 94545

DocuSign Envelope ID: 5DB6E2D2-8865-4C2F-9B02-F38647AC4CB4

If to the University:

Amy Gardner
University of California, Berkeley
University Business Partnerships & Services
1608 Fourth Street, Suite 200, MC 1510
Berkeley, CA 94710

With copy to:

Eryn Hong
University of California, Berkeley
Office of Business Contracts and Brand Protection
Attn: BCBP
1608 4th Street, Suite 228, MC 7600
Berkeley, CA 94710
Berkeley, CA 94710

28. **DATA SECURITY AND PRIVACY REQUIREMENTS**. Pepsi agrees to adhere to the additional terms and conditions regarding data security that are contained in "First Amendment to Appendix – Data Security and Privacy," which is attached as Exhibit F, and hereby incorporated into the Agreement by reference as though fully set forth herein

29. **ASSIGNMENT**. This Agreement shall be binding upon and inure to the benefit of the respective successors and permitted assigns of the Parties; provided, however, that neither Party may transfer or assign its rights or obligations hereunder, by operation of law or otherwise, without the advance written consent of the other Party. Any attempted assignment of a Party's rights or obligations under this Agreement in violation of this Agreement shall be null and void.

30. **FORCE MAJEURE**. Neither Party will be responsible to the other or to any third party for any failure, in whole or in part, to perform any of its respective obligations hereunder, to the extent and for the length of time that performance is rendered impossible or commercially impractical, owing to acts of God, public insurrections, pandemics, epidemics, floods, fires, strikes, lockouts, or other labor disputes, disruptions in supply, shortages or scarcity of materials, changes to applicable laws and regulations and other circumstances of substantially similar character beyond the reasonable control of the affected Party(s) (collectively, "Force Majeure"). Any Party(s) so affected, will (i) use all reasonable efforts to minimize the effects thereof and (ii) promptly notify the other Party(s) in writing of the Force Majeure and the effect of the Force Majeure on such Party's ability to perform its obligations hereunder. The affected Party(s) will promptly resume performance after it is no longer subject to Force Majeure. If the Force Majeure period continues beyond ninety (90) days, the Parties agree to discuss in good faith potential modifications to this Agreement and in the event an agreement cannot be reached, any Party may terminate the Agreement. In the event University's performance is temporarily suspended pursuant to a Force Majeure event, Pepsi's volume-dependent, fixed, advanced, or guaranteed funding obligations will be suspended for the duration of University's nonperformance. Once University resumes performance or in the event University is able to perform some, but not all of its obligations herein, any volume-dependent, fixed, advanced, or guaranteed funding will be adjusted commensurate with the decline in volume associated with the suspended or partial performance.

31. **SEVERABILITY**. Whenever possible each provision of this Agreement will be interpreted in such a manner as to be effective and valid under applicable law, but if any provision of this Agreement will be prohibited, void, invalid, or unenforceable under applicable law, such provision will be ineffective to the extent of such prohibition, voidability, invalidity or unenforceability without invalidating the remaining provisions of this Agreement, which will be given full effect without regard to the invalid provisions.

32. **GOVERNING LAW; JURISDICTION**. This Agreement is controlled by the laws of the State of California, without giving effect to its conflicts of laws principles. Any disputes arising under, resulting from or in connection with this Agreement shall be heard in the court of competent jurisdiction in the County of Alameda, California.

33. **ENTIRE AGREEMENT**. This Agreement constitutes the only agreement, and supersedes all prior agreements and understandings, both written and oral, between the Parties with respect to the subject matter hereof, and all schedules and exhibits attached hereto are a material part of this Agreement and are incorporated herein by this reference. This Agreement is not intended to confer upon any person other than the Parties hereto any rights or remedies hereunder.

34. **INDEPENDENT CONTRACTORS**. The Parties are independent contractors, and nothing in this Agreement will be interpreted as creating an agent-principal, joint venture, partnership, limited liability company, or any legal relationship other than independent contractors.

35. **COUNTERPARTS**. This Agreement may be executed in two or more counterparts, each of which shall be deemed an original, but all of which taken together will constitute one and the same instrument. Execution and delivery of this Agreement by delivery of a facsimile or electronically recorded copy (including a .pdf file) bearing a copy of the signature of a Party will constitute a valid and binding execution and delivery of this Agreement by such Party. Such copies will constitute enforceable original documents.

36. **MODIFICATION**. This Agreement may not be modified unless the modification is contained in a writing signed by the authorized representatives of Pepsi and the undersigned representative of the University or that person's successor.

37. **NO IMPLIED WAIVER**. No delay in exercising or failure to exercise any right shall constitute a waiver of that right, but time is of the essence with respect to the covenants contained herein.

38. **CONSTRUCTION**. Ambiguities, if any, in this Agreement will be reasonably construed in accordance with all relevant circumstances, including, without limitation, prevailing practices in the industry of the Parties in the place where this Agreement is to be performed and will not be construed against either Party, irrespective of which Party may be deemed to have authored the ambiguous provision. The Parties to this Agreement are represented by counsel and have had the opportunity to freely negotiate the terms of this Agreement, so neither shall be deemed the author of this Agreement. Any reference to "days" in this Agreement means calendar days unless otherwise expressly specified.

39. **SUSTAINABILITY**. The rights, permissions, messaging, imagery, Products, promotions, and any other activities or goods provided or performed by Pepsi as part of this Agreement are subject to the sustainability-related provisions outlined in Exhibit H, Sustainability, of this Agreement.

40. **HEALTH AND WELLNESS**. The rights, permissions, messaging, imagery, Products, promotions, and any other activities or goods provided or performed by Pepsi as part of this Agreement are subject to the health and wellness-related provisions outlined in Exhibit I, Health and Wellness, of this Agreement.

41. **ADDITIONAL UNIVERSITY LOCATIONS**. This Agreement sets forth the terms and conditions that apply to provision of the Beverages set forth in Exhibit A by Pepsi to the Campus. As used in this Section 41, "**Additional University Location**" means any location of University not identified in the Agreement, including, without limitation, the Office of the President, the Division of Agriculture and Natural Resources, the Lawrence Berkeley National Laboratory, the University of California campuses and the University of California medical centers. Pepsi agrees that the terms of this Agreement may be extended to Additional University Locations pursuant to addenda mutually agreed to by the Parties that specify the Additional University Location and the other terms applicable to such arrangement. All contractual administration issues (e.g., terms and conditions, extensions, and renewals), operational issues, fiduciary responsibility, payment issues, performance issues and liabilities, and disputes involving individual Additional University Locations will be addressed, administered, and resolved by each Additional University Location. This Agreement does not obligate any Additional University Locations not specified herein under this Agreement or Pepsi as to any Additional University Locations not specified herein.

**THE REGENTS OF THE UNIVERSITY OF CALIFORNIA**	**PEPSICO BEVERAGE SALES, LLC**
By: DocuSigned by: [signature] E38D77A49C6F4CA...	By: DocuSigned by: Coleen Gravem D06078F0FED4475...
Eryn Hong	Coleen Gravem
Its: Associate Director, Brand Protection	Its: Key Account Manager – Workplace
Date: September 28, 2023	Date: September 28, 2023

EXHIBIT A

PEPSI'S PRODUCTS, SERVICES AND APPROVED DESIGNATIONS

For the purpose of this Agreement only, in order to define the exclusivity to be provided by University, Pepsi's business is defined as the manufacture and distribution of cold Beverages.

Approved Designations

For the purposes of this Agreement, Pepsi may utilize the following designations when describing or referring to the rights provided to Pepsi pursuant to this Agreement:

"Official Beverage Partner of UC Berkeley"

"Official Beverage Partner of Cal Athletics"

In addition to the earlier stipulations of this Agreement, Pepsi's provision of Beverages to the University, and utilization of the designation(s) noted immediately above, is governed by the terms of this Exhibit A.

(A) **Exclusivity**. Except for the **Permitted Exceptions** and other exclusions set forth in this Agreement, Pepsi will be the exclusive Beverage supplier to, and Beverage sponsor of, University. University will take all reasonable steps to ensure that the Beverages are the exclusive products of their respective types (carbonated and non-carbonated, non-alcoholic drinks, however dispensed except as set forth herein) sold, dispensed or otherwise made available, or in any way advertised, displayed, represented or promoted at or in connection with the Participating Food Service Locations identified in Exhibit K and applicable Teams by any method or through any medium whatsoever (including without limitation digital, print, broadcast, direct mail coupons, handbills, displays and signage), whether public or private.

i. Permitted Exceptions:

a. Shelf Space Exception: Subject to the Permitted Exceptions stipulations, the University may allocate 15% of retail shelf space to Competitive Products.

b. Teams: Subject to the Permitted Exceptions of, and other exclusions set forth herein, this Agreement, during the Term, the Teams shall not themselves nor shall they authorize a third party to promote, market advertise, sponsor or endorse any Competitive Products in connection with the University, the Teams, or the Teams' Marks unless the University has offered the opportunity in writing to Pepsi to contract for such marketing, advertising, or sponsorship opportunity on commercially reasonable terms consistent with the fair market value for such opportunity and (i) Pepsi has declined to participate in such opportunity; or (ii) Pepsi has refused to enter into a contract to participate in such opportunity on commercially reasonable terms consistent with the fair market value of such opportunity (such declining or refusal following University offering will be described herein as a "Pepsi Walkaway"). This provision shall be applicable upon the expiration of existing marketing agreements between Pepsi (or its subsidiaries) and the University with regard to Gatorade's and Muscle Milk's exclusive sponsorship in the categories of sports drinks and protein of the Teams set to expire on June 30, 2027. Even if a Pepsi Walkaway occurs, the University may not, under any circumstances, sell, serve, promote, market advertise, merchandize, publicly sponsor or endorse Competitive Products at the Facilities other than at the athletic facilities. In the event of a Pepsi Walkaway, the University may sell marketing, promotion, and advertising opportunities that include promotional opportunities within the athletic facilities and may distribute at no cost Competitive Products of such sponsor to its Teams and its players, coaches and staff. At no time shall the terms of this Section be read to apply to any other products.

c. Coffee: For the avoidance of doubt, both Parties acknowledge that the University has an agreement with Peet's Coffee, Inc. ("Peet's") dated July 1, 2017 for Peet's exclusive sponsorship in the category of coffee.

d. Non-alcoholic (or dealcoholized) Beer, Wine, and Spirits: Pepsi does not currently manufacture or distribute other nonalcoholic beer, wine and spirits products. In the event University would like to enter into an agreement with another manufacturer of such beverages, University agrees to give Pepsi a first right of refusal before it enters into such an agreement.

e. GT Living Foods: For the avoidance of doubt, both Parties acknowledge that Teams have a non-

exclusive three (3) year agreement with GT Kombucha set to expire on June 30, 2026 for Teams sponsorship in the category of kombucha.

ii. Direct Competitor: The University will not serve, dispense, or otherwise make available or permit the availability of, or in any way advertise, display, represent or promote, beverage products licensed by, or produced by bottlers licensed by, The Coca-Cola Company or any affiliate thereof. Notwithstanding the foregoing, upon expiration of University's agreements with Gatorade and Muscle Milk, the Teams may enter into an agreement with The Coca-Cola Company for hypertonic, isotonic and hypotonic drinks (sports drinks and fluid replacements) in the event University has offered the opportunity in writing to Pepsi to contract for such marketing, advertising, or sponsorship opportunity on commercially reasonable terms consistent with the fair market value for such opportunity and (i) Pepsi has declined to participate in such opportunity; or (ii) there is a Pepsi Walkaway.

(B) Purchase and Resale of Products. University agrees that Participating Food Service Locations will purchase Packaged and Post-Mix Products exclusively and directly from Pepsi, subject to the Permitted Exceptions noted above, and subject to the applicable components of the sustainability and health-and-wellness stipulations outlined in Exhibits H (Sustainability) and I (Health and Wellness) of this Agreement. Throughout the Term, University will continuously serve, dispense, sell and/or otherwise make Products available throughout the Participating Food Service locations. University agrees to pay all accounts owing to Pepsi in accordance with payment terms as established by Pepsi, provided that:

- these terms include electronic invoicing and allow electronic reconciliation, and that;
- Pepsi cooperates with the integration of its applicable operations and platforms into the University's menu management and inventory system(s) to facilitate real-time inventory control, and that;
- University units be given significant account "autonomy," so that the procurement and payment activities of one unit do not hinder other units' opening of accounts, ordering or receiving Products based on the status ("delinquent," "current," etc.) of other University accounts.
    - This account "autonomy" does not excuse the University of payment obligations.

(C) **Fountain Products**. University agrees to use the Postmix Products for use in preparing the fountain beverage products (the "***Fountain Products***"): (i) in accordance with the standards established by Pepsi and (ii) only for immediate or imminent consumption; University not to resell the Postmix Products either to nonaffiliated outlets or to consumers in any form other than the Fountain Products.

(D) **Ancillary Product**. University agrees that Participating Food Service Locations will purchase hot and cold cups and lids that must be fiber-based, free of intentionally added polyfluorinated chemicals (PFAS-PFOS) and BIP or CMA-certified as biodegradable and compostable. Only natural fiber-based items, or fiber-based items lined with a BPI or CMA-certified PLA, are accepted ("***Ancillary Products***"). Pepsi-branded ancillary products shall be purchased exclusively from Pepsi provided Pepsi's offerings meet the aforementioned criteria. University can purchase any non-Pepsi-branded ancillary products, and will not brand ancillary products with other Beverage providers' branding, meeting University requirements from Suppliers that can provide product that meets University requirements.

(E) **Product Mix; Minimum SKU/Brand Requirement**. Throughout the Term, University agrees to offer a mutually agreed-to mix of Pepsi's Beverage products and package sizes and types for sale and distribution throughout the Participating Food Service Locations, provided that this mix allows University to comply with the policies identified, and Pepsi to comply with the requirements outlined in Exhibits H (Sustainability) and I (Health and Wellness) of this Agreement. For the avoidance of doubt, both Pepsi and the University acknowledge and agree that these policies include the University of California Sustainable Practices Policy, and the Berkeley campus Food and Beverage Choices Policy, and that the University, with Pepsi's agreement, will be able to offer for sale only "Healthier" beverages, as defined by the Food and Beverage Choices Policy, at certain Participating Food Service Locations throughout the Term of this Agreement. Unless otherwise agreed upon in this Agreement, *Pepsi and the University further acknowledge and agree that effective **January 1, 2024**, Pepsi will not sell any Products in single-use plastic packaging to the University, as defined by the University in Exhibit H of this Agreement, with the exception of its Gatorade and Naked Juice product lines. With regard to these two product lines, Pepsi will offer non-single use plastic versions of these products to the University as soon as such products are available. Pepsi and the University further acknowledge and agree that, effective **January 1, 2030**, Pepsi will not sell or otherwise provide any Products or other items in single-use plastic packaging, as defined by the University in Exhibit H of this Agreement, to the University.*

(F) **Restrictions for Products**. The parties recognize and agree that there are certain additional territorial restrictions that pertain to the purchase and resale of the Products. To the extent any prospective

Facilities are located outside the territories serviced by Pepsi, then Pepsi may, upon request by University, use commercially reasonable efforts to facilitate an agreement between the Pepsi bottler servicing the applicable territory and University with terms substantially similar to the terms of this Agreement. Furthermore, University agrees not to distribute or resell the Products, directly or indirectly, outside the territories serviced by Pepsi and shall cause its purchasing representative to abide by such territorial restrictions.

(G) **Food Service Providers**. The terms and conditions of this Agreement, including the pricing, funding and other consideration provided for herein is based on University's current operating model as of the Effective Date of this Agreement. If University is self-operated and converts to a food service provider model during the Term, then University must require such food service provider to abide by the applicable pricing and other terms set forth in this Agreement to the exclusion of all other benefits, including in connection with any agreement such food service provider may separately have with Pepsi or Pepsi's affiliates. In the event that University fails to adhere to this requirement (or the food service provider refuses to abide accordingly), then Pepsi shall be entitled to adjust its pricing, funding or other consideration offer to University herein on an equitable basis to neutralize any negative impact such change may have on the economics of the original Agreement.

(H) **Best Taste Limit and Product Handling**. University understands that the Products provided hereunder are provided with a best taste limit ("BTL") date printed on the packaging. Neither Pepsi nor the bottlers replace Products that are past the BTL date. University agrees that no Product shall be sold past the BTL date, and that it shall abide by company procedures on product handling and quality control periodically published by the manufacturer.

(I) **Equipment and Service.**

(1) Pepsi shall, based upon Pepsi's and the University's survey of the Participating Food Service Locations' needs, provide and install all Equipment at the Participating Food Service Locations for the dispensing of Products during the Term at no cost to University. Before Year 4 of the Agreement and provided Pepsi determines that it is both commercially reasonable and feasible, Pepsi will refresh or replace all fountain equipment of the Participating Food Service Locations. The rest of the Participating Food Service Locations' equipment will be refreshed or replaced as needed.

(2) Pepsi and the University will commit to a mutually-agreed upon preventive maintenance program and schedule for the Equipment, wherein Pepsi will perform the preventive maintenance activities according to the schedule outlined in Section (L) below.

(3) Title to all Equipment shall be with Pepsi or its affiliates. All Equipment will be either new or certificated to be in "like new" condition, and shall be Energy Star rated. University retains the right to refuse Equipment per University reasonable standards for its Participating Food Service Locations. Pepsi will loan to University, at no charge, appropriate Equipment for dispensing the Products at the Participating Food Service Locations. University agrees that the equipment will be exclusively used to display and merchandise the Products as reasonably determined by Pepsi, and subject to applicable local law, rule, or regulation. University will not use the Equipment to display, stock, advertise, sell or maintain any other products (including on the exterior of the Equipment). Title to such Equipment will remain vested in Pepsi or its affiliate and University will return all Equipment to Pepsi upon expiration or earlier termination of this Agreement. During the Term, Pepsi will provide, at no charge to the University, preventive maintenance and service to the Equipment. Pepsi will service and stock, if necessary, (i) the Equipment and (ii) any additional Equipment determined by the parties to be installed at new Participating Food Service Locations. Pepsi shall provide University with a telephone number to request emergency repairs and receive technical assistance related to the Equipment. Pepsi shall respond to each University request and use reasonable efforts to remedy the related Equipment problem as soon as possible,. University may request emergency repairs and receive technical assistance related to the Equipment by contacting Pepsi's service hotline at 877-386-4567. 24/7 access is also available through digital service platforms. www.pepsiequipmentservice.com and/or www.pepsicopartners.com. Pepsi shall use commercially reasonable efforts to respond to each request for emergency service within 24 hours during normal dispatch hours of 7 AM to 8 PM Monday-Friday. Non-emergency service requests shall be responded to within three (3) days and completed as soon as commercially reasonable. Non-operational service requests shall be given priority over general service requests. Any excessive service issues or delays shall be timely reported to Pepsi's key account manager and Pepsi shall take commercially reasonable efforts to resolve such issues

DocuSign Envelope ID: 5DB6E2D2-8865-4C2F-9B02-F38647AC4CB4

to University's reasonable satisfaction. In the event there is no such resolution, then University and Pepsi will enter into mutually agreeable discussions including, but not limited to, a reduction in the Annual Units Threshold for the affected Year.

(4) To the extent that future technology enhancements, equipment platforms or products to support these platforms are substantially different in scope of composition compared to existing equipment components and products, Pepsi will work with the University on campus roll out opportunities and University will work in good faith to negotiate the economic terms for implementation of the new technology equipment. The Equipment may not be removed from the Participating Food Service Locations without Pepsi's written consent, and the University agrees not to encumber the Equipment in any manner or permit other equipment to be attached thereto except as authorized by Pepsi. Upon expiration or earlier termination of the Term, all Equipment will be surrendered to Pepsi. University shall submit equipment needs and may request to change the product mix, add or reduce dispensing units, or direct the removal of Equipment. Removal of Equipment from the Participating Food Service Locations by Pepsi shall be done at no expense to University. Pepsi shall ensure Vending Machines are equipped with non-resalable counters for tracking sales by product and total dollars. Vending Machines shall be serviced and stocked frequently to ensure a wide selection of products are available , and shall have web-based diagnostics for stock levels and maintenance requirements. If a failure to stock or service the Vending Machines persists, then University and Pepsi will enter into mutually agreeable discussions including, but not limited to, a reduction in the Annual Units Threshold for the affected Year. Pepsi shall employ a driver with the responsibility of understanding the campus schedule and stock accordingly, and deliver per a schedule agreed to by University and Pepsi. Pepsi shall be responsible for collecting, for its own account, all cash monies from the Vending Machines and for all related accounting for all cash monies collected therefrom. Pepsi or its designated agents shall be responsible for maintaining, repairing, and replacing the Equipment. At the end of the Term, Pepsi shall have the right to, and shall upon request of the University, remove all Equipment from the Participating Food Service Locations at no expense to the University.

(5) Upon expiration of this Agreement, if University has not entered into a further agreement with Pepsi for the purchase of the Products, University will surrender to Pepsi all Equipment installed at the Participating Food Service Locations.

(J) **Vending Machines**. With respect to the vending machine Equipment placed at the Facilities (the "***Vending Machines***"), specific placement of said machines will occur through mutual agreement. Pepsi will have the additional responsibility for (i) stocking the Vending Machines with the Products and (ii) collecting, for its own account, all cash monies from the Vending Machines and for all related accounting for collected monies. University agrees to provide reasonable assistance to Pepsi in apprehending and prosecuting vandals. Pepsi shall not be obligated to pay Commissions on documented revenue losses resulting from vandalism or theft of Product with respect to any Vending Machines. Pepsi shall not be assessed common area maintenance fees, taxes or other charges based on its occupation of the space allocated to its Equipment at the Facilities. If Pepsi removes or moves any Vending Machines upon mutual agreement, Pepsi will confirm the removal or move with the designated University contact. On an annual basis, Pepsi will provide an updated list of Vending Machine types and locations to designated University contact. All Vending Machines will have the opportunity to display a University face-front graphic and will include designated space for University messaging. Cost for providing custom fronts will be covered out of existing funds provided to the University by Pepsi. If mutually agreed to, Pepsi will provide printed graphic interior building signage, reviewed and approved by University, to direct users to vending locations. Pepsi shall work with University to identify optimal locations for such equipment; and provided, further, that the University shall have the right to identify existing vending machine locations for the installation of Pepsi's vending machines. Pepsi agrees the University or any of its duly authorized representatives at any time during the Term of the Agreement an upon written request by the University shall have access to, and the right to audit and examine, any pertinent books, documents, papers and records relating to the performance of the Agreement for Vending Machines. Pepsi acknowledges and agrees that where commercially reasonable to do so, University prefers that vending machine fronts promote "Healthier" beverages, as defined by the Berkeley campus Food and Beverage Choices Policy. University agrees to work with Pepsi to relocate Vending Machines that have consistently low sales volume to more optimal, higher traffic locations, as mutually agreeable. Pepsi may, upon mutual agreement with the University, remove Vending Machines that have consistently low sales volume or are otherwise less than optimal pursuant to Pepsi's and the University's determination. Upon such removal, University and Pepsi will enter into mutually agreeable

discussions including, but not limited to, a reduction in the Annual Units Threshold for the affected Year.

(K) **Payment Processor**. Pepsi agrees to provide credit card readers for all Vending Machines that Pepsi places at the Facilities that include credit/debit card readers, and that Pepsi shall require its credit card processing vendor to maintain a strict PCI compliant program. Pepsi shall retain ownership and responsibility for all credit/debit card readers and responsibility for vendor's compliance with Payment Card Industry (PCI) standards. Pending the University's consent, Pepsi shall work with University to incorporate, at no charge, the University's local one-card payment system. Pepsi agrees to provide, upon written request from University, current evidence, in form and substance reasonably satisfactory to University, of compliance with Payment Card Industry Security Council data security standards, which has been properly certified by an authority recognized by the payment card industry for that purpose.

(L) **Service.** Pepsi will provide, at no charge to the University, preventive maintenance for the Equipment it provides the University during the Term of this Agreement in accordance with the following schedule:

- Semi-annual beverage line flush for fountain drink machines. Replacement shall be done if the lines are broken or leaking;
- Semi-annual check on drink calibration and set appropriate drink mix ratio for fountain drinks for fountain machines;
- Quarterly audit/maintenance and parts replacement of all beverage dispensers, tea machines, bubbly machines, BIB dink connectors, valves, spigots, nozzles and retail cold merchandisers, merchandiser doors & lights, and other applicable Pepsi Equipment if such items are not working properly;
- Twenty-four (24)-hour response time to troubleshooting and repairing for equipment breakdown service calls;
- Replacement of any Equipment that is ineligible or unsuitable for repair within one (1) month;
- Quarterly maintenance report regarding Equipment serviced, issues noted, etc. to be provided applicable University contact(s).

Preventive maintenance will be performed on an appropriate ongoing basis throughout Participating Food Service Locations and the broader Facilities to maintain the Equipment in the proper condition to consistently perform as designed. In particular, the University and Pepsi will coordinate Pepsi's maintenance activities and access to the University's Participating Food Service Locations and broader Facilities during appropriate University breaks (winter, spring, summer, etc.), downtimes, etc.

Pepsi will otherwise service the Equipment as appropriate to maintain its functionality throughout the Participating Food Service Locations and broader Facilities. Pepsi will also provide University with a telephone number to request emergency repairs and receive technical assistance related to the Equipment after business hours. Pepsi representatives (inclusive of those stocking, repairing, responding to service calls, sales, etc.) will physically report to the Manager on duty prior to and upon completion of services to ensure mutual communication and expected services were successfully performed. Notwithstanding the foregoing, University acknowledges that delays in service may be caused by factors well outside of Pepsi's control, and therefore Pepsi's service record will be measured in the aggregate such that an isolated failure is not a material breach of the Agreement. Pepsi will provide service records to the University. Pepsi is responsible for keeping the Vending Machines stocked and for Product availability in all venues. If the University has actual knowledge that Pepsi has not reasonably stocked the Vending Machines or that there is a lack of Product at the Facilities which Pepsi failed to cure, and if the University reasonably believes that such failure to stock or lack of Product might impact sales volume, then the University shall be obligated to report such failure to stock or lack of Product as soon as reasonably possible after the University obtains actual knowledge of such issue. Pepsi will promptly respond to such situations so as to remedy any out-of-stocks. If such failure to stock the Vending Machines or maintain ample levels of inventory for Dining and Retail persists, then University and Pepsi will enter into mutually agreeable discussions including, but not limited to, a reduction in the Annual Units Threshold for the affected Year. The parties acknowledge and agree that nothing contained in this Section shall relieve Pepsi of its responsibilities to stock and maintain Product availability at all times during the Term. Pepsi will be responsible for providing sufficient personnel and support services to ensure timely maintenance of vending machines to the satisfaction of the University. The machines shall be serviced frequently enough to ensure a wide selection of products are available. In the event a vending machine cannot be maintained in a fully operational status, that machine shall be replaced with an equivalent machine to ensure continuity of vending services. All machines shall display a phone number

DocuSign Envelope ID: 5DB6E2D2-8865-4C2F-9B02-F38647AC4CB4

to be used for reporting a machine that is malfunctioning/inoperable. Pepsi will be responsible for maintaining its vending equipment and products in a manner consistent with acceptable vending trade practices that will enhance product marketability and ensure the maximum levels of services from all such vending equipment installed at the Facilities. To that end, Pepsi will aim to provide and install wireless monitors with web-based dashboards indicating stock and maintenance to avoid out-of-stock occurrences and malfunctioning machines.

(M) **Access to University Facilities and Participating Food Service Locations**. Pepsi's access to the University Facilities and Participating Food Service Locations, in connection with the Agreement, shall only be in accordance with all applicable federal, state, and local laws and University policies, which may include individuals signing waivers of liability and assumption of risk prior to accessing such Facilities and Participating Food Service Locations. Access to and/or use of the Facilities and the Participating Food Service Locations may be denied in the University's sole discretion if circumstances are such that the proposed use or access would interfere with the orderly operations of the University's programs. Pursuant to the University policies, anyone who will be present on University campus will be required to meet campus, city, and state mandates relating to COVID-19, including physical distancing, face coverings and hand washing hygiene protocols, and the University's Office of Environmental Health & Safety COVID-19 specific trainings, rules and requirements (collectively "COVID-19 Mandates"), as commercially reasonable. Access to campus and/or use of the Facilities may be denied or terminated in the University's sole discretion, if circumstances are such that the access or proposed access or use does not or would not comply with COVID-19 Mandates or interferes, or would interfere, with the orderly operations of the University's programs.

(N) **Pricing.** University will require that participating Food Service Locations purchase Products and Pepsi-Branded Ancillary Products directly and exclusively from Pepsi pursuant to the pricing, exceptions, and other terms and conditions set forth herein. The initial pricing schedule for Products is set forth in attached Exhibit E. Thereafter, the prices may be increased each Year, though the price increases for Ancillary Products, Packaged Products and Postmix Products shall not exceed four percent (4%) per Year. Before a price increase occurs, Pepsi shall provide thirty (30) days' written notice of any such increase. Pepsi will be entitled to pass-through any incremental fees, deposits, taxes or other governmentally-imposed charges (whether local, state, federal or judicially-imposed on manufacturers, distributors, consumers, or otherwise). The pass-through of any such governmentally-imposed fees, deposits, taxes or charges on the Products will not be subject to any pricing cap or notification restrictions that may be specified in this Agreement.

(O) **Price Discrepancy**. Any price discrepancy claim must be submitted to Pepsi within 365 days of the date of the invoice in question. If University makes a price discrepancy claim within 90 days of the invoice date, University must submit a written request specifying the particular Product, amount in dispute and reason for the dispute. This request should be addressed to:

Accounts Receivable
Pepsi-Cola Customer Service Center
P.O. Box 10
Winston-Salem, North Carolina 27102

If University makes a price discrepancy claim from 91 to 365 days after the date of invoice, in addition to the written request as specific above, University must submit to Pepsi a copy of the invoice in question, copies of any check remittances pursuant to the invoice in question and any additional supporting documentation.

(P) **Distribution Limitations**. Pepsi reserves the right to limit quantities, withhold or deduct funding as an offset to amounts not paid by University or terminate this Agreement if University (i) sells Products directly or indirectly for resale outside of Pepsi's exclusive territory where the Facility operates and (ii) purchases Products outside Pepsi's exclusive territory where the Facility operates and resells such Products within Pepsi's exclusive territory.

PEPSICO Foodservice

2023 PEPSI BEVERAGES PORTFOLIO - NorCal

PROUCT INFORMATION www.pepsiproductfacts.com – Products may vary by Market/Location

Coleen Gravem (510) 750-7535

CSD

20oz Bottles (24pk) & 1L Bottles (15pk) & 12oz Cans (24pk)

Pepsi
Diet Pepsi
Pepsi Zero Sugar
Pepsi Zero Sugar Cherry
Pepsi Zero Sugar Mango
Pepsi Zero Sugar Vanilla
Pepsi Real Sugar
Pepsi Mango
Pepsi Vanilla
Pepsi Wild Cherry
Diet Wild Cherry Pepsi
Caffeine Free Pepsi
Caffeine Free Diet Pepsi
Starry
Starry Zero Sugar
Manzanita Sol
Brisk Zero Sugar Lemon
Mtn Dew
Diet Mtn Dew
Mtn Dew Zero Sugar
Mtn Dew Code Red
Mtn Dew Major Melon Zero Sugar
Mtn Dew Major Melon
Mtn Dew Baja Blast
Mtn Dew Baja Blast Zero
Mtn Dew Frost Bite
Mtn Dew Baja Caribbean Splash
Mtn Dew Baja Passionfruit Punch
Mtn Dew Spark
Mtn Dew Spark Zero Sugar
Mug Root Beer
Crush Orange
Crush Orange Zero
Crush Grape
Crush Strawberry
Crush Pineapple
Crush Watermelon

12oz Glass (24pk)
Pepsi Real Sugar

DISTRIBUTION VARIES BY REGION
Dr. Pepper
Diet Dr. Pepper
Dr. Pepper Zero
Dr. Pepper Zero Cherry
Dr. Pepper Cherry
Diet Dr. Pepper Cherry
Dr. Pepper Zero Cream Soda
Dr. Pepper Cream Soda
Diet Dr. Pepper Cream Soda
Dr. Pepper Strawberry Cream

13.65oz Cans (12pk or 4pk)
Pepsi Nitro Cola
Pepsi Vanilla Nitro Cola

7.5oz Cans (10pk / 24pk)
Pepsi
Pepsi Wild Cherry
Diet Pepsi
Pepsi Zero Sugar
Pepsi Real Sugar
Starry
Tonic
Tonic Zero Sugar
Club Soda
Mt Dew
Ginger Ale
Dr. Pepper
Diet Dr. Pepper
Crush Orange
Mug

Mtn Dew Kickstart
16oz Cans (12pk)
Blueberry Pom
Midnight Grape
Mango Lime
Black Cherry
Pineapple Orange Mango
Fruit Punch
Orange Citrus
Neon Base

12oz Sleek Cans (6pk)
Pineapple Orange Mango
Black Cherry
Orange Citrus
Midnight Grape

CRAFT SODA

Bundaberg
375ML Glass Bottles (4pk)
Ginger Beer
Root Beer
Diet Ginger Beer
Blood Orange
Guava
Peach

ENERGY

Celsius
12oz Sleek Cans (12pk)
Sparkling Orange
Sparkling Kiwi Guava
Sparkling Watermelon
Sparkling Peach Vibe
Sparkling Tropical Vibe
Sparkling Arctic Vibe
Sparkling Strawberry Lemonade
Sparkling Wild Berry
Sparkling Fuji Apple Pear
Sparkling Strawberry Guava
Sparkling Grape Rush
Sparkling Lemon Lime (P1W1)
Sparkling Fantasy Vibe (P1W1)
Green Tea Raspberry Acai
Green Tea Peach Mango

Mountain Dew Energy
16oz Cans (12pk)
Pomegranate Blue Burst
Strawberry Melon Spark
Orange Breeze
Peach Mango Dawn
Tropical Sunrise
Berry Blitz
Cherry Lime Lift
Pitch Black LTO (P1W1)

16oz Cans (4pk)
Strawberry Melon Spark
Pomegranate Blue Burst

AMP
16oz Cans (12pk)
AMP Original

Gatorade Fast Twitch (P2W1)
12oz Bottles (12pk)
Watermelon Strawberry
Cool Blue
Glacier Freeze
Tropical Mango
Strawberry Lemonade
Orange

Rockstar
16oz Cans (12pk)
Original Energy
Punched
Sugar Free
Zero Carb
Recovery
Recovery Orange
Recovery Str Lem P1W2)
Pure Zero Punched
Pure Zero TMGS
Pure Zero Grape
Pure Zero Silver Ice
Pure Zero Mandarin Orange
Pure Zero Watermelon
Pure Zero Mango Guava
Pure Zero Lime Cucumber
Pure Zero Watermelon
Pure Zero Strawberry Peach (P1W2)
Boom Whipped Orange
Boom Whipped Strawberry
Revolt Killer Citrus
Hardcore Apple
Xdurance Cotton Candy
Xdurance Super Sour Green Apple
Xdurance Blue Raspberry
Xdurance Ripped
Juiced Pineapple Org Guava
Juiced Island Mango
Blackberry Goji
Orange Island Fruit
Punched Berry Sangria
Punched Tropical Berry
Punched Strawberry Peach (P1W2)
Frozen Lemonade
Punched Watermelon
Aguas Frescas Punched Strawberry (X North)
Aguas Frescas Punched Pineapple (X North)
Recovery 2.0 Mango Lemonade
Recovery 2.0 Pineapple Coconut

Rockstar Unplugged
12oz Sleek Cans (12pk)
Blueberry
Passion Fruit
Raspberry Cucumber (West only)

READY TO DRINK COFFEE

Starbucks Frappuccino
13.7oz Glass Bottles (12pk)
Vanilla
Mocha
Caramel
Coffee

9.5oz Glass Bottles (15pk)
Vanilla
Mocha
Caramel

Starbucks Latte
14oz PET Bottle (12pk)
Caramel Macchiato
Vanilla Latte

Starbucks NITRO
9.6oz Can (12pk)
Black Unsweetened
Vanilla Sweet Cream
Dark Caramel
Dark Cocoa Sweet Cream
Splash of Sweet Cream

Starbucks Pink/Paradise Drink
14oz Plastic Bottles (12pk)
Strawberry Acai
Pineapple Passionfruit

Starbucks Tripleshot Energy
15oz Cans (12pk)
Vanilla
Mocha
Caramel
Zero Sugar Vanilla
Zero Sugar Black
Zero Sugar Milk Chocolate

Starbucks Double Shot Energy
15oz Cans (12pk)
Mocha
White Chocolate
Hazelnut
Vanilla
Caramel
Coffee

Starbucks Cold Brew
11oz Glass Bottle (12pk)
Unsweet Black
Vanilla Sweet Cream

Starbucks Double Shot Espresso
6.5oz Cans (12pk)
Cubano
Salted Caramel
Americano
Espresso Light (24pk)
Espresso Regular (24pk)

Starbucks Cold & Crafted
11oz Glass Bottles (12pk)
Coffee + Splash of Milk & Vanilla
Coffee + Splash of Milk & Mocha

PEPSICO Foodservice

2023 PEPSI BEVERAGES PORTFOLIO

PROUCT INFORMATION www.pepsiproductfacts.com – Products may vary by Market/Location

Last Updated 12/5/2022

HYDRATION

Aquafina Water
16.9oz Bottles (24pk)
20oz Bottles (24pk)
1 Liter Bottles (12pk)
1.5 Liter Bottles (12pk)
16oz Alumitek Bottle (24pk)

LifeWTR
20oz Bottles (24pk)
700ml Sport Cap Bottles (12pk)
1 Liter Bottles (12pk)
1 Liter Bottles (6pk)
500mL Bottles (6pk)
1.5L Bottles (12pk)
1L Immune Support

Proud Source
12oz Can Still
12oz Can Sparkling
16oz Aluminum Bottle Still
16oz Aluminum Bottle Sparkling
750ml Alum Bottle Still
750ml Alum Bottle Sparkling

Bubly Sparkling Water
12oz Cans (24pk)
Blackberry
Lime
Cherry
Grapefruit
Raspberry
Strawberry
Watermelon
Pineapple
Mango
Blueberry Pomegranate
Key Lime Pie
Peach
Coconut Pineapple
Passionfruit
Orange Cream
Lemon Sorbet
Just Bubly

16oz Cans (12pk)
Lime
Grapefruit
Cherry
Blackberry

Bubly Bounce Sparkling Water
12oz Cans (24pk)
16oz Cans (12pk)
Mango Passion Fruit
Triple Berry
Blood Orange Grapefruit
Citrus Cherry
Blueberry Pomegranate

Schweppes
1 Liter Bottles (12pk)
Zero Sugar Tonic
Tonic
Club Soda

VALUE ADDED PROTEIN

Muscle Milk
14oz Bottles (12pk)
Chocolate
Vanilla Crème
Strawberry & Cream
Banana Cream
100 SF Chocolate
100 SF Vanilla
Pro 40 Knockout Chocolate Pro
40 Intense Vanilla
Pro 40 Go Banana
Pro 40 Cookies and Crème
Pro 40 Chocolate Peanut Butter

Evolve
11.16oz Bottles (12pk)
Double Chocolate
Vanilla Bean

Gatorade Zero w/ Protein
16.9oz Bottles (12pk)
Cool Blue
Fruit Punch
Glacier Cherry

GATORADE

Gatorade
20oz Bottles (24pk)
Fruit Punch
Lemon-Lime
Orange
Glacier Freeze
Fierce Grape
Blue Raz
Lime Cucumber
G2 Grape
Frost Glacier Cherry
Fierce Blue Cherry
Fierce Apple
Flow Blackberry
Flow Strawberry Kiwi

12oz Bottles (24pk)
Glacier Cherry
AS Orange
AS Berry

28oz Bottles (15pk)
Glacier Freeze
Lemon Lime
Fruit Punch
Blue Raz
Orange
Fierce Grape
Glacier Cherry
Fierce Blue Cherry
Fierce Strawberry
Lime Cucumber
Passion Fruit
Frost Arctic Blitz
Fierce Green Apple
Riptide Rush
Kiwi Strawberry
Icy Charge

Protein Bars
2.8oz Bars (12pk)
Chocolate Chip
Choc. Chip Pnt Bttr
Chocolate Caramel

Gatorade ZERO
20oz Bottles (24pk)
Glacier Cherry
Glacier Freeze
Lemon Lime

28oz Bottles (15pk)
Cool Blue
Glacier Cherry
Berry
Orange
Lemon Lime
Glacier Freeze
Fruit Punch
Lime Cucumber
Citrus Kick (P10W1)

Gatorade Fit (P3W1)
28oz Bottles (15pk)
Tropical Mango
Citrus Berry
Watermelon Strawberry
Cherry Lime
Blackberry Raspberry (P3W1)

Gatorade Gatorlyte
20oz Bottles (12pk)
Orange
Strawberry Kiwi
Zero Sugar Strawberry Kiwi
Zero Sugar Lemon Lime
Cherry-Lime
Watermelon
Mixed Berry
Lime Cucumber

Propel
1L Bottles (12pk)
Grape
Kiwi Strawberry
Berry

Propel Immune Support
20oz Bottles (24pk)
Orange Raspberry
Lemon Blackberry

Propel Zero
20oz Bottles (24pk)
Kiwi Strawberry
Berry
Grape
Strawberry Lemonade
Black Cherry
Watermelon
Mango

TEA

Lipton Brisk
12oz Cans (24pk)
Lemon
Lemon Zero
Raspberry
20oz Bottles (24pk
Lemon

Lipton Iced Tea - LIT
20oz Bottles (24pk)
Citrus Green
Citrus Green Dt
Peach
Southern Sweet
Texas Sweet

Lipton Pure Leaf Brewed Iced Tea
18.5oz Bottles (12pk)
Sweet
Unsweet
Extra Sweet
Lemon
Raspberry
Sweet Green
Peach
Lemonade
Mango Hibiscus
Unsweet Green Tea
Unsweet Lemon
Diet Lemon
Honey Green
Subtly Sweet
Subtly Sweet Peach
Subtly Sweet Lemon

16.9oz Bottles (12pk)
Unsweet
Sweet
Extra Sweet
Raspberry
Immune Support-Pineapple Mango
Immune Support-Dt Pineapple Mango
White Tea Raspberry (P2W4)

16.9oz Bottles (6pk)
Subtly Sweet
Subtly Sweet Peach

Yachak Yerba Mate (West Div Only)
16oz Can (12pk)
Berry Red
Berry Blue
Ultimate Mint
Passionfruit
Blackberry

PEPSICO Foodservice

2023 PEPSI BEVERAGES PORTFOLIO

PROUCT INFORMATION www.pepsiproductfacts.com – Products may vary by Market/Location

Last Updated 12/5/2022

JUICE *(Shelf Stable)*

Ocean Spray Juice
15.2oz Bottles (12pk)
Cranberry
Cran-Grape

Robinson's Fruit Shoots
10.1oz Bottles (24pk)
(*No Sugar Added)
Berry Burst
Strawberry Raz

Dole Juice
15.2oz Bottles (12pk)
100% Apple Juice
100% Orange Juice
100% Ruby Red Grapefruit
Strawberry Kiwi
Tropical Fruit Punch

Tropicana Juice
32oz Ambient (12pk)
Orange
Apple Cranberry

Dole Lemonade
20oz Bottles (24pk)
2L Bottles (8pk)
Lemonade
Strawberry Lemonade

BAR

Neon Zebra
7.5oz (24pk)
Margarita
Strawberry Daiquiri
Mojito
Whiskey Sour

3 Gallon Bar Mixers
Red Alert
Margarita Ginger
Beer Sour Mix

FOUNTAIN BEVERAGES

Post Mix
5 Gallon BIB
Pepsi
Diet Pepsi
Mountain Dew
Wild Cherry Pepsi
Starry
Crush Orange
Mug Root Beer
Dr. Pepper *varies regionally*
Diet Dr. Pepper
Tropicana Pink Lemonade
Tropicana Lemonade
Brisk Unsweet Tea
Brisk Raspberry Tea

3 Gallon BIB
Pepsi Zero Sugar
Lipton Iced Tea
Lipton Sweet Tea
Schwepps Ginger Ale
Schwepps Tonic
SoBe Life Water Yumberry Pomegranate
Diet Mt. Dew
Gatorade Fruit Punch
Gatorade Lemon Lime
Tropicana Fruit Punch
Dole 100% Apple Juice
Dole 100% Orange Juice
Dole 15% Cranberry Juice

CRAFT Stubborn Soda
3 Gallon BIB
Draft Cola
Draft Cola Zero Sugar
Classic Root Beer
Lemon Berry Acai
Agave Vanilla Cream
Black Cherry with Tarragon
Citrus Hibiscus Orange
Pineapple Cream Soda

Viva Sol
3 Gallon BIB
Jamaica
Pina
Horchata
Melon
Tamarindo

Fresh Brew Tea – 1 Sachet 3 Gallon
Lipton
32 Pack of 3oz Sachet Tea (Black)
24 Pack of 3oz Sachet Tea (Flavor)

Tea Kitchen
32 Pack of 3oz Tea Sachet (Black)

Juice Cartridge
1 Gallon
Dole 100% Orange
Dole 100% Apple Juice
Dole 20% Passion Orange Guava
Dole 15% Cranberry

Bubly
3 Gallon BIB
Lime
Raspberry
Orange

Siberian Chill
3 Gallon BIB
Very Cherry Blue
Raspberry

Fresh Non-Carbs
12 Blocks/Case
3 Concentrate Glns
Black Tea
Lemonade
Mango Refresca
Watermelon Lime Refresca
Strawberry Basil Lemonade
Green Tea w/ Citrus

DocuSign Envelope ID: 5DB6E2D2-8865-4C2F-9B02-F38647AC4CB4

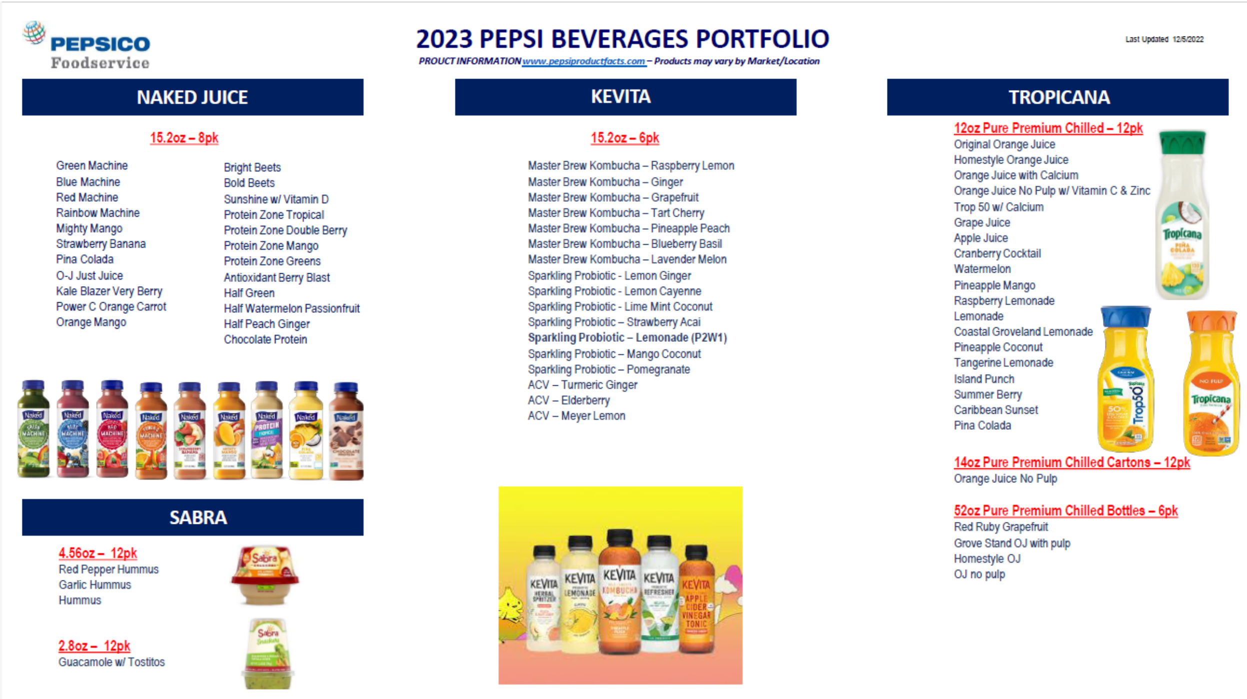

*The 2023 Pepsi Beverages Portfolio depicts Pepsi's national offerings. The Parties acknowledge and agree that some 2023 Pepsi Beverages Portfolio products may not meet the University's policies and, as such, may not be offered under this Agreement.

EXHIBIT B
ANNUAL SPONSORSHIP BENEFITS

The University will provide Pepsi the following or mutually-agreed to similar sponsorship benefits on an annual basis throughout the Term of this Agreement. The University may offer Pepsi additional sponsorship benefits on an ad-hoc basis throughout the Term. Pepsi will provide consideration for these additional benefits as mutually agreed to with the University.

1) ATHLETICS

a) Use of Marks:
 i) The rights to use Cal Athletics' trademarks and logos

b) Web Assets:
 i) 250,000 OAS ad impressions on Calbears.com
 ii) Pepsi Composite Calendar on Calbears.com

c) Social Media:
 i) (1) Four (4) social media posts on official Cal Athletics feeds:
  (a) two (2) during football season
  (b) two (2) during basketball season

d) Football:
 i) Travel with Team:
  (1) Opportunity for two PepsiCo-designated individuals to travel with football team to away game of PepsiCo's choice, provided preferred game is indicated sufficiently in advance to allow for adequate planning, preparation, etc.
   (a) For the 2023 football season: two *additional* PepsiCo-designated individuals may travel with football team to the away game versus the University of Utah on October 14, 2023
   (b) For the 2024 and 2025 football seasons: if the basketball trips noted in e)iv)(1)(b) below are not available, two *additional* PepsiCo-designated individuals may travel with football team to the away games chosen by PepsiCo as part of d)i)(1)
 ii) Presenting Sponsorship:
  (1) One (1) Presenting Sponsorship of Cal Athletics at one mutually-determined regular season home football game to include:
   (a) on-field presentation to Pepsi rep;
   (b) video board logo & public address recognitions during the game
   (c) opportunity to distribute samples and/or a free premium item to a fan at such game, with such items to be provided by Pepsi, at its cost, and subject to Cal Athletic and Learfield's prior approval.
 iii) Signage:
  (1) One (1) logo recognition with a corresponding public address announcement at each home game recognizing Pepsi as the Official Beverage Partner of Cal Athletics
  (2) One (1) permanent field sign
  (3) Two (2) minutes in-game LED takeover at each home football game
  (4) One (1) minute halftime LED takeover at each home football game
  (5) Concessions branding
 iv) Video board:
  (1) One (1) season-long videoboard promotion, "Pepsi T-Shirt Toss"
 v) Tickets & Hospitality:
  (1) Four (4) Stadium Club season tickets
  (2) Four (4) regular reserved season tickets
  (3) Three (3) season parking passes
  (4) Eight (8) season pre-game CBSP hospitality passes
 vi) Print:
  (1) Logo inclusion on at least one printed item
  (2) One (1) full-page color advertisement in game day programs
 vii) Radio:
  (1) Two (2) :30 radio spots per football broadcast
  (2) One (1) radio feature - Pre-Game Coach Wilcox Interview
  (3) Two (2) radio billboard reads per football broadcast

e) Basketball:
 i) Presenting Sponsorship:

(1) One (1) Presenting Sponsorship of Cal Athletics at one mutually-determined regular season home MBB game to include:
(a) on-court presentation to Pepsi rep;
(b) video board logo & public address recognitions during the game
(c) opportunity to distribute samples and/or a free premium item to a fan at such game, with such items to be provided by Pepsi, at its cost, and subject to Cal Athletic and Learfield's prior approval.

ii) Signage:
(1) One (1) logo recognition with a corresponding public address announcement at each home game recognizing Pepsi as the Official Beverage Partner of Cal Athletics
(2) Concessions branding
(3) Two (2) minutes of in--game LED takeover at each men's basketball home game, to include courtside signage

iii) Tickets & Hospitality:
(1) Two (2) courtside or premium seat tickets for up to five (5) mutually-determined home games each season
(2) Ten (10) men's basketball regular reserved season tickets
(3) Three (3) men's basketball season parking passes
(4) Four (4) women's basketball season tickets

iv) Travel with Team:
(1) For each of the 2024-2025 and 2025-2026 basketball seasons:
(a) *if basketball trips are available*, two PepsiCo-designated individuals to travel with basketball team to away game mutually agreed upon, based upon travel opportunities
(b) *if basketball trips are not available*, PepsiCo will have additional football travel rights, as noted in d)i)(1)(b) above

v) Radio:
(1) Two (2) :30 radio spots per basketball broadcast
(2) One (1) radio feature read, Post-Game Coach Madsen Interview
(a) Read during all 29 regular season men's basketball games
(3) Two (2) radio billboard reads per basketball broadcast

vi) Print:
(1) One (1) full-page color advertisement in game day programs

f) Baseball:
i) Signage:
(1) One (1) outfield wall sign
ii) In-Game Promotion:
(1) One (1) season long promotion at all home baseball games

g) Olympic Sports:
i) One (1) permanent sign at the following venues:
(1) Levine-Fricke Field (softball)
(2) Spieker Aquatics Center
(3) Hellman Tennis Center
(4) Edwards Stadium (soccer)
ii) One (1) Olympic Sports Pass

2) CAL ALUMNI ASSOCIATION

a) Digital + Event Sponsorship:
i) Four (4) Facebook posts highlighting Pepsi's partnership with the university
ii) Year-long run of site web ads on alumni.berkeley.edu
iii) Exclusive non-alcoholic beverage sponsor for Homecoming for the entire length of contract. Includes logo inclusion in all marketing material for the event and tabling / product distribution.

3) CAL PERFORMANCES

a) Sponsorship Recognition:
i) Logo placement on Institutional Partners slide on plasma screens throughout Zellerbach Hall
ii) Log placement on "Corporate Giving" webpage
iii) Listing on Annual Support page in all program books throughout the season
iv) Listing on Supporter Wall in Zellerbach Hall lobby

b) Hospitality:
   i) Ten (10) complimentary tickets to season performances
   ii) Six (6) complimentary, one-time parking passes

4) CAREER CENTER

a) Blue Level Sponsorship:
   i) Branding & Visibility:
      (1) Employer logo with link on Career Center website
      (2) Employer added to exclusive Handshake Collection to highlight jobs to students
      (3) Year-round visibility on digital displays in the Career Center
   ii) Student Access & Outreach:
      (1) Handshake Job Postings and Events promoted on Career Center website
      (2) Company logo highlighted on social media each semester
      (3) Two (2) targeted student emails per semester
      (4) Priority Invitation for special event participation
      (5) Career Center room use and Handshake scheduling for coffee chats, includes parking pass
      (6) One (1) complimentary resume book download
   iii) Career Fairs & Events:
      (1) Guaranteed access to career fairs, if registered by deadline
      (2) One (1) complimentary resume book from career fairs Pepsi attends
   iv) Consultation:
      (1) Exclusive partner email for concierge service
      (2) Ongoing support from dedicated Career Center staff
      (3) Invitation to Annual Berkeley Circle Roundtable luncheon
      (4) Strategic recruitment meeting with Career Center Employer Relations staff
      (5) DEI recruiting consultation with Career Center Employer Relations staff

5) ESPORTS

a) Cal Esports Tabling Events:
   i) Opportunity to table at five (5) different key events throughout the semester, including the Cal Esports Open House, Halloween LAN and more
b) Cal Esports Jerseys/Merchandise:
   i) Mutually agreed-to premier logo placement on several key areas on jersey/apparel
c) Branding within Cal Esports Center:
   i) Banner with one (1) mutually-agreed-to logo within the center,
d) Monthly Email Newsletter Inclusion (10,000+ recipients):
   i) Content to be mutually agreed upon

6) NEW STUDENT SERVICES

a) Digital marketing:
   i) Guidebook Mobile App Ad:
      (1) clickable banner ad published during high usage times that will direct the user to in-app content to a URL through a web browser
b) Student Resource Fair:
   i) Tabling opportunity at the Student Resource Fair during Golden Bear Orientation in August

7) **PARKING & TRANSPORTATION**

a) Sponsorship of Bike-to-Work Day event hosted by Parking & Transportation (P&T)
   i) **Digital + In-person**
      (1) One (1) mention of sponsorship in P&T employee email promoting event
      (2) Opportunity to table at energizer station:
         (a) provide drink or snack products for attendees (waters, fresh fruits, trail mix or other healthy snack)

8) **PEOPLE & CULTURE**

a) Diamond sponsor of The NOW Employee Conference:
   i) Full-color clickable logo on the People & Culture (P&C) NOW Conference website
   ii) Mention on P&C social media accounts preceding the event

iii) Sponsorship logo recognition on archived materials in P&C virtual conference resource center
iv) Sponsorship of one breakout session (includes brief sponsor mention by a conference facilitator)
v) Promotional mention in P&C newsletter preceding the event
vi) Two (2) tickets to attend the NOW Conference, includes full access to the conference program
vii) Public recognition/mention during the conference opening and closing remarks
viii) IMPORTANT:
(1) NOTE: NOW Conference rotates annually between in-person and virtual
(2) NOTE: NOW Conferences may be held at an external facility that may have catering contracts/relationships with a competing company

b) Staff Appreciation Week (SAW)
i) Sponsorship of one (1) in-person or virtual SAW event annually:
(1) One (1) mention of sponsorship in P&C employee email promoting SAW
(2) One (1) sponsorship mention on SAW website promoting sponsored event

9) **RECREATION & WELLBEING**
a) Caltopia, presenting sponsor:
i) Presenting sponsor designation/placement on all Caltopia printed and digital materials:
(1) Logo Listing on Welcome Banner
(2) Thank you sponsors backdrop
(3) Event Guide
(4) Logo featured on Official Caltopia T-shirts
(5) Up to eight (8) 8'x10' booth spaces spread throughout the exhibition area
(6) Up to 6 event guide ads in the official Caltopia Event Guide (printed and digital)
ii) Wellness Happens Here sponsorship:
(1) Presenting sponsor designation/placement on all Wellness Happens Here printed and digital materials
(2) Opportunity to table at the Rec Center
(3) Opportunity to sponsor select group exercise classes
(4) Opportunity to create customized programming for event
iii) Customized Event Tabling:
(1) Opportunity to table at any Recreation & Wellbeing locations based on availability
(a) All programs and events to be mutually agreed upon
iv) Signage in fitness areas:
(1) Up to six (6) logo inclusions on murals throughout the Recreational Sports Facility
(a) To be mutually agreed upon
v) Digital marketing on websites and social media:
(1) Opportunity to include ads in monthly Recreation & Wellbeing Newsletter
(2) Ongoing "Thank you to our sponsors" recognition on rotating footer of Recreation & Wellbeing website
(3) Opportunity to advertise and collaborate on mutually agreed upon social media campaigns on Recreation & Wellbeing social channels (Twitter, Facebook & Instagram)
(a) Limited to two (2) posts per month per channel

10) **STUDENT UNION**
a) Signage:
i) Digital screens - year-round opportunity (24/7)
ii) Physical signage (A-frames) - in conjunction with a Student Union event
b) Digital Presence:
i) Website listing/logo: year-long sponsorship recognition on the SU website
ii) Social Media: four (4) Instagram postings/year; two (2) per semester
iii) Student Union newsletter inclusion – one (1) per semester
c) Market Days: six (6) tabling opportunities per year
d) Open House/Cal Day: opportunity to participate in one (1) event and handout product
e) Single day marketing event at the Student Union: opportunity to engage with students, staff and faculty
f) Dorm Dash Event: opportunity to participate in a freshmen event in August.

11) **SUTARDJA CENTER FOR ENTREPRENEURSHIP AND TECHNOLOGY (SCET)**

a) Sponsorship of the SCET Fellow Program:
   i) The SCET Fellow Program takes place each fall semester, partnering with numerous campus entities (e.g. student groups, academic departments, etc.) and hosts a variety of technical challenges.
   ii) Fall 2023 challenges include: AR Marketing, Energy, NASA Space Apps.
      (1) Invitation for Pepsi representative to engage on SCET Fellows leadership committee to develop challenge
      (2) Invitation for Pepsi representative to serve as mentor and judge for challenges
      (3) Invitation for Pepsi representative to attend SCET Fellow Program end-of-term events, including presenting the Fellow Program winner at the SCET Collider Cup.
      (4) SCET will acknowledge PepsiCo support as appropriate on:
         (a) SCET website
         (b) SCET fellow program materials (web and print)
         (c) SCET Newsletter
         (d) SCET Social Media posts related to the fellow program

12) **VISITOR SERVICES**
a) Cal Day Gold Sponsor:
   i) One (1) premium 10x10 sponsor booth/activity space in prime location; includes one (1) six foot table
   ii) One (1) brand name inclusion in pre-Cal Day social media posts (official #CalDaySponsor)
   iii) Banners of variable size thanking sponsor displayed during event (A-Frame signage, Cable Car banners, etc.)
   iv) One (1) rotating banner ad in Official Cal Day Guidebook app

EXHIBIT C
UNIVERSITY OF CALIFORNIA, BERKELEY
TRADEMARK GUIDELINES AND REQUIREMENTS

These provisions apply to all use of the names, logos, indicia, landmarks, slogans, graphic images, and other trademarks belonging to The Regents of the University of California pursuant to the terms of the Agreement, including, specifically on products for resale and/or distribution, in all forms of media, such as electronic and/or print advertising, promotion, social media and/or on websites including their inclusion in Internet domain names. For the avoidance of any doubt, while this Exhibit is intended to provide Pepsi with general guidelines, any and all uses of the University Marks is subject to University's prior, written approval and such approval is confirmation that such guidelines have been met.

a) All variations of the University names are trademarks of the University, and property of The Regents of the University of California ("UC Regents").

b) Acceptable nominations are "UC Berkeley," "University of California Berkeley," "Berkeley," "Cal," or "California."

c) "University of California" may not be used without specifically indicating the Berkeley Campus. Examples of correct uses include: "University of California Golden Bears" or "University of California, Berkeley."

d) The University Marks should always present a positive image.

e) In accordance with the State of California Education Code Section 92000, the University's name and/or the Marks may not be used in any way that could state or imply an endorsement of a business, social, political, religious or economic movement, activity, program or group, or in advertising services. Vendors/Entities may be designated as "Sponsor," "Preferred Provider" or other term agreeable to the University, including the Approved Designations set forth in Exhibit A, which suggests that the entity is a supporter of the University's mission of teaching, research, and public service.

f) The University Seal is restricted from use on items which could be construed as University documents, publications, or advertisement except when such documents or publications are used by the University for official University business such as checks, certificates, or rubber stamps. The University Seal may not be defaced, altered, overprinted, or dismantled in any manner.

g) University Marks are not to be associated, or used on or in connection with alcohol, tobacco, condoms, gambling products, items which could be used to maim or kill, or that could present a high liability exposure. For example, University Marks should not be used in conjunction with knives, guns, or cigarette lighters.

h) The University's Marks will not be licensed for use in association with certain other mark(s), words or phrases, for example: the Playboy bunny, Coed Naked, profanity, ethnic/gender/religious slurs, pro-hazing designs or designs that are deemed to be in poor taste. Further, art that is in some way degrading or demeaning, or reflects poorly on the University image, will not be approved.

i) Copyright Requirements: Any artwork or creative material created by the University will be considered University-owned designs and the UC Regents retain all rights in and to these designs. Copyright ownership must be attributed to the UC Regents as follows:

© 20XX UC Regents

© 20XX The Regents of the University of California

j) In accordance with the Federal Trademark Dilution Act of the United States Patent and Trademarks Office, modifying or altering the University's trademarks is prohibited. University Marks should not be obscured or changed in such a way which would dilute the integrity of the design or its brand recognition.

k) All marketing collateral, advertisements, product packaging, web pages, and any other content that includes the University Marks should also include the proper trademark attribution statement crediting ownership of the University Marks to the UC Regents. Typically, the attribution statement is displayed at the end of the material, in the footer of the document, or on the back of the package. The correct trademark attribution statement is:

> The [XXX] marks and related logos are trademarks associated with the University of California, Berkeley campus and owned by and used with permission of The Regents of the University of California.

All designs, which include UC Berkeley's name and/or other trademarks, and modifications of previously approved designs must be submitted to a delegated authority of the University, for review and approval before such designs are produced, reproduced or sold. Such delegated authority may seek input from other campus units/leaders for socially and/or politically sensitive uses of the University's Marks before granting approval.

l) The use of the University's Marks, when used in association with a product or service, is permitted solely in accordance with written permission from a delegated authority of the University or an authorized representative. The agreement may be in the form of a trademark license agreement or other agreement with appropriate trademark provisions.

EXHIBIT D
UNIVERSITY MARKS

Unless otherwise agreed by the Parties in writing, the following "Primary Marks" as identified below may be used as specifically approved by University in the manner set forth in this Agreement:

Primary Marks

Athletics Cal Script

Cal Alumni Association

Cal Performances

Career Center

Esports

New Student Services – Golden Bear Orientation

Parking & Transportation

People & Culture

Berkeley Recreation and Wellbeing.

Student Union

Sutardja Center for Entrepreneurship and Technology (SCET)

University Partnership Program

DocuSign Envelope ID: 5DB6E2D2-8865-4C2F-9B02-F38647AC4CB4

Secondary Marks
[May only be used in association with one of the aforementioned Primary Marks or with an approved Co-Branded Mark as defined below]

Berkeley/ UC Berkeley

Co-Branded Marks
Pepsi may develop and use custom joint (aka co-branded) artwork (i.e., co-branded logo) (collectively "**Co-Branded Marks**") that include the Primary Marks as described in this Exhibit D, subject to the Trademark Use and Co-brand Guidelines of Exhibit C, or any subsequent or similar Brand Guidelines provided by the Office of Communication and Public Affairs [e.g., Brand Partnership System: https://upp.berkeley.edu/business-partners/build-your-brand]. Development and use of Co-Branded Marks shall be subject to the terms of the Agreement, including its review provisions. Pepsi shall have the right to use the Co-Branded Marks for commercial and marketing programs, in all national markets, subject to the provisions of the Agreement.

The Parties agree to exchange an updated list of University Marks and Co-Branded Marks each Year of the Agreement, either on the anniversary of the Effective Date or in connection with the annual payment.

EXHIBIT E
CONSIDERATION

Subject to the express conditions set forth herein, during the Term of this Agreement, Pepsi agrees to provide University with the monetary and non-monetary consideration described below.

(A) **Beverage Partnership Funding**

Each Year throughout the Term, Pepsi agrees to provide University with annual Beverage Partnership Funding. The Beverage Partnership Funding is earned and paid relative to University volume performance, as set forth below in connection with an index rate (i.e. the Annual Units Threshold) or growth rate to certain volume assumptions, pursuant to the following:

Contract Year	Baseline Beverage Partnership Funding	minimum required Annual Units Threshold	95% of minimum required Annual Units Threshold	105% of minimum required Annual Units Threshold	Payment 1 Due*	Payment 2 Due**
1	$570,000	66,515	59,863	69,841	August 1, 2023 or within thirty (30) days of execution	September 1, 2024
2	$570,000	66,515	59,863	69,841	August 1, 2024	September 1, 2025
3	$587,100	66,515	59,863	69,841	August 1, 2025	September 1, 2026
4	$604,713	66,515	59,863	69,841	August 1, 2026	September 1, 2027
5	$622,854	66,515	59,863	69,841	August 1, 2027	September 1, 2028
6	$641,540	66,515	59,863	69,841	August 1, 2028	September 1, 2029
7	$660,786	66,515	59,863	69,841	August 1, 2029	September 1, 2030
8	$680,610	66,515	59,863	69,841	August 1, 2030	September 1, 2031
9	$701,028	66,515	59,863	69,841	August 1, 2031	September 1, 2032
10	$722,059	66,515	59,863	69,841	August 1, 2032	September 1, 2033

*Each Beverage Partnership Funding payment will be made in two (2) installments. The first installment will advance 90% of the base Beverage Partnership Funding for the applicable Year and is due as Payment 1 for the applicable Year in accordance with the chart set forth above.

**The second installment will pay 10% of the baseline Beverage Partnership Funding for the applicable Year in arrears and is due as Payment 2 date for the applicable Year in accordance with the chart set forth above.

University acknowledges and agrees that each Year the Beverage Partnership Funding may be adjusted based on the number of Units purchased from Pepsi and sold throughout the Participating Food Service Locations and the Facilities during the applicable Year, as compared to the minimum required Annual

Units Threshold as set forth above. The adjustment (if any) will occur via the Payment 2 of Beverage Partnership Funding that Year, according to the examples below:

- For each percent of decline below 59,863 Units in a given Year, that Year's Beverage Partnership Funding will be reduced by the percent of the decline;
- For each percent of increase above 69,841 Units in a given Year, the University will receive a rebate based on the percent increase multiplied by the Beverage Partnership Funding for that year.

For example:

- If the University's volume of Units purchased in Year 2 were 53,876 Units, a 10% decline from 59,863 Units, then that Year's Beverage Partnership Funding would be reduced by 10%, to $513,000;
- If the University's volume of Units purchased in Year 2 were 76,825 Units, a 10% increase from 69,841 Units, then that Year's Beverage Partnership Funding would be increased by 10%, to $627,000.

A portion of the Beverage Partnership Funding will be paid directly to the Cal Alumni Association, as determined by University each Year.

(B) **Other Monetary Consideration**

In addition to the Beverage Partnership Funding noted above, Pepsi will provide the University the following consideration within sixty (60) days of August 1 in Years 1-10, as defined above, of the Term of the Agreement.

(1) **Sustainability**. In Years 1-10 of the Term, Pepsi agrees to provide University with funding in the amount of thirty thousand US dollars ($30,000) to be used by University in furtherance of University's sustainability endeavors. These funds must be used to address University priorities related to sustainability in a materially impactful way; the use and communications related to these funds will be mutually agreed to by Pepsi and the University. Upon request by Pepsi, the University will summarize the specific expenditures related to these funds. These funds are not a reimbursement and are not subject to any volume thresholds; they are annual, guaranteed payments in support of the University's sustainability efforts.

(2) **Marketing**. In Years 1-10 of the Term, Pepsi agrees to provide University with funding in the amount of one hundred and twenty thousand US dollars ($120,000) in support of marketing activities related to this Agreement, including, but not limited to, those outlined in Exhibit B of this Agreement. Each Year Pepsi will provide one hundred thousand US dollars ($100,000) of these funds upfront to the University. The remaining twenty thousand US dollars ($20,000) will be held by Pepsi until provided to the University for mutually agreed upon marketing activities in the given Year. These funds are not subject to any volume-related adjustments. Upon request by Pepsi, the University will summarize how these funds have been spent, though not more frequently than once per quarter. Amounts remaining unspent at the end of each Year will not rollover to the following Year and will expire at the end of the Term and are retained by Pepsi.

(3) **Flex Fund**. In Years 1-10 of the Term, Pepsi agrees to provide University with fifteen thousand US dollars ($15,000) to be used for mutually agreed upon programming to enhance existing programs or new opportunities in a given Year. Upon request by Pepsi, the University will summarize the specific expenditures related to these funds. These funds are not subject to any volume-related adjustments. Amounts remaining unspent at the end of each Year will not rollover to the following Year and will expire at the end of the Term and are retained by Pepsi.

(4) **Commissions**. Pepsi agrees to provide University with commissions, as a percentage of the actual cash ("**cash in bag**" or "**CIB**") collected by Pepsi from the Vending Machines placed at the Facilities, less any applicable government-imposed taxes/fees and deposits, as applicable ("**Commissions**"). Such Commissions shall be at the rate of 30% and shall be calculated as follows:

(CIB*Commission Rate) – applicable CRV = Commission due

i. Commissions Payment. Pepsi agrees to pay Commissions to University within thirty (30) days of the end of each four (4) week accounting period established by Pepsi. Pepsi shall make all pertinent revenue and sales records respecting the Vending Machines available to University. University agrees that it is responsible for reviewing such records and that any claim or dispute relating to the Commissions must be brought by University in writing within one (1) year of the date such Commissions payment is due.

ii. Change to Commission Rate/Formula. University agrees that Pepsi shall have the right to change the Commission Rate and/or its formula/method for calculating Commissions as may be required by applicable laws or as reasonably necessary to respond to legislative acts in order that the Commission Rate remains cost neutral.

iii. Vend Price. The initial vend prices for University to qualify for any Commissions are set forth in the Commission chart above. Pepsi shall have the right to increase vend prices by $0.25 in Years 2, 5, and 8 of the Term. University acknowledges that Pepsi has the right to pass through any incremental fees, deposits, taxes or other governmentally-imposed charges (whether local, state, federal, or judicially-imposed on manufacturers, distributors, consumers or otherwise). The pass-through of any such governmentally-imposed feeds, deposits, taxes or charges on the Products will be in addition to any scheduled Vend Prices increases set forth herein or notification restrictions that may be specific in this Agreement.

(C) **Payments**

(1) Unless otherwise indicated by the University, all amounts payable will be paid in United States dollars by ACH electronic payment and submitted via instructions provided by the University. Payments will be addressed to "UC Regents," and will be due within sixty (60) days of the payment dates indicated in this Agreement, unless otherwise mutually agreed to by the University and Pepsi.

i. Payments due to the Cal Alumni Association will be paid in United States dollars by ACH electronic payment and submitted via instructions provided by the University and/or the Cal Alumni Association, and will be addressed to "Cal Alumni Association." Payments will be due within sixty (60) days of the payment dates indicated in this Agreement, unless otherwise mutually agreed to by the University and Pepsi.

(2) **Right of Offset**. Pepsi reserves the right to withhold payments due hereunder as an offset against amounts not paid by University or its Food Service Providers for Products ordered from and delivered by Pepsi, with regard to overdue balances owed by University's student service provider for vending Product purchased with student meal cards, and any and all balances due and payable to Pepsi pursuant to this Agreement or any separate services agreement between University and Pepsi and/or its subsidiaries and affiliates.

(3) **Tax**. Neither Pepsi nor its affiliates will be responsible for any taxes payable, fees or other tax liability incurred by University in connection with the consideration or any other fees payable by Pepsi under this Agreement. If Pepsi is charged common area maintenance fees, taxes or other charges related to Pepsi's occupation of the space allocated to its Equipment at the Facilities, Pepsi may make an adjustment to the consideration provided in Section 4.(I)(1) below to offset such costs.

(D) **Royalties**

(1) In conjunction with Pepsi's sale and/or non-sale distribution of products bearing the University and Pepsi names, logos, and other trademarks (Co-Branded Marks), Pepsi will pay Campus' Licensing (agency) royalties based on the prevailing standard royalty rate. Licensing is renewed annually and the sale or distribution of products bearing Co-Branded Marks is contingent on Pepsi's maintaining a license separate from any sponsorship agreement and/or Pepsi using a vendor with an executed license agreement with the University.

(E) **Non-Monetary Consideration**

(1) **Activation Support**. In Years 1 and 6 of the Term, Pepsi agrees to accrued on behalf of and pay the University with activation support with a value of up to fifty thousand US dollars ($50,000), which will be used and spent by Pepsi to pay for mutually agreed upon co-branding items, menu boards, signage, digital media, and similar equipment, material, and activities. Pepsi will reimburse the University for any internal costs incurred. In Years 2, 3, 4, 7, 8, 9, and 10 of the Term, Pepsi agrees to provide University

with such activation support up to a value of fifteen thousand US dollars ($15,000). The annual budgets for this activation support will be reviewed on a quarterly basis throughout the Term. University acknowledges and agrees that unused activation support will not be carried over to a subsequent Year and will not be redeemable for cash payment; for the avoidance of doubt, any unused Activation Support will expire at the end of each Year and Term and will be retained by Pepsi

(2) **Product Free of Charge**. Upon request from University, Pepsi agrees to provide up to 2,400 cases annually of 12oz carbonated soft drink cans (including bubly, and/or 16.9oz plastic Aquafina product pursuant to the University's discretion) in Complimentary Product each Year, provided, however, that University will administer all requests through a central contact so that University may prioritize the requests. University acknowledges and agrees that unrequested Product in any Year shall not be carried over to the subsequent Year or be redeemable for cash payment.

(F) **Additional Consideration**

(1) **Emergency Water**. Pepsi agrees to provide University with 1,296 cases of 16.9oz Aquafina for University's emergency water supply in Year 1 of the Agreement. This emergency water supply will be rotated out and replaced by new product on an annual basis. For the avoidance of doubt: i) Emergency Water will not be construed as non-monetary Consideration and Pepsi will charge University for the cost of all Emergency Water used; and ii) Emergency Water will consist exclusively of plastic water bottles and will not be subject to non-plastic restrictions if any.

(An overview of the monetary and non-monetary consideration of this Agreement follows below)

OVERVIEW OF MONETARY AND NON-MONETARY CONSIDERATION

This table is an overview of the baseline monetary and non-monetary consideration Pepsi is to provide the University under this Agreement, subject to the details outlined in sections (A), (B), (E), and (F) of this Exhibit E

Year	1	2	3	4	5	6	7	8	9	10
Beverage Partnership Funding	$570,000	$570,000	$587,100	$604,713	$622,854	$641,540	$660,786	$680,610	$701,028	$722,059
Full Service Vending Commissions*	$15,400	$16,800	$16,800	$16,800	$18,200	$18,200	$18,200	$19,600	$19,600	$19,600
Marketing Support	$120,000	$120,000	$120,000	$120,000	$120,000	$120,000	$120,000	$120,000	$120,000	$120,000
Activation Support	$50,000	$15,000	$15,000	$15,000	$15,000	$50,000	$15,000	$15,000	$15,000	$15,000
Product Free of Charge	2,400 cases	2,400 cases	2,400 cases	2,400 cases	2,400 cases	2,400 cases	2,400 cases	2,400 cases	2,400 cases	2,400 cases
Sustainability Support	$30,000	$30,000	$30,000	$30,000	$30,000	$30,000	$30,000	$30,000	$30,000	$30,000
Flex/Tech Fund	$15,000	$15,000	$15,000	$15,000	$15,000	$15,000	$15,000	$15,000	$15,000	$15,000
Emergency Water	1,296 cases	1,296 cases	1,296 cases	1,296 cases	1,296 cases	1,296 cases	1,296 cases	1,296 cases	1,296 cases	1,296 cases

*Anticipated Commissions as of Effective Date of Agreement.

UC Berkeley 2023 Contract Pricing

Item<br>*Bottle & Can*	Size	Packaging	Unit Price	Package Price
Aquafina	16oz	24	$1.31	$31.50
Aquafina	16.9oz	24	$0.41	$9.73
Aquafina	20oz	24	$0.79	$18.96
Aquafina	1 Liter	15	$1.29	$19.28
Aquafina	1.5 Liter	12	$1.51	$18.06
Bubly	12oz	24	$0.60	$14.30
Bubly	16oz	12	$0.94	$11.22
Bundaberg	375ml	24	$1.38	$33.06
Carbonated Soft Drinks	7.5oz	24	$0.61	$14.62
Carbonated Soft Drinks (Glass)	12oz	24	$1.12	$26.85
Carbonated Soft Drinks	12oz	24	$0.63	$15.00
Carbonated Soft Drinks (Nitro)	13.65oz	12	$1.68	$20.20
Carbonated Soft Drinks	16oz	12	$1.47	$17.60
Carbonated Soft Drinks	20oz	24	$1.00	$23.92
Dole	15.2oz	12	$1.91	$22.91
Evolve	11.16oz	12	$3.48	$41.72
Gatorade	20oz	24	$1.40	$33.50
Gatorade G Fit	28oz	15	$2.15	$32.21
Gatorade Zero Sugar	28oz	15	$2.72	$40.74
Gatorade Zero Sugar Protein	16.9oz	12	$2.00	$24.02
Gatoryte	20oz	12	$2.00	$24.02
Gatorade Propel	20oz	12	$1.52	$18.24
Kevita	15.2oz	12	$1.38	$16.51
LifeWTR	700ml	12	$1.72	$20.66
LifeWTR	20oz	24	$1.22	$29.18
LifeWTR	1L	12	$1.78	$21.31
LifeWTR	1.5L	12	$2.07	$24.79
Lipton Pure Leaf	18.5oz	12	$1.84	$22.05
Muscle Milk	14oz	12	$4.26	$51.15
Naked Juice	16.9oz	12	$2.86	$34.28
Naked Juice Protein	15.2oz	8	$2.73	$21.82
Naked Juice Smoothie	15.2oz	8	$2.45	$19.57
Naked Juice	64oz	6	$7.63	$45.75
Ocean Spray	15.2oz	12	$1.91	$22.91
Proud Source	12oz	24	$1.04	$25.00
Proud Source	16oz	24	$1.31	$31.50
Proud Source	750ml	12	$2.33	$28.00
Starbucks Double Shot	6.5oz	12	$2.49	$29.93
Starbucks Nitro	9.6oz	12	$2.76	$33.06
Starbucks Cold & Crafted	11oz	12	$2.40	$28.75
Starbucks Cold Brew	11oz	12	$3.32	$39.89

Starbucks Frappuccino	9.5oz	15	$2.18	$32.73
Starbucks Frappuccino	13.7oz	12	$2.49	$29.93
Stubborn	12oz	18	$1.00	$18.00
Tropicana	12oz	12	$1.34	$16.09
Tropicana	14oz	12	$1.83	$21.91
Tropicana	15.2oz	12	$1.91	$22.91
Tropicana	52oz	6	$5.66	$33.93

Item<br>*Fountain*	Size	Packaging	Unit Price	Package Price
PepsiCo Corp. Carbonated Soft Drinks	1 gallon BIB	5	$19.90	$99.50
PepsiCo Corp. Carbonated Soft Drinks	1 gallon BIB	3	$20.58	$61.74
Crush	1 gallon BIB	5	$19.90	$99.50
Dr. Pepper	1 gallon BIB	5	$19.90	$99.50
Bubly	1 gallon BIB	3	$20.58	$61.74
Dole Juice	1 gallon BIB	3	$33.50	$100.50
Dole Juice	1 gallon BIB	1	$33.50	$33.50
Flavor Shots	1 gallon BIB	1	$26.40	$26.40
Gatorade	1 gallon BIB	3	$21.09	$63.27
Lipton Brisk	1 gallon BIB	5	$19.90	$99.50
Lipton LCT Iced Tea	1 gallon BIB	3	$20.58	$61.74
SoBe LifeWater	1 gallon BIB	3	$21.23	$63.69
Stubborn Soda	1 gallon BIB	3	$21.00	$63.00
Tropicana Lemonade	1 gallon BIB	3	$19.90	$59.70
Viva Sol	1 gallon BIB	3	$19.90	$59.70
SodaStream Flavors	1 gallon BIB	1	$50.00	$50.00

Item<br>*Supply Items*	Size	Packaging	Unit Price	Package Price
Double Poly Cups	16oz	1200	$0.08	$97.22
Double Poly Cups	22oz	1200	$0.08	$97.22
Double Poly Cups	32oz	480	$0.17	$82.27
Lids for Double Poly Cups	16/22oz	1000	$0.05	$54.14
Lids for Double Poly Cups	32oz	960	$0.06	$59.36
Compostable Cups	16oz & 22oz	1000	$0.12	$116.68
Compostable Cups	32oz	500	$0.23	$115.00
CO2	20#	1	$32.15	$32.15
CO2	50#	1	$54.61	$54.61

Annual price increases will follow contracted increase amounts per agreement

EXHIBIT F

FIRST AMENDMENT TO APPENDIX – DATA SECURITY AND PRIVACY

SAFEGUARD STANDARD FOR PAYMENT CARD DATA

A. Supplier agrees that it is responsible for the security of Cardholder Data (as currently defined by the Payment Card Industry Data Security Standard and Payment Application Standard Glossary of Terms, Abbreviations, and Acronyms) that it possesses (if any), including the functions relating to storing, processing and transmitting Cardholder Data. In this regard, Supplier represents and warrants that it will implement and maintain certification of Payment Card Industry ("PCI") compliance standards regarding data security, and that it will undergo independent third party quarterly system scans that audit for all known methods hackers use to access private information and vulnerabilities that would allow malicious software (*e.g.*, viruses and worms) to gain access to or disrupt UC Information Resources. These requirements, which are incorporated herein, can be found at https://www.pcisecuritystandards.org/security_standards/index.php. Supplier agrees to provide at least annually, and from time to time at the written request of UC, current evidence (in form and substance reasonably satisfactory to UC) of compliance with these data security standards, which has been properly certified by an authority recognized by the payment card industry for that purpose.

B. In connection with credit card transactions processed for UC, Supplier will provide reasonable care and efforts to detect fraudulent payment card activity. In performing the Services, Supplier will comply with all applicable rules and requirements, including security rules and requirements, of UC's financial institutions, including its acquiring bank, the major payment card associations and payment card companies. If during the term of an Agreement with UC, Supplier undergoes, or has reason to believe that it will undergo, an adverse change in its certification or compliance status with the PCI standards and/or other material payment card industry standards, it will promptly notify UC of such circumstances.

C. Supplier further represents and warrants that software applications it provides for the purpose of performing Services related to processing payments, particularly credit card payments, are developed in accordance with all applicable PCI standards, and are in compliance with all applicable PCI standards, including but not limited to The PCI SSC's Software Security Framework (SSF), Point to Point Encryption Solution Requirements (P2PE) including approved card readers or Point of Interaction (POI). As verification of this, Supplier agrees to provide at least annually, and from time to time upon written request of UC, current evidence (in form and substance reasonably satisfactory to UC) that any such application it provides is certified as complying with these standards and agrees to continue to maintain that certification as may be required.

D. Supplier will immediately notify UC if it learns that it is no longer PCI compliant under one of the standards identified above, or if any software applications or encryption solutions are no longer PCI compliant.

EXHIBIT G
PEPSI MARKS

PEPSI
ZERO SUGAR

bubly
sparkling water

EXHIBIT H
SUSTAINABILITY

Annual Sustainability Funding

Pepsi will provide University funding, as described in Exhibit E, to further University efforts to improve sustainability-related outcomes on Campus.

Reporting

Pepsi will provide reporting, as outlined in Exhibit J, that allows the University to assess the performance of Products at the Participating Food Service Locations and the broader Facilities, and will make commercially reasonable efforts to provide supplemental sustainability-related data available to the University upon the University's request, provided such requests are made by a single point of contact on behalf of the University.

Communications and Promotional Activities

Pepsi and the University will mutually agree on any communications, promotions, and similar activities related to this agreement, including advertising and onsite activations. For the avoidance of doubt, Pepsi and the University acknowledge and agree that these activities and materials will focus on non-single use plastic packaged products, as defined below under "Transition Away from Single-Use Plastics," with the exception of the Gatorade and Naked Juice product lines..

Pepsi and the University further acknowledge and agree that, where commercially reasonable to do so, the communications and other activities outlined in this Agreement will prioritize the promotion of those products certified as sustainable according to one of the certifications listed in section H of the University of California Sustainable Practices Policy, included as Appendix A of this Agreement

Adherence to Applicable University Policies

Pepsi will further support the University's sustainability goals by providing the University with a Product mix that allows the University and the Participating Food Service listed in Exhibit K, as well as vending machines and other "autonomous" distribution and dispensing mechanisms throughout the Facilities, to meet the waste, spend, energy, and related goals and requirements of the University of California Sustainable Practices Policy.

Pepsi acknowledges and agrees that as part of this Agreement it commits to biannual reviews of the conformity of the Product mix it makes available to the University, and the communications, promotional, stocking, and merchandising activities related to this Product mix, to:

- the University of California Sustainable Practices Policy.

Pepsi further acknowledges and agrees that, in response to these reviews, it will take verifiable measures to address any discrepancies between its activities and updated versions of the University of California Sustainable Practices Policy.

For the avoidance of doubt, in addition to the single-use plastic-related stipulations of this Agreement, further outlined below, Pepsi agrees that it will provide:

DocuSign Envelope ID: 5DB6E2D2-8865-4C2F-9B02-F38647AC4CB4

- ENERGY-STAR-designated equipment across all applicable product categories.
  - o For appliance and other equipment purchases where ENERGY STAR certification is not available, purchases should be Federal Energy Management Program (FEMP)-designated, if available.

Pepsi further acknowledges and agrees that:

- It will not use packaging foam when supplying the University with Products, Equipment, or other related materials, services, etc. as part of this Agreement.
- It will comply with the Toxics in Packaging Prevention Act (AB 455) as to be free of any intentionally introduced lead, cadmium, mercury or hexavalent chromium, and containing no incidental concentrations of these regulated metals greater than 100 parts per million (ppm) by weight.
- All packaging that it provides as part of this Agreement will meet at least one of the criteria listed below:
  - o Uses bulk packaging;
  - o Uses reusable packaging;
  - o Uses innovative packaging that reduces the weight of packaging, reduces packaging waste, or utilizes packaging that is a component of the product;
  - o Maximizes recycled content and/or meets or exceeds the minimum post-consumer content level for packaging in the U.S. Environmental Protection Agency Comprehensive Procurement Guidelines;
  - o Uses locally recyclable or certified compostable material.

Transition Away from Single-Use Plastics

Pepsi acknowledges and agrees that after **January 1, 2024**, Pepsi will not sell or otherwise make available any Products to the University that are packaged in single-use plastic, as defined by the University, with the exception of Gatorade and Naked Juice product lines, and if applicable, the Emergency Water supply described in Exhibit E. The University defines single-use plastic as:

- A product or item of packaging that is conventionally disposed of after a single use (including plastic that is recycled) and is not intended, and is not sufficiently durable, washable, or otherwise treatable, to allow for multiple refills of the original product or other products. For the avoidance of doubt, rPET and any other plastic made from recycled materials is considered a single-use plastic by the University.

With respect to Gatorade and Naked Juice product lines, Pepsi will make commercially reasonable efforts to offer non-single use plastic versions of these products to the University as soon as possible, and will provide University updates on the status of the availability of these product lines in non-single use plastic formats.

Pepsi acknowledges and agrees that after **January 1, 2030**, it will be prohibited from selling or otherwise making available any Products and any other items (giveaways, etc.) that include single-use plastic in their composition or packaging (for example, shrink-wrapped items). This prohibition also applies to any single-use plastics used in the distribution of Products and any other items at the Facilities and the Participating Food Service Locations.

Pepsi acknowledges and agrees that the cups and lids it provides the University must be fiber-based, free of intentionally added polyfluorinated chemicals (PFAS-PFOS) and BPI or CMA-certified as

DocuSign Envelope ID: 5DB6E2D2-8865-4C2F-9B02-F38647AC4CB4

biodegradable and compostable. Any Ancillary Products that feature eco-friendly messaging must be natural fiber-based items, or fiber-based items lined with a BPI or CMA-certified PLA.

EXHIBIT I
HEALTH & WELLNESS

Reporting

Pepsi will provide reporting, as outlined in Exhibit J, that allows the University to assess the performance of Products at the Participating Food Service Locations and the broader Facilities, and will make commercially reasonable efforts to provide supplemental health and wellness-related data available to the University upon the University's request, provided such requests are made by a single point of contact on behalf of the University.

Communications and Promotional Activities

Pepsi and the University will mutually agree on any signage, communications, promotions, and similar activities related to this agreement, including advertising and onsite activations. For the avoidance of doubt, Pepsi and the University acknowledge and agree that these activities and materials will:

- focus on Products considered to be "Healthier" by the UC Berkeley Food and Beverage Choices Policy (included in this Agreement as Appendix B);

Pepsi and the University further acknowledge and agree that, where commercially reasonable to do so, the communications and other activities outlined in this Agreement will prioritize, among the aforementioned "Healthier" Products, those that are free of:

- artificial colors, sweeteners, flavors, and other additives.

Adherence to Applicable University Policies

Pepsi will further support the University's health and wellness goals by providing the University with a Product mix that allows the University and Participating Food Service Locations listed in Exhibit K, as well vending machines and other "autonomous" distribution and dispensing mechanisms throughout the Facilities, to meet the product, marketing, stocking, merchandising and related requirements of the UC Berkeley Food and Beverage Choices Policy.

Pepsi acknowledges and agrees that as part of this Agreement it commits to biannual reviews of the conformity of the Product mix it makes available to the University and the communications, promotional, stocking, and merchandising activities related to this Product mix with the University as part of this Agreement to:

- the UC Berkeley Food and Beverage Choices Policy

To facilitate this compliance, Pepsi will not:

- deliver new Products to the University without vigorously endeavoring to obtain affirmative consent from the University regarding the delivery of such Products.

Pepsi further acknowledges and agrees that it will take verifiable measures to address any discrepancies between its activities and updated University health and wellness-related policies.

EXHIBIT J
REPORTING AND REVIEW MEETINGS

Reporting:

1. On a quarterly basis, Pepsi will provide the University reporting that includes cumulative and itemized volume, spend, and sales data. Said data will include:

- product information (brand, size, format)
- channel (fountain, vending, bottles and cans, etc.)
- locations

The University prefers that this data include year-to-date comparisons between the then current reporting period and the equivalent previous year's reporting period.

In this context:

- "Volume" refers to the number of units of Pepsi products purchased by the University or sold through vending and similar machines throughout the Facilities.
- "Spend" refers to University purchases from Pepsi.
- "Sales" refers to direct sales to consumers via vending machines and similar "autonomous" technologies.

2. On an annual basis, Pepsi will provide the University:

- An annual version of the volume, spend, and sales reporting outlined above.
- Its annual ESG (or equivalent) reporting, such as the downloads available at https://www.pepsico.com/our-impact/sustainability/report-downloads as of June 2023.
  - Pepsi will provide the University with ESG-related metrics specific to Pepsi's operations with the University when such data is available.
- A formal update on its progress to provide the University with Gatorade and Naked Juice in non-single use plastic formats
- A list of collaboration opportunities wherein the University's research, innovation, or other expertise could be of potential benefit to Pepsi and/or the broader beverage industry's efforts to improve outcomes related to environmental sustainability, health and wellness, and other areas of mutual concern.

Meetings:

1. On a quarterly basis, representatives from Pepsi and the University will meet to discuss the ongoing relationship between the parties. The exact topics and formats of these meetings will be mutually determined by Pepsi and the University.

DocuSign Envelope ID: 5DB6E2D2-8865-4C2F-9B02-F38647AC4CB4

EXHIBIT K
PARTICIPATING FOOD SERVICE LOCATIONS

The following are locations at the Facilities that are Participating Food Service Locations, as defined in this Agreement.

Berkeley Dining:

- Dining Commons:
    - Café 3
    - Clark Kerr
    - Crossroads
    - Foothill
- Convenience Stores:
    - Bear Market
    - CK Cub
    - The Den
    - Cub Market
- Campus Restaurants:
    - Brown's
    - The Golden Bear Café
    - The Eateries at Student Union
    - The Pro Shop at RSF
- Berkeley Catering
- Training Table at California Memorial Stadium

Intercollegiate Athletics:

- California Memorial Stadium
- Clark Kerr Sand Courts
- Edwards Stadium
- Haas Pavilion
- Hellman Tennis Complex
- Legends Aquatic Center
- Levine-Fricke Field
- Spieker Aquatics Complex
- Stu Gordon Stadium
- T. Gary Rogers Rowing Center
- Underhill Field
- Witter Rugby Field

The Student Union:

- Bear's Lair
- Goldie's

DocuSign Envelope ID: 5DB6E2D2-8865-4C2F-9B02-F38647AC4CB4

International House:

- edmonds Cafe
- International House Dining Commons
- International House Catering

DocuSign Envelope ID: 5DB6E2D2-8865-4C2F-9B02-F38647AC4CB4

APPENDIX A
UC SUSTAINABLE PRACTICES POLICY

Sustainable Practices

**Responsible Officer:**	EVP – Chief Financial Officer
**Responsible Office:**	ES – Energy & Sustainability
**Issuance Date:**	3/10/2022
**Effective Date:**	3/10/2022
**Last Review Date:**	2/16/2022
**Scope:**	All Campuses, Health Locations, and the Lawrence Berkeley National Laboratory

**Contact:**	Matthew St. Clair
**Title:**	Director of Sustainability, UCOP
**Email:**	Matthew.StClair@ucop.edu
**Phone:**	(510) 287-3897

1. TABLE OF CONTENTS

I. POLICY SUMMARY **2**
II. DEFINITIONS **2**
III. POLICY TEXT **8**
- **A.** Green Building Design 8
- **B.** Clean Energy 9
- **C.** Climate Protection 10
- **D.** Sustainable Transportation 11
- **E.** Sustainable Building and Laboratory Operations for Campuses 12
- **F.** Zero Waste 12
- **G.** Sustainable Procurement 14
- **H.** Sustainable Foodservices 16
- **I.** Sustainable Water Systems 16
- **J.** Sustainability at UC Health 17
- **K.** General Sustainability Performance Assessment 18
- **L.** Health and Well-Being 18

IV. COMPLIANCE/RESPONSIBILITIES **18**
- **A.** Implementation 18
- **B.** Revisions 18
- **C.** Compliance 19

DocuSign Envelope ID: 5DB6E2D2-8865-4C2F-9B02-F38647AC4CB4

D. Reporting ... 19

V. PROCEDURES ... 19

A. Green Building Design ... 19

B. Clean Energy ... 23

C. Climate Protection ... 24

D. Sustainable Transportation ... 27

E. Sustainable Building and Laboratory Operations for Campuses ... 28

F. Zero Waste ... 29

G. Sustainable Procurement ... 31

H. Sustainable Foodservices ... 33

I. Sustainable Water Systems ... 34

J. Sustainability at UC Health ... 36

K. General Sustainability Performance Assessment ... 36

L. Health and Well-Being ... 37

VI. RELATED INFORMATION ... 37

VII. FREQUENTLY ASKED QUESTIONS ... 37

VIII. REVISION HISTORY ... 38

I. POLICY SUMMARY

The Sustainable Practices Policy ("Policy") establishes goals in 12 areas of sustainable practices: green building, clean energy, climate protection, transportation, sustainable operations, zero waste, procurement, foodservice, water, health care, performance assessment, and health and well-being.

II. DEFINITIONS

Association for the Advancement of Sustainability in Higher Education (AASHE): The higher education association that sets sustainability standards for universities and colleges. Its mission is to support sustainability in higher education through empowering faculty, administrators, staff, and students to be effective change agents and drivers of sustainability innovation.

Addressable Spend: Spend that can be impacted through sourcing activities. For the purposes of this Policy, it relates to the spend within a specific product or service category.
Adjusted Patient Day (APD): Inpatient Days x (Gross Patient Revenue/ Inpatient Revenue) where Gross Patient Revenue is Outpatient Revenue + Newborn Revenue + Inpatient Revenue.

California Building Code (CBC): This refers to the California Building Code, Title 24 portion of the California Code of Regulations

Clean Transportation Fuel: A clean transportation fuel is a fuel derived from a net carbon- neutral fuel source with a carbon intensity of zero or less. These transportation fuels are typically produced from nonpetroleum renewable sources. Common examples include natural gas or hydrogen derived from the capture of gases from sewage waste, manure collection, or green waste decomposition. A fuel's carbon intensity can vary based on how it is produced.

DocuSign Envelope ID: 5DB6E2D2-8865-4C2F-9B02-F38647AC4CB4

For a California Air Resources Board's maintained list of certified carbon intensities for alternative fuels, see the website LCFS Pathway Certified Carbon Intensities.

Climate Neutrality: Climate neutrality is a goal for the University to have net zero climate impacts from greenhouse gas (GHG) emissions attributed to scope 1 direct emission sources and scope 2 indirect emission sources as defined by The Climate Registry, and specific scope 3 emissions as defined by Second Nature's Carbon Commitment. This will be achieved by minimizing GHG emissions from these sources as much as possible and using carbon offsets or other measures to mitigate the remaining GHG emissions.

Combustion: As defined by CalRecycle, combustion is a rapid conversion of chemical energy into thermal energy. The reaction is exothermic. Organic matter is oxidized with sufficient air (or oxygen) for reactions to go to completion. The carbon and hydrogen are oxidized to carbon dioxide and water, respectively.

Construction and Demolition Waste: Waste generated by construction projects that do not occur every year or are not a result of regular operations and maintenance (e.g., building renovations or new construction).

Diversion from Landfill: Institutions divert materials from the landfill, combustion, or other non-allowable thermal conversion by recycling, composting, donating, reselling, or reusing.

Economically and Socially Responsible (EaSR) Spend: Spend on products or services supplied by a business holding one of the UC-recognized certifications listed in the UC Sustainable Procurement Guidelines.

Expanded Polystyrene (EPS): As defined by the City of San Francisco, blown polystyrene and expanded and extruded foams which are thermoplastic petrochemical materials utilizing a styrene monomer and processed by various techniques including fusioning polymer spheres (expanded bead polystyrene), injection molding, foam molding, and extrusion-blown molding (extruded foam polystyrene).

Fleet: University-owned or operated vehicles and mobility equipment (e.g., passenger vehicles, trucks, vans, shuttles, agricultural vehicles, marine equipment, etc.), including vehicles operated under contract with the University and for which the University/Campus maintains operational control.

Foodservice: Dining establishments such as cafeterias, restaurants, cafes, retail stores, or similar places where food or drink is stored, prepared, packaged, served, or sold for consumption on premises or elsewhere. This includes locations that administer meal plans. Health location foodservice is defined as cafeterias.

Foodware Accessory Items: All types of items usually provided alongside food in containers and cups, including utensils, chopsticks, napkins, cup lids, cup sleeves, food or beverage trays, condiment containers and saucers, straws, stirrers, and toothpicks.

Foodware: Products that are used to serve or transport food or beverages, including cups, bowls, plates, and hinged containers, as well as accessory items (see above definition). This does not include prepackaged, sealed food that is mass-produced by a third-party vendor off the premises for resale at University locations (e.g., grab-and-go items, such as prepackaged sandwiches and snacks resold in campus stores)

DocuSign Envelope ID: 5DB6E2D2-8865-4C2F-9B02-F38647AC4CB4

Green Lab Assessment Programs: A program that works with individual laboratories and researchers to inform, collect best practices, and assess areas for improvement in research efficiency, including engagement and targeted initiatives around efficiency in natural resources and other environmental issues. This assessment program could be based on the My Green Labs (MGL) Systemwide Checklist or another similar tool. The MGL checklist was developed based on best practices from several UC campuses as well as the expertise of My Green Lab

Gross Square Foot: Pursuant to the definition in the Facilities Inventory Guide (Appendix C, page C.19), gross square footage is the Outside Gross Area, or OGSF50, and equals the sum of Basic Gross Area (the sum of all areas, finished and unfinished, on all floors of an enclosed structure, for all stories or areas which have floor surfaces) + 50% Covered Unenclosed Gross Area (the sum of all covered or roofed areas of a building located outside of the enclosed structure). OGSF50 is also known as "California Gross."

Industrial Water: Water provided for specific industrial applications such as heating, cooling, or lubricating equipment.

Leadership in Energy and Environmental Design (LEED)™: Leadership in Energy and Environmental Design. LEED is a registered trademark of the U.S. Green Building Council (USGBC). This trademark applies to all occurrences of LEED in this document. LEED is a green building rating system developed and administered by the non-profit U.S. Green Building Council. The four levels of LEED certification, from lowest to highest, are Certified, Silver, Gold, and Platinum. LEED has several rating systems. This Policy refers to the following rating systems:

LEED for Interior Design and Construction (LEED-ID+C) for renovation projects;

LEED for Building Operations and Maintenance (LEED-O+M) for the ongoing operational and maintenance practices in buildings; and,

LEED for Building Design and Construction (LEED-BD+C) for new buildings and major renovations of existing buildings.

Locally Compostable: Products that can be composted in the local facilities that provide service to the campus. Acceptable products will vary by facility. Locally compostable may include but is not limited to products made of plastic, paper, wood, and bamboo. Compostable products must meet the criteria outlined in the Sustainable Procurement Guidelines.

Locally Recyclable: Products that can be recycled by the local facilities that provide service to the campus. Acceptable products will vary by facility.

Location: As used in this Policy, means all UC campuses, health locations, and the Lawrence Berkeley National Laboratory, as referred to in the "Scope" above.

Municipal Solid Waste: Garbage, refuse, sludges, and other discarded solid materials resulting from residential activities and industrial and commercial operations, which are legally accepted in CalRecycle permitted landfills. Municipal Solid Waste does not include any regulated hazardous/universal waste, medical waste, or other material used as Alternative Daily Cover (ADC); however, it does include construction and demolition waste, diverted recyclables and organic waste. Non-health location waste targets refer to municipal solid waste only. Health Locations waste targets use the Practice Greenhealth definition of "Total Solid Waste," see section III.J.

DocuSign Envelope ID: 5DB6E2D2-8865-4C2F-9B02-F38647AC4CB4

Organic: As defined by CalRecycle, material containing carbon and hydrogen. Organic material in municipal solid waste includes the biomass components of the waste stream as well as hydrocarbons usually derived from fossil sources (e.g., most plastics, polymers, the majority of waste tire components, and petroleum residues).

Packaging Foam: Any open or closed cell, solidified, polymeric foam used for cushioning or packaging, including: Ethylene-vinyl acetate (EVA) foam, Low-density polyethylene (LDPE) foam, Polychloroprene foam (Neoprene), Polypropylene (PP) foam, Polystyrene (PS) foam (including EPS, extruded polystyrene foam (XPS) and polystyrene paper (PSP)), Polyurethane (PU) foams, Polyethylene foams, Polyvinyl chloride (PVC) foam, and Microcellular foam. Not included are easily biodegradable, plant-based foams such as those derived from corn or mushrooms.

Partner for Change: An award given through Practice Greenhealth's Environmental Excellence Awards program that recognizes health care organizations that have implemented a significant number of environmental programs and who can demonstrate continuous improvement and expansion of these programs on the path to sustainability.

Plant-Based Foods: As defined by the Culinary Institute of America's Menus of Change program, these include fruits and vegetables (produce); whole grains; beans; other legumes (pulses), and soy foods; nuts and seeds; plant oils; herbs and spices; simple combinations of these foods and their derivatives, and vegetarian/vegan alternatives to meat and dairy.
Plant-Forward: As defined by the Culinary Institute of America's Menus of Change program, this represents a style of cooking and eating that emphasizes and celebrates, but is not limited to, plant-based foods—including fruits and vegetables (produce); whole grains; beans, other legumes (pulses), and soy foods; nuts and seeds; plant oils; and herbs and spices—and that reflects evidence-based principles of health and sustainability. Often used synonymously with "vegetable-centric," "vegetable-forward," and "plant-centric."

Plastic Bags: A carryout bag, regardless of the thickness of the material, made of plastic that is provided by a store or foodservice facility to a customer at the point of sale to hold customer's purchases. This does not include bags that are locally compostable.

Potable Water: Water that meets state water quality standards for human consumption**.**

Practice Greenhealth: The leading membership and networking organization for sustainable health care, delivering environmental solutions to hospitals and health systems across the United States.

Preferred Level Green Spend: The amount spent on products meeting the UC Preferred Level of environmental sustainability criteria as laid out in the UC Sustainable Procurement Guidelines.

Reclaimed or Recycled Water: Wastewater treated with the intention of reuse, including:

Direct Potable Reuse: Treated wastewater reused for human consumption.

Indirect Potable Reuse: Treated wastewater blended with groundwater or other water sources reused as potable or non-potable water.
Non-Potable Reuse: Treated wastewater reused for purposes other than human consumption, such as irrigation, fire suppression, and industrial processes.

DocuSign Envelope ID: 5DB6E2D2-8865-4C2F-9B02-F38647AC4CB4

Renewable Energy: Energy generated from inexhaustible sources, such as the sun or wind, or from sources that can quickly be replenished, such as biomass. For the purposes of this Policy, an energy source is renewable if it has been designated as such by the California Energy Commission (Renewables Portfolio Standard Guidebook).

Required Level Green Spend: The minimum spend that meets sustainability criteria required for a product or service category. For Required Level Green Spend criteria, see the UC Sustainable Procurement Guidelines.

Research Group: When counting the laboratories assessed via a green lab assessment program, a laboratory will be counted as a research group rather than by physical rooms. As defined in the Laboratory Hazard Assessment Tool (LHAT), this group includes the workers that report to one Principal Investigator (PI) or Responsible Person. While some PI's may have multiple groups, one assessment for the purposes of this Policy will include all the people working under one PI or Responsible Person, all of the rooms they occupy or share, and field sites, if any. Total number of PI's and Responsible People will be tracked according to LHAT or a similar tracking method at campuses not using LHAT. LHAT includes research and teaching laboratories.

Savings by Design: An energy efficiency program offered by California's four investor-owned utility companies and the Sacramento Municipal Utility District. Savings By Design provides design assistance, energy analysis, life cycle costing, and financial incentives for new construction and major renovation projects. The Savings By Design program is also known as the Non-Residential New Construction Program.

Single-Pass Cooling: Single-Pass or once-through cooling systems flow water through a piece of equipment to absorb heat and dispose of the water down the drain without recirculation. Replacing and managing these types of systems is a recommended best practice by the International Institute for Sustainable Laboratories (I^2SL), the U.S. Office of Energy Efficiency & Renewable Energy, and the EPA. Equipment typically using this type of cooling includes hydraulic equipment, distillation condensers, refrigeration condensers, air compressors, vacuum pumps, electron microscopes, mass spectrometers, lasers, helium recovery, and electro-magnets.

Single-Occupancy Vehicle (SOV): A vehicle driven by a single driver with no passengers. SOV percentages may separate the percentage of vehicle trips occurring in zero- or low- emission vehicles from carbon-fuel vehicles (e.g., SOV-standard fuel and SOV-alternative fuel).

Solicitation: The process of seeking information, bid proposals, and quotations from suppliers.

Sustainability Tracking, Assessment and Rating System (STARS): A transparent, self- reporting framework for colleges and universities to measure their sustainability performance. STARS provides a framework for understanding sustainability in all sectors of higher education through using a common set of measurements that enables meaningful comparisons over time and across institutions.

Sterilized Water: Water that has been cleaned to remove, deactivate, or kill microorganisms present that may be harmful to humans, primarily used in medical facilities and research.

Stormwater: Water that originates during precipitation events.

DocuSign Envelope ID: 5DB6E2D2-8865-4C2F-9B02-F38647AC4CB4

Strategic sourcing: A process designed to maximize the purchasing power of large, decentralized organizations, such as the University of California, by consolidating and leveraging common purchases.

Sustainable Food: Food and beverage purchases that meet the AASHE STARS Technical Manual's requirements for sustainably and ethically produced food for campuses and Practice Greenhealth's sustainable food for health locations.

Sustainable Procurement: Modified from the UK Government's Sustainable Procurement Task Force (2012) Purchasing that takes into account the economic, environmental, and socially responsible requirements of an entity's spending. Sustainable Procurement allows organizations to procure their goods and services in a way that achieves value for money on a whole-life basis in terms of generating benefits not only to the organization but also to society and the economy, while remaining within the carrying capacity of the environment.

Sustainable Water Systems: Water systems or processes that maximize water use conservation or efficiency, optimize water resource management, protect resources in the context of the local watershed, and enhance economic, social, and environmental sustainability while meeting operational objectives.

Takeback program: A program that allows customers to return used products or materials to either the producer or distributor for responsible re-use or recycling consistent with applicable state and federal laws. These programs encourage responsible design for disassembly and recyclability and protect the environment by keeping bulky or toxic products and packaging out of the waste stream.

Transportation Demand Management (TDM): The application of strategies and policies to reduce travel demand (specifically that of single-occupancy private vehicles). TDM programs may include car sharing (car share), carpools (rideshare), vanpools, bus pools, shuttles, transit, bicycle circulation systems, pedestrian circulation systems, emergency rides home, telecommuting, flexible schedules, parking management (amount, access, fees), etc.

Total Cost of Ownership (TCO): An analysis of cost that considers not only purchase price but also any costs associated with the acquisition, use, and disposal of the product. These costs may include some or all of the following: freight, taxes and fees, installation, operation/energy use, maintenance, warranty, collection, end-of-life disposal or recycling, as well as social or environmental costs, such as the cost of purchasing pollution offsets or monitoring labor practices.

UC Green Laboratories Action Plan: A document created with the goal of setting campus- specific targets, documenting the strengths and areas for improvement within sustainable operations of research laboratories via gap analysis, and outlining actions that can be implemented to further targets.

USGBC: U.S. Green Building Council. The USGBC is a membership-based non-profit organization dedicated to sustainable building design and construction and is the developer of the LEED building rating system.

Wastewater: Water that is discharged from domestic, industrial, or other use.

Watershed: In the context of this Policy, a watershed is the area of land that drains to a common waterway, such as a stream, lake, estuary, wetland, aquifer, bay, or ocean.

DocuSign Envelope ID: 5DB6E2D2-8865-4C2F-9B02-F38647AC4CB4

Water systems: Natural and/or human-made systems that provide water to and support the functions of watersheds and/or human communities.

Weighted Campus User (WCU): As defined in the current AASHE STARS Technical Manual. This calculation applies only to campuses and not to health locations or LBNL.

Zero-emissions vehicle (ZEV): As defined by the California Air Resources Board (CARB) ZEV program standards, a vehicle that emits no tailpipe pollutants (e.g., criteria air pollutants, precursors, or greenhouse gases) from the onboard source of power under any possible operational modes or conditions. Common examples include battery electric and fuel cell vehicles.

Zero waste: The University's zero waste goal is made up of incremental waste reduction and waste diversion targets. The University recognizes the attainment of reduction goals stated in this Policy and a 90% diversion of municipal solid waste as minimum compliance standard to be defined as zero waste for locations other than health locations.

III. POLICY TEXT

The University of California ("University") is committed to responsible stewardship of resources and to demonstrating leadership in sustainable business practices. The University's locations should be living laboratories for sustainability, contributing to the research and educational mission of the University. The goals outlined throughout these policy and procedures sections shall be applied within the constraints of research needs and budgetary requirements and in compliance with safe operating practices and all applicable rules, regulations and laws. Policy goals are presented below in twelve areas of sustainable practices.

A. Green Building Design

1. New Buildings

   a. All new building projects, other than acute care facilities, will be designed, constructed, and commissioned to outperform the California Building Code (CBC) energy-efficiency standards by at least 20% or meet the whole-building energy performance targets listed in Table 1 of Section V.A.1. The University will strive to design, construct, and commission buildings that outperform CBC energy efficiency standards by 30% or more or meet the stretch whole-building energy performance targets listed in Table 1 of Section V.A.1, whenever possible within the constraints of program needs and standard budget parameters.

   b. Acute care/hospital facilities and medical office buildings will be designed, constructed, and commissioned to outperform ASHRAE 90.1 - 2010 by at least 30% or meet the whole-building energy performance targets listed in Table 2 in Section V.A.1.

   c. No new building or major renovation that is approved after June 30, 2019, will use onsite fossil fuel combustion (e.g., natural gas) for space and water heating (except those projects connected to an existing campus central thermal infrastructure). Projects unable to meet this requirement will document the rationale for this decision, as described in Section V.A.1.d.

   d. All new buildings will achieve a USGBC LEED "Silver" certification at a minimum. All

DocuSign Envelope ID: 5DB6E2D2-8865-4C2F-9B02-F38647AC4CB4

new buildings will strive to achieve certification at a USGBC LEED "Gold" rating or higher, whenever possible within the constraints of program needs and standard budget parameters.

e. The University of California will design, construct, and commission new laboratory buildings to achieve a minimum of LEED "Silver" certification. Design, construction, and commissioning processes will strive to optimize the energy efficiency of systems not addressed by the CBC energy efficiency standards.

f. All new building projects will achieve at least two points within the available credits in LEED-BD+C's Water Efficiency category (in support of section III.I.) and prioritize earning waste reduction and recycling credits (per section V.F.)

2. Building Renovations

a. Major Renovations of buildings are defined as projects that require 100% replacement of mechanical, electrical, and plumbing systems and replacement of over 50% of all non-shell areas (interior walls, doors, floor coverings, and ceiling systems) will at a minimum comply with III.A.1.d. or III.A.1.e. Such projects will outperform CBC Title 24, Part 6, currently in effect, by 20%. This does not apply to acute care facilities.

b. Acute care facilities and medical office buildings undertaking major renovations, as defined above, will outperform ASHRAE 90.1- 2010 by 30%.

c. Renovation projects with a project cost of $5 million or greater (CCCI 5000) that do not constitute a Major Renovation as defined in item III.A.2.a. will at a minimum achieve a LEED-ID+C Certified rating and register with the utilities' Savings by Design program, if eligible. This does not apply to acute care facilities.

B. Clean Energy

In support of the climate neutrality goals outlined in Section C of this Policy, the University of California is committed to reducing its greenhouse gas emissions by reducing energy use and switching to clean energy supplies.

1. Energy Efficiency

Each location will implement energy efficiency actions in buildings and infrastructure systems to reduce the location's energy use intensity by an average of at least 2% annually.

2. On-campus Renewable Electricity

Campuses and health locations will install additional on-site renewable electricity supplies and energy storage systems whenever cost-effective and/or supportive of the location's Climate Action Plan or other goals.

3. Off-campus Clean Electricity

By 2025, each campus and health location will obtain 100% clean electricity. The UC Clean Power Program will provide 100% clean electricity to participating locations.

4. On-campus Combustion

By 2025, at least 40% of the natural gas combusted on-site at each campus and

DocuSign Envelope ID: 5DB6E2D2-8865-4C2F-9B02-F38647AC4CB4

health location will be biogas.

C. Climate Protection

1. Greenhouse Gas Emissions

Each campus and the UC Office of the President will develop strategies for meeting the following UC goals:

a. Achieve climate neutrality from scope 1 and 2 sources by 2025

b. Achieve climate neutrality from specific scope 3 sources (as defined by Second Nature's Carbon Commitment) by 2050 or sooner

c. Maintain greenhouse gas (GHG) emissions at or below 1990 levels, pursuant to the California Global Warming Solutions Act of 2006.

For purposes of this section, campuses will include their related health location for all goals. GHG emissions reduction goals pertain to emissions of the six Kyoto greenhouse gases (carbon dioxide, methane, nitrous oxide, sulfur hexafluoride, hydrofluorocarbons, and perfluorocarbons) originating from all scope 1 and scope 2 sources as specified by the Climate Registry and from scope 3 emissions as specified by Second Nature's Carbon Commitment, which includes air travel paid through the institution; and commuting to and from campus by students, faculty and other academic appointees, and staff. These goals will be pursued while maintaining the research, education, and public service missions of the University. Campuses subject to the United States Environmental Protection Agency (USEPA) Greenhouse Gas Reporting Program, California Air Resources Board (CARB) Mandatory Greenhouse Gas Emissions Reporting, or participation in the CARB Cap-and-Trade Program will perform to those regulatory requirements.

2. Offsets

a. The University will prioritize direct reductions of its covered scope 1, 2, and 3 emissions. This Policy does not require the University, as a system and as individual campuses and units, to purchase carbon offsets to meet their carbon neutrality goals; instead, it sets priorities and minimum standards if they decide to purchase offsets. In meeting the UC Sustainable Practices Policy climate goals as outlined in section III.C., the University will use offsets as a transitional strategy while implementing all feasible reductions in its scope 1, 2, and 3 emissions. The University will reevaluate and update section III.C and V.C of the Sustainable Practices Policy by 2025.

b. The University will only use high-quality offset credits to meet its climate protection goals, beyond its requirements under California's cap-and-trade program and will draw on the University's academic capacity to vet the quality of all voluntary offset credits it uses.

c. To align its voluntary offset program with its research, education, and public service mission, the University will choose offset projects that demonstrate or advance scalable climate solutions aligned with a path towards deep decarbonization; prioritize projects that advance University research and support student education; prioritize projects with health and social justice benefits, and benefits to the UC

DocuSign Envelope ID: 5DB6E2D2-8865-4C2F-9B02-F38647AC4CB4

community and communities surrounding the campuses; and prioritize projects with the potential for climate benefits well beyond the credited reductions, recognizing the urgency of near-term reductions. The University will analyze the ecological, health, social, and human rights impacts of its offset decisions to avoid negative outcomes for low-income communities, communities of color, and other marginalized populations and to prioritize projects that benefit these communities.

d. The University will develop and implement its voluntary offset procurement strategy in a way that advances understanding of and models how institutions of higher education and in other sectors can use offsets as an effective climate mitigation strategy aligned with their institutional mission.

D. Sustainable Transportation

The University will implement transportation programs and greenhouse gas (GHG) emission reduction strategies that reduce the environmental impacts from commuting, fleet and business air travel related to achieving the Climate Protection section of this Policy (see Section III.C.).

1. Each location will reduce GHG emissions from its fleet and report annually on its progress. Locations will implement strategies to reduce emissions from University- owned or operated fleet vehicles to align with UC's 2025 carbon neutrality goals (as defined in the Climate Protection sections of this Policy). Carbon neutral fleets can be achieved if vehicles produce no tailpipe emissions, use a clean transportation fuel, and/or if carbon offsets are purchased.

   To support this goal, each location will ensure that:

   a. After July 1, 2023, zero-emission vehicles, plug-in hybrid, or dedicated clean transportation fueled vehicles will account for at least 50% of all vehicle acquisitions (including both leased and purchased vehicles).

   b. All sedans and minivan acquisitions will be zero-emission or plug-in hybrid vehicles, except for public safety vehicles with special performance requirements.

   c. In applications where zero-emission vehicles are not available, regardless of vehicle size class, the use of clean transportation fuels and other low-emission fuels will be prioritized

   Furthermore:

   d. Any carbon offsets purchased to meet the carbon neutrality goal will be coordinated with the location's Office of Sustainability, will support the location's overall carbon neutrality strategy, and will follow the guidelines laid out in the Climate Protection section of this Policy (see Section III.C.).

   e. Vehicle acquisitions plans should meet the State's goal (outlined in Executive Order N-79-20) that all new passenger cars and light-duty trucks (under 8,500 lbs.) acquired after January 1, 2035, and all medium-and heavy-duty vehicles acquired or operated after January 1, 2045, will be zero-emission.

   Lawrence Berkeley National Laboratory will follow federal fleet requirements in the case where federal and UC fleet requirements conflict.

DocuSign Envelope ID: 5DB6E2D2-8865-4C2F-9B02-F38647AC4CB4

2. The University recognizes that single-occupant vehicle (SOV) commuting is a primary contributor to commute-related GHG emissions and localized transportation impacts.
   a. By 2025, each location will strive to reduce its percentage of employees and students commuting by SOV by 10% relative to its 2015 SOV commute rates.
   b. By 2050, each location will strive to have no more than 40% of its employees and no more than 30% of all employees and students commuting to the location by SOV.
3. Recognizing that flexible work arrangements, including telecommuting, are a low-cost, effective way to reduce emissions and carbon footprint, each location should review and update local employee telecommute and flexible work policies, guidelines, procedures, and other applicable documents to normalize and promote telecommuting options and other flexible scheduling, as aligned appropriately based on business needs.
4. Consistent with the State of California goal of increasing alternative fuel – specifically electric – vehicle usage, the University will promote purchases and support investment in alternative fuel infrastructure at each location.
   a. By 2025, each location will strive to have at least 4.5% of commuter vehicles be zero-emissions vehicles (ZEV).
   b. By 2050, each location will strive to have at least 30% of commuter vehicles be ZEV.
5. Each location will develop a business-case analysis for any proposed parking structures serving University affiliates or visitors to campus to document how a capital investment in parking aligns with each campus' Climate Action Plans and/or sustainable transportation policies.

E. Sustainable Building and Laboratory Operations for Campuses

1. Each campus will seek to certify as many buildings as possible through the LEED-O+M rating system within budgetary constraints and eligibility limitations.
2. All campuses will maintain an ongoing Green Lab Assessment Program supported by a department on campus to assess the operational sustainability of research groups and the laboratories and other research spaces.
   a. At least one staff or faculty member from the campus must have the role of managing the Green Lab Assessment Program.
   b. Any green lab assessment programs and related efforts will adhere to all relevant UC, state and national policies and laws. Safety will never be compromised to accommodate sustainability goals.
   c. All campuses will maintain a UC Green Laboratories Action Plan.

F. Zero Waste

1. The University will achieve zero waste through prioritizing waste reduction in the following order: reduce, reuse, and then recycle and compost (or other forms of organic recycling) as described in section V.F.6. Minimum compliance for zero waste, at all locations other than health locations, is as follows:
   a. Reduce per capita municipal solid waste generation by:

DocuSign Envelope ID: 5DB6E2D2-8865-4C2F-9B02-F38647AC4CB4

i. 25% per capita from FY2015/16 levels by 2025

ii. 50% per capita from FY2015/16 levels by 2030.

b. Divert 90% of municipal solid waste from the landfill.

2. The University supports the integration of waste, climate and other sustainability goals, including the reduction of embodied carbon in the supply chain through the promotion of a circular economy and the management of organic waste to promote atmospheric carbon reduction. In support of this goal, waste reporting will include tracking estimated scope 3 greenhouse gas emissions.

3. The University prohibits the sale, procurement, or distribution of packaging foam, such as food containers and packaging material, other than that utilized for laboratory supply or medical packaging and products. The University seeks to reduce, reuse, and find alternatives for packaging foam used for laboratory and medical packaging products.

a. No packaging foam or expanded polystyrene (EPS) will be used in foodservice facilities for takeaway containers.

For implementation guidelines related to the procurement of goods for University of California campuses, reference the University of California Sustainable Procurement Guidelines.

4. The University is committed to the reduction and elimination of single-use items in line with the University's and the State of California's Zero Waste goals and in recognition of the severe environmental impact single-use products have globally. In recognition of this commitment, locations will reduce single-use products by taking the following actions:

a. Eliminate plastic bags in all retail and foodservice establishments in campus facilities or located on University owned land no later than January 1, 2021

b. Replace disposable single-use plastic foodware accessory items in all foodservice facilities with reusables or locally compostable alternatives and provide only upon request no later than July 1, 2021

c. Provide reusable foodware items for food consumed onsite at dine-in facilities and to-go facilities no later than July 1, 2022.

d. Replace single-use plastic foodware items with reusable or locally compostable alternatives at to-go facilities no later than July 1, 2022

e. Phase out the procurement, sale and distribution of single-use plastic beverage bottles. Non-plastic alternatives will be locally recyclable or compostable.

i. Foodservice facilities will provide alternatives no later than January 1, 2023.

ii. Locations are encouraged to prioritize the installation of water refill stations to support the transition from single-use plastics to reusables.

iii. Locations will consider eliminating single-use plastic beverage bottles when contracting with suppliers, or upon contract renewal and/or extension if current contract terms prohibit (e.g., vending machines, departmental purchases, etc.).

f. When selecting prepackaged, sealed food that is mass produced off premises and

DocuSign Envelope ID: 5DB6E2D2-8865-4C2F-9B02-F38647AC4CB4

resold at University locations (e.g., grab-and-go items, such as chips, candy, prepackaged sandwiches, etc.), preference should be given in contract award and negotiations to suppliers that utilize locally compostable or locally recyclable packaging options.

This Policy section (III.F.4.) also applies to third-party foodservice facilities that lease space or provide contracted services at locations. Locations will include these Policy provisions in lease language as new leases and contracts are negotiated or existing leases are renewed and work to incorporate these practices, as much as possible, within the timeframe of current leases. When procuring catering services, where possible, select providers that can provide alternatives to single-use plastics.

G. Sustainable Procurement

Recognizing the substantial impact that procurement decisions have on the environment, society, and the economy, the University of California will maximize its procurement of sustainable products and services. The goals outlined throughout these policy and procedures sections will be applied within the constraints of research needs and budgetary requirements and in compliance with all applicable rules, regulations, and laws.

1. The University values the health and wellbeing of its students, staff, faculty and other academic appointees, visitors, and suppliers. The University seeks to provide healthy and accessible conditions for the communities it serves, and this will be considered as a fundamental factor when making procurement decisions. Where functional alternatives to harmful products or impacts exist, they are to be strongly preferred.

2. Per III.F.1. the University prioritizes waste reduction in the following order: reduce, reuse, and then recycle. Accordingly, sustainable procurement will look to reduce unnecessary purchasing first, then prioritize the purchase of surplus or multiple-use products, before looking at recyclable or compostable products.

3. The University's sustainable purchasing requirements (detailed in the UC Sustainable Procurement Guidelines) are:

   a. 100% compliance with Required Level Green Spend criteria within three fiscal years of the addition of those products and/or product categories to the Guidelines.

   b. 25% Preferred Level Green Spend as a total percentage of spend per product category; target to be reached within three fiscal years after a category is added to the Guidelines.

   c. 25% Economically and Socially Responsible Spend as a total percentage of addressable spend; target to be reached within five fiscal years of adoption of this section in the Guidelines.

4. The University's sustainable purchasing reporting requirements are:

   a. Reporting on percent Preferred Level Green Spend beginning at the close of the first full Fiscal Year after a category is added to the Guidelines.

   b. Reporting on percent Economically and Socially Responsible Spend beginning at the close of Fiscal Year 2018/19.

5. Each University's Procurement department will integrate sustainability into its processes

DocuSign Envelope ID: 5DB6E2D2-8865-4C2F-9B02-F38647AC4CB4

and practices, including competitive solicitations, to satisfy the sustainable purchasing goals outlined above for products, as well as for the procurement of services. The University will do so by:

a. Allocating a minimum of 15% of the points utilized in solicitation evaluations to sustainability criteria. Criteria may include, but are not limited to, sustainable product attributes, supplier diversity, supplier practices, contributions to health and wellbeing, and materials safety. Exceptions to this Policy may only be granted by the appropriate Policy Exception Authority. Decisions to grant an exception will be made in the context of a location's need to support teaching, research and public service when there is a demonstrable case that the inclusion of a minimum of 15% of the points utilized in solicitation evaluation for sustainability criteria will conflict with the project teams' ability to execute a competitive solicitation.

b. Supporting outreach, education, and providing equal access to small, diverse, and disadvantaged suppliers for all applicable University procurement opportunities in accordance with BUS-43 policy.

c. Comparing the Total Cost of Ownership when evaluating costs for goods and services in the selection of suppliers, whenever feasible.[1]

d. Targeting sustainable products and services for volume-discounted pricing to make less competitive or emerging sustainable products and services cost-competitive with conventional products and services.

e. Leveraging its purchasing power and market presence to develop sustainable product and service options where not already available.

f. Requiring packaging for all products procured by the University be designed, produced, and distributed to the end-user in a sustainable manner.

g. Contracting with suppliers of products (e.g., electronics, furniture, lab consumables) that have established (preferably non-manufacturer specific) end-of-life reuse, recycling, and/or takeback programs at no extra cost to the University, and in compliance with applicable federal, state, and University regulations regarding waste disposal.

h. Requiring sustainability-related purchasing claims to be supported with UC- recognized certifications and/or detailed information on proven benefits, durability, recycled content, and recyclability properties, in accordance with the Federal Trade Commission's (FTC) Green Guides for the use of environmental marketing claims.

i. Working with its suppliers to achieve greater transparency and sustainable outcomes throughout the supply chain. This may include maximizing the procurement of products that optimize the use of resources from extraction through

[1] Public Contract Code§ 10507.8 states: "As provided for in this article, when the University of California determines that it can expect long-term savings through the use of life cycle cost methodology, the use of more sustainable goods and materials, and reduced administrative costs, the lowest responsible bidder may be selected on the basis of the best value to the University. To implement this method of selection, the Regents of the University of California will adopt and publish policies and guidelines for evaluating bidders that ensure that best value selections by the University are conducted in a fair and impartial manner."

DocuSign Envelope ID: 5DB6E2D2-8865-4C2F-9B02-F38647AC4CB4

manufacturing and distribution (e.g., EPA's SmartWay Program).

6. All procurement staff will consult the UC Sustainable Procurement Guidelines document for minimum mandatory sustainability requirements to be included in solicitations for a given product or service category.

H. Sustainable Foodservices

1. Campus and Health Location Foodservice Operations

a. Food Procurement

Each campus foodservice operation will strive to procure 25% sustainable food products by the year 2030 as defined by AASHE STARS, and each health location foodservice operation will strive to procure 30% sustainable food products by the year 2030 as defined by Practice Greenhealth, while maintaining accessibility and affordability for all students and health location's foodservice patrons.

b. Education

Each campus and health location will provide patrons and foodservice staff with access to educational and training materials that will help support their food choices.

c. Menu Development

Each campus and health location will strive to reduce greenhouse gas emissions of their food purchases through globally-inspired, culturally-acceptable plant-forward menus.

i. Campuses and health centers will establish a baseline and goal in 2020.

Progress will be tracked annually by reporting the percentage of plant-based foods procured beginning in 2021.

2. Foodservice Operations in Leased Locations:

a. Foodservice operations leased in campuses and health locations owned by the University of California and contractors providing foodservices in campus and health locations will strive to meet the policies in III.H.1.a-c.

b. Campuses and health locations will include Section H of this Policy in lease language as new leases and contracts are negotiated or existing leases are renewed. However, campus and health locations will also work with tenants to advance sustainable foodservice practices as much as possible within the timeframe of current leases.

I. Sustainable Water Systems

With the overall intent of achieving sustainable water systems and demonstrating leadership in the area of sustainable water systems, the University has set the following goals applicable to all locations:

1. Locations will reduce growth-adjusted potable water consumption 20% by 2020, and 36% by 2025, when compared to a three-year average baseline of FY2005/06, FY2006/07, and FY2007/08. Locations that achieve this target early are encouraged to set more stringent goals to further reduce potable water consumption.

DocuSign Envelope ID: 5DB6E2D2-8865-4C2F-9B02-F38647AC4CB4

a. Each campus will strive to reduce potable water used for irrigation by converting to recycled water, implementing efficient irrigation systems, planting drought-tolerant landscaping (including California native plants where feasible and appropriate), and/or removing turf.

2. Each location will develop and maintain a Water Action Plan that identifies long-term strategies for achieving sustainable water systems.

3. Each campus will identify once-through cooling systems, constant flow sterilizers, constant-flow autoclaves and other water-to-waste cooling systems. Each campus will develop and implement plans for eliminating or replacing these systems with recirculating systems or other means of cooling that do not drain water to waste after one use.

4. New equipment requiring liquid cooling will be connected to an existing recirculated building cooling water system, new local chiller vented to building exhaust or outdoors, or to the campus chilled water system through an intervening heat exchange system, if available.

   a. Once-through or single-pass cooling systems will not be allowed for soft-plumbed systems using flexible tubing and quick-connect fittings for short-term research settings.

   b. If no alternative to single-pass cooling exists, water flow must be metered, automated and controlled to reduce water waste.

5. Required water efficiency measures applicable to building projects are outlined in Section A of this Policy on Green Building Design, New Building.

6. Guidelines for the sustainable procurement of water fixtures, as applicable, are listed in the UC Sustainable Procurement Guidelines.

J. Sustainability at UC Health

1. Health locations will achieve Practice Greenhealth's award "Greenhealth Partner for Change."

2. Health locations will achieve a target of 25lbs of total solid waste as defined by Practice Greenhealth per Adjusted Patient Day by 2025 and strive for 20lbs of total waste per Adjusted Patient Day by 2030. In meeting these goals, Health locations will follow the provisions outlined in section F of this Policy on Zero Waste, including limiting combustion and reducing the use of foam and single-use products.

   a. Practice Greenhealth defines total solid waste as municipal solid waste as well as all forms of regulated waste. This includes but is not limited to regulated medical waste, biohazardous waste, pharmaceutical waste, and universal waste. It does not include construction and demolition waste.

3. In line with campus targets, health locations will reduce growth-adjusted potable water consumption 20% by 2020 and 36% by 2025, when compared to a three-year average baseline of FY2005/06, FY2006/07, and FY2007/08.

4. Acute care/hospital facilities and medical office buildings in health locations will be designed, constructed and commissioned, or renovated as outlined in Section A of this

DocuSign Envelope ID: 5DB6E2D2-8865-4C2F-9B02-F38647AC4CB4

Policy on Green Building Design.

5. Health locations will strive to procure 30% sustainable food products by the year 2030 as defined by Practice Greenhealth and outlined in Section H of this Policy on Sustainable Foodservices.

K. General Sustainability Performance Assessment

1. All undergraduate campuses must maintain a certified AASHE STARS report.
2. All campuses must achieve a Silver STARS rating and strive for Gold by 2023.

L. Health and Well-Being

Health, equity, and the environment, including climate, are deeply interconnected, thus health, inequity, and environmental and climate change require intersectoral and collaborative solutions. Healthful food, healthy buildings, and active transportation are just some examples in which health, sustainability, and equity are synergistic. The Healthy Campus Network (HCN) leadership will use a Health in All Policies[2] framework and broad stakeholder engagement to better address health inequities; to support a culture of health for all faculty, staff, and students; to foster community collaborations across the UC system and California; and to meet the policy goals outlined below.

1. By the end of 2022, the HCN will review the strengths and gaps in the UC Sustainable Practices Policy and make recommendations for integration based on:
   a. Environmental and human health co-benefits,
   b. Social, physical, and emotional well-being, and
   c. Health equity.
2. By the end of 2021, the HCN will review and revise healthy vending goals with stakeholders to propose for inclusion in this Policy.
3. By the end of 2021, the HCN will review the chemicals of concern criteria detailed in the Sustainable Procurement Guidelines and make recommendations for the inclusion of specific Policy targets.

IV. COMPLIANCE/RESPONSIBILITIES

A. Implementation

The Executive Vice President - Chief Financial Officer is the Responsible Officer for this Policy. The UC Sustainability Steering Committee, which is chaired by the Executive Vice President - Chief Financial Officer, provides oversight for all aspects of the Policy.

B. Revisions

The President is the approver of this Policy and has the authority to approve or delegate the approval of revisions to the Policy.

[2] Rudolph, L., Caplan, J., Ben-Moshe, K., & Dillon, L. (2013). Health in All Policies: A Guide for State and Local Governments. Washington, DC and Oakland, CA: American Public Health Association and Public Health Institute

DocuSign Envelope ID: 5DB6E2D2-8865-4C2F-9B02-F38647AC4CB4

The systemwide Working Group corresponding to each section of the Policy recommends Policy revisions to the UC Sustainability Steering Committee and Executive Vice President - Chief Financial Officer. Proposed previsions accepted by the UC Sustainability Steering Committee and the Executive Vice President - Chief Financial Officer will then be recommended to the President for approval or to the appropriate delegated authority, as stated above.

The Sustainable Practices Policy will be reviewed, at a minimum, once every three years with the intent of developing and strengthening implementation provisions and assessing the influence of the Policy on existing facilities and operations, new capital projects, plant operating costs, fleet and transportation services, and accessibility, mobility, and livability. The University will provide for ongoing active participation of students, faculty and other academic appointees, administrators, and external representatives in further development and implementation of this Policy.

C. Compliance

Chancellors and the Lawrence Berkeley National Laboratory Director are responsible for implementation of the Policy in the context of individual building projects, facilities operations, etc. An assessment of location achievements with regard to the Policy is detailed in an annual report to the Regents. The internal audit department may conduct periodic audits to assess compliance with this Policy.

D. Reporting

On an annual basis, the President will report to the Regents on the University's sustainability efforts in each area of the Policy. Unless otherwise specified, reporting on progress on each section of this Policy will be to UCOP as part of the development of the Annual Report on Sustainable Practices.

V. PROCEDURES

A. Green Building Design

1. New Buildings and Major Renovations

   a. Projects will utilize the versions of the CBC energy efficiency standards and LEED- BD+C that are in effect at the time of the first submittal of "Preliminary Plans" (design development drawings and outline specifications) as defined in the State Administrative Manual.[3]

   b. If eligible, all new buildings and major renovations (as defined in III.A) will register with the Savings By Design program to document compliance with the requirement to outperform CBC energy efficiency standards by at least 20%.

   c. Projects other than acute care facilities that opt to use energy performance targets for compliance with III.A.1.a. will at a minimum use the whole-building energy

[3] The State Administrative Manual is a reference source for statewide policies, procedures, regulations and information developed and issued by authoring agencies such as the Governor's Office, Department of General Services, Department of Finance , and Department of Personnel Administration.

DocuSign Envelope ID: 5DB6E2D2-8865-4C2F-9B02-F38647AC4CB4

performance target listed below that corresponds to the year of the project's budget approval.

i. The whole-building energy performance target is expressed as a percentage of the sum of the Annual Electricity and Annual Thermal targets (converted to kBtu/gsf-yr) as developed for UC Building 1999 Energy Benchmarks by Campus, in Sahai, et al. 2014 and updated with a new "100% Lab Space" use type in the spreadsheet 2016 Whole-Building Quantitative Energy Performance Targets (2020 update).[4]

1.1 Table 1

Calendar Years	Compliance Target	Stretch Target
2015-16	65%	50%
2017-18	60%	45%
2019-20	55%	40%
2021-22	50%	35%
2023-24	45%	30%
2025 or after	40%	25%

d. Projects will report their target energy use and how much they anticipate exceeding the CBC energy-efficiency standards (campuses), ASHRAE 90.1 - 2010 (Health Locations), or the UC Building Benchmarks at plan phase (P-Phase) approval. This information will be confirmed with modeled energy estimates at approval of the start of construction (completion of the W-Phase). Final efficiency will be reported at closeout (generally a year after the building has been occupied).

e. Decisions affecting energy efficiency, fossil fuel use, and connection to existing central thermal services will be made in the context of the location's climate action plan. Where on-site fossil fuel combustion within the building is deemed necessary, the rationale for this decision will be documented as part of the existing project approval process. The submittal should include the following:

i. An estimate of annual electricity and gas use for the project as well as the project's target design energy use in thousand British thermal units (kBtu) per square foot.

ii. An explanation of why fossil fuel combustion is required for the project and what other alternatives were evaluated.

iii. An analysis explaining why fossil-fuel combustion is the most cost-effective energy source for the identified project-specific applications.

iv. A plan to mitigate, by 2025, the associated greenhouse gas emissions in accordance with the location's Climate Action Plan.

[4] The "UC Building 1999 Energy Benchmarks by Campus" and "2016 Whole-Building Quantitative Energy Performance Targets (2020 update)" documents can be found at https://www.ucop.edu/sustainability/policy-areas/green-building/index.html.

DocuSign Envelope ID: 5DB6E2D2-8865-4C2F-9B02-F38647AC4CB4

This documentation is part of the broader project approval process and does not require separate UCOP approval. Draft information should be submitted prior to budget approval as part of a Project Planning Guide, Delegated Authority Project Certification Checklist or related ancillary document. This information should be updated prior to design approval.

f. Acute care facilities and medical office buildings opting to use energy performance targets for compliance with III.A.1.b. will at a minimum use the whole-building energy performance target listed in Table 2 below. The whole-building energy performance target is expressed as a percentage of the sum of the Annual Electricity and Annual Thermal targets (converted to kBTU/gsf-yr) based on ASHRAE (2012) Advanced Energy Design Guidelines for Large Hospitals.

1.2 Table 2

	**Acute Care**			**Medical Office Buildings**		
	**Benchmark Average**	**Target**	**Stretch Target**	**Benchmark Average**	**Target**	**Stretch Target**
UC Davis Health	230	160	115	85	60	43
UC Irvine Health	230	160	115	80	56	40
UCLA Health	230	160	115	80	56	40
UC San Diego	230	160	115	80	56	40
UC San Francisco Health	230	160	115	80	56	40

g. Locations will demonstrate compliance based on the results of energy modeling that represents a best estimate of as-operated, whole-building energy use, before accounting for on-site energy generation. Targets are intended to be verifiable in actual operation following building occupancy.

Projects are also required to model and report on the following metrics:

- annual electricity consumption (kWh/gsf/yr)
- annual thermal consumption (therms/gsf/yr)
- peak electricity (W/gsf)
- peak chilled water (tons/kgsf) (if applicable)
- peak thermal (therms/hr/kgsf)

The following very high-intensity process loads may be subtracted out of the total building energy use intensity if they can be metered separately:

- Clean room

- Data center
- Micro-chip fabrication
- Accelerator (e.g., laser, light source)
- Bio-safety level III Laboratory
- Magnetic Resonance Imaging (MRI)
- Positron Emission Tomography (PET)
- Computer Tomography (CT)
- Pharmacies

If a building has more than 6 Operating Rooms (ORs), additional ORs (defined as any ORs beyond the baseline of 6 ORs) may be subtracted out of total building energy use intensity if they meet the following two requirements:

i. OR heating, ventilation and air conditioning (HVAC) is metered separately; and,

ii. A commitment is made by an appropriate official within the hospital's administration to implement an OR HVAC setback program in the subtracted ORs.

h. Locations are encouraged to coordinate with local water districts in efforts to conserve water and to meet reduced water use goals of the local districts.

2. Privatized Development

a. All privatized development of New Buildings or Major Renovations on University- owned land that is constructed in whole or in substantial part for University-related purposes (i.e., in furtherance of the University's mission, both programmatic and auxiliary uses), and build-to-suit projects not on University-owned land constructed for University-related purposes, will comply with section III.A. of this Policy. The provisions of this subsection apply regardless of the business relationship between the parties (i.e., whether a gift, acquisition, ground lease and/or lease).

3. Building Renovations

a. At budget approval, all renovation projects should include a list of sustainable measures under consideration.

b. For all improvement projects in spaces leased or licensed by the Regents to be used for University-related purposes for a term of greater than 12 months, locations will strive to comply with the appropriate Policy requirements in III.A.2.

4. Waiver Conditions Applicable to all Projects

a. Waivers will only be granted in exceptional circumstances and will not be considered if the project negatively impacts the ability to comply with the goals of this Policy, in particular the goal of achieving carbon neutrality by 2025.

b. Any proposed waiver from section III.A. of the Policy may be requested administratively from the UCOP Executive Director of Capital Programs prior to first project approval.

DocuSign Envelope ID: 5DB6E2D2-8865-4C2F-9B02-F38647AC4CB4

c. New Building and Major Renovation projects applying for an exception from section III.A.1.d. of this Policy should strive to achieve a USGBC LEED "Certified" rating. New building and renovation projects that are unable to achieve a USGBC LEED "Certified" rating will submit a request for an exception with a LEED scorecard and supporting documentation to the UCOP Executive Director of Capital Programs, showing the credits that the project would achieve.

d. Such waiver requests will indicate the applicable section of the Policy and/or Procedures; the proposed solution; and demonstrate equivalency with Policy intent.

5. General/Miscellaneous

a. The University will develop a program for sharing best practices.

b. The University will incorporate the requirements of sections III.A. and V.A. into existing training programs, with the aim of promoting and maintaining the goals of the Policy.

c. The University planning and design process will include explicit consideration of life cycle cost along with other factors in the project planning and design process, recognizing the importance of long-term operations and maintenance in the performance of University facilities.

d. The University will work closely with the USGBC, I²SL, the Department of Energy, the U.S. Environmental Protection Agency, state government, and other organizations to facilitate the improvement of evaluation methodologies to address University requirements.

B. Clean Energy

1. Energy Efficiency: The energy efficiency goal follows the spirit of the U.S. Department of Energy's Better Building Challenge. Each location's percent reduction in energy use intensity (EUI) will be reported annually based on the sum of weather-adjusted energy use divided by the sum of the maintained gross square footage (OGSF50). The average annual reduction will be calculated using an established baseline as detailed in the UC EUI Tracking Methods and References. UCOP will use energy usage data from the systemwide purchased utility database for reporting campus energy use intensity, based on the campus-specified set of utility accounts and associated maintained gross square footage. Electric and gas site energy will be converted to kBTU and normalized for weather. Policy goals will be evaluated and adjusted as appropriate following the 2025 reporting year.

2. On-campus Renewable Energy

a. Each location will determine the appropriate mix of measures to be adopted within its clean energy portfolio. The capacity to adopt these measures is driven by technological and economic factors, and each location will need to reevaluate its mix of energy measures regularly.

b. Locations will periodically evaluate the feasibility of new on-site renewable electricity projects. The financial evaluation of these projects will fully account for the anticipated avoided costs associated with decreased on-site power production from combined heat and power plants and/or purchased electricity, as well as the avoided

DocuSign Envelope ID: 5DB6E2D2-8865-4C2F-9B02-F38647AC4CB4

cost of carbon.

3. Off-campus Clean Electricity

   a. Clean electricity is defined as having a residual greenhouse gas emission factor that is less than 150 lbs. CO2/MWh.

   b. Clean electricity will be procured through the following methods and reported on annually:

      iii. A location may opt-in to a utility-provided green power program for its purchased electricity that meets the definition of clean electricity specified in V.B.3.a.

      iv. The UC Clean Power Program, which will procure and supply to participating campuses 100% clean electricity.

      v. Those locations without access to a green power program may purchase Renewable Energy Credits (REC) to offset purchased electricity. To be counted, such RECs will be transferred to UC or retired on behalf of UC.

4. Where feasible, the University will seek to benefit from the economies of scale and reduce risk by developing a portfolio for systemwide clean energy procurement contracts from which locations may benefit.

5. On-campus Combustion

   a. The University will develop and procure biogas supplies under the direction of the Energy Services Unit Governing Board (The Governing Board). The Governing Board will establish acceptable pricing for biogas projects and determine how the biogas will be allocated to each location. Locations may also implement local projects to directly transport biogas to the location.

C. Climate Protection

1. Each campus will maintain individual membership with The Climate Registry (TCR).[5] Campuses will include their health locations in their membership.

2. Each campus will complete a Greenhouse Gas (GHG) emissions inventory annually. Campuses will include their health locations in their inventories.

3. To comply with TCR and the Second Nature Carbon Commitment requirements,[6] inventories should contain emissions of the six Kyoto greenhouse gases from scope 1 and 2 emission sources outlined in the TCR General Reporting Protocol; and scope 3 emissions sources outlined by the Second Nature Carbon Commitment's Implementation Guide. All UC campuses will report their updated emissions inventories through the Second Nature Carbon Commitment online reporting tool at least biennially. Campuses must verify all emissions inventories through TCR. Campuses may either pursue verification annually (for the previous year's emissions inventory) or biennially

---

[5] The Climate Registry is a nonprofit collaboration among North American states, provinces, territories and Native Sovereign Nations that sets consistent and transparent standards to calculate, verify and publicly report greenhouse gas emissions into a single registry.

[6] The Second Nature Carbon Commitment requirements are outlined at Second Nature: The Presidents' Climate Leadership Commitments.

DocuSign Envelope ID: 5DB6E2D2-8865-4C2F-9B02-F38647AC4CB4

(for the emissions inventories from the previous two years).

4. Campuses subject to the United States Environmental Protection Agency (USEPA) Greenhouse Gas Reporting Program, California Air Resources Board (CARB) Mandatory Greenhouse Gas Emissions Reporting, or participation in the CARB Cap- and-Trade Program will complete the relevant emissions inventories outlined in the USEPA and CARB reporting protocols.

5. Each campus will regularly update its climate action plan for (a) maintaining GHG emissions at or below 1990 levels ; (b) achieving climate neutrality for scope 1 and 2 sources by calendar year 2025 (annual 2025 emissions reported in 2026); (c) achieving climate neutrality for the Second Nature Carbon Commitment-specified scope 3 sources (as defined by Second Nature's Carbon Commitment) for calendar year 2050 (annual 2050 emissions reported in 2051); and, (d) climate action planning will integrate environmental justice, adaptation, and resilience. This will include an annual review and update, if needed, of the GHG reduction strategies reported by the campus to the UC Office of the President (UCOP). Campuses will include their health locations in the action plan.

6. Each campus will complete an assessment of Scope 1 emissions from natural gas combustion by 2035 or at the date when that location's combined heat & power plant (or any other major fossil fuel-using campus infrastructure) is planned for capital renewal or major repair, whichever occurs first. The assessment should determine the best pathway, at that point, to decarbonize 80% of scope 1 emissions through means other than offsets. A de-carbonization assessment should evaluate, but is not limited to, (1) progress toward de-carbonization of piped gas, (2) the feasibility of installing on-site carbon capture, (3) electrification of carbon-emitting plant equipment, (4) hydrogen or synthetic methane injection, (5) emergent technologies, and (6) energy efficiency directed at Scope 1 footprint reductions. The assessment should be provided to campus leadership and inform each campus's Climate Action Plan.

7. The Climate Change Working Group (CCWG), under the UC Sustainability Steering Committee and represented on the President's Global Climate Leadership Council, will monitor progress toward reaching the stated goals for GHG reduction and will evaluate suggestions for strategies and programs to reach these goals.

8. The CCWG will develop protocols for growth adjustment, data normalization, and accurate reporting procedures, as required.

9. The University will use only high-quality carbon offsets to meet its climate protection goals beyond its requirements under California's cap-and-trade program. High-quality offsets represent real, additional, quantifiable, durable, and enforceable emissions reduction or carbon removal that have undergone third-party verification.[7]

    a. For the purposes of this section, offsets are considered:

        i. Additional if the credited reductions would not have occurred were it not for the offset program or the University's climate protection policy. Additionality can be

[7] Third-Party Verification will involve an audit of offset project eligibility or claimed reductions or removals against an approved methodology by an independent party.

DocuSign Envelope ID: 5DB6E2D2-8865-4C2F-9B02-F38647AC4CB4

assessed for an individual project or for a project type.

ii. Durable if there is a very high likelihood that they will remain out of the atmosphere for 40 years on-site or through commitments to replace credits.

iii. Enforceable if the University is able to reasonably ensure that its quality standards are met.

b. The University recognizes that not all offset credits available for purchase from projects registered in the major offset registries represent high-quality emissions reductions.

c. The University will evaluate the quality of each offset project it uses, involving a peer review process overseen by the Carbon Abatement Technical Committee (CATC). The CATC will be made up of at least one representative from each University of California campus, LBNL, Office of the President, and at least one student and one faculty member representative from the University. This review will include evaluating individual projects, or types of projects, against the University's offset quality criteria by appropriate experts. Peer review is in addition to third-party verification.

d. Credits are considered to be real if the quantity of credits generated and used by a project, or a project type, does not exceed conservative estimates of the actual effect of the project, or the set of projects of the project type, on emissions. When there is uncertainty in emissions reduction/removal estimates, estimates are conservative when they are more likely to under-represent than to over-represent actual emissions reductions/removals achieved. Evaluations will take into account the following factors as detailed in the UC Offset Procurement Guidelines: project additionality, conservativeness of methods used to estimate emission reductions, including the baseline, and effects outside of project boundaries such as through leakage.[8]

e. The results of these evaluations, including quantitative assessments of credit quality and justifications for the assumptions and determinations made, will be released publicly for all offset projects or project types the University uses to meet its climate targets.

f. The CATC will develop and maintain additional criteria, guidelines, and procedures for evaluating offset projects against the University's quality and mission criteria in the UC Offset Procurement Guidelines to be published in 2022.

g. The University recognizes the quality and mission benefits of implementing its own offset projects. UC-initiated offset projects give the University greater knowledge about the project with which it can ensure the projects' additionality and have confidence in the emission reduction estimates. UC-initiated offsets can also support the University's mission by researching, testing, and refining climate mitigation solutions and supporting student education which can have climate mitigation

---

[8] An offset project results in leakage when it reduces an activity, in turn causing that activity, and the associated emissions, to shift location to somewhere outside of the offset project boundaries. For example, a project that increases forest carbon by reducing timber harvesting can result in increased harvesting on other forestlands to meet timber demand.

benefits far beyond the reduction from the credited offset project. The University system and its individual campuses and units will prioritize offset projects with active University involvement.

h. Decisions affecting offset procurement will be made in the context of the location's climate action plan while following the offset requirements set forth in this Policy.

D. Sustainable Transportation

1. The Sustainable Transportation Working Group, with input from the Climate Change Working Group, will develop normalized data reporting protocols to track progress on the implementation of sustainable transportation programs. Annually, each location will collect and report:

   a. Fleet efficiency metrics: fleet fuel consumption, total vehicle inventory, and total number and percent of new ZEV fleet acquisitions.

   b. Commute data: employee and campus-wide mode split, including telecommute and compressed week, average vehicle ridership (AVR), and percent of commuter alternative fuel vehicles, including ZEVs.

      i. Average vehicle ridership is calculated by dividing all person trip arrivals by private vehicle trips, with adjustments for telecommuting, compressed work weeks, and zero-emission vehicles (based on the South Coast Air Quality Management District's methodology).

   c. Number and type of alternative fuel infrastructure (e.g., electric vehicle charging stations, natural gas, etc.).

2. Due to the unique characteristics of each campus' fleet management protocols, each location will:

   a. Develop a Fleet Sustainability Implementation Plan by January 1, 2022, to document the infrastructure and financial needs to implement a low-carbon fleet program and lower campus fleet carbon emissions through 2025.

   b. Implement practical measures to improve fleet emissions, including, but not limited to, managing vehicle fleet size, eliminating non-essential vehicles, purchasing the cleanest and most efficient vehicles and fuels, and investing in clean bus operations.

   c. Establish a local process for centralized review and approval of vehicle acquisitions to ensure that those acquisitions comply with this Policy, that non-compliant acquisitions are operationally and financially justified, and that locations take advantage of opportunities to improve fleet utilization and efficiency.

3. Explore partnerships with local agencies, including the Department of Energy's Clean Cities program, on opportunities to improve sustainable transportation access to and around University facilities in addition to developing its own transportation programs.

4. Each location will implement parking management and pricing strategies to support emissions reduction, trip reduction, and sustainable transportation goals, including variable pricing and unbundling parking and housing costs.

5. The University will pursue strategic programs and data collection to offset greenhouse gas emissions related to commutes and business-related campus air travel. The

DocuSign Envelope ID: 5DB6E2D2-8865-4C2F-9B02-F38647AC4CB4

Sustainable Transportation and Climate Change Working Groups will set an interim emissions reduction target for transportation-related scope 3 emissions.

6. The Sustainable Transportation Working Group will support central and local Human Resource Offices, and other key stakeholders, in developing systemwide best practices guidance on telecommuting, flexible work schedules, and other alternative work arrangements. Any recommendations should take into consideration issues surrounding costs, savings, challenges, and equity.
7. This Policy will be consulted for all new campus development – including acquisitions and leases – to evaluate how the development or acquisition would meet the transportation policies and goals of the campus and University.
8. The Sustainable Transportation Working Group will regularly update the systemwide best practices guide for implementing this Policy and take steps to implement the best practices identified throughout the UC system. Mechanisms for reducing transportation emissions include, but are not limited to:
    a. Constructing additional on-campus housing (e.g., student housing and temporary housing for new faculty)
    b. Expanding transportation demand management (TDM) programs: car share, carpool/rideshare, vanpool, shuttles, transit, bicycle circulation system, pedestrian circulation system, emergency rides home, parking management and pricing, employee service, retail amenities, etc.
    c. Expanding intra-campus transportation programs such as shuttles, car share, bike share, bicycle, pedestrian infrastructure, etc.
    d. Encouraging flexible work schedules and/or telecommuting programs to provide alternative commute flexibility and options in accordance with local practices.
    e. Replacing fleet vehicles with newer, more fuel-efficient vehicles when ZEV are not available
    f. Rightsizing fleets (determining the appropriate fleet size, revising business practices to reduce the need for travel)
    g. Reducing overall fleet miles traveled
    h. Increasing use of fuels with lower GHG emissions
    i. Installation of telematics and GPS to measure and help reduce fuel consumption by monitoring and reducing excessive idling and speeding.

E. Sustainable Building and Laboratory Operations for Campuses

1. The University will incorporate the Sustainable Building and Laboratory Operations policy requirements into existing facilities-related training programs, with the aim of promoting and maintaining the goals of the Policy.
2. The University will work closely with the USGBC to address the needs and concerns of campuses in the further development of USGBC programs, including the LEED-O+M rating system and the USGBC's "Application Guide for Multiple Buildings and On- Campus Buildings."

3. Campuses will use the LEED-O+M certification process to advance the University's educational and research mission by using the buildings as living, learning laboratories.
4. Campuses will assess at least three new research groups through their Green Lab Assessment Program.
5. Campuses will maintain a UC Green Laboratories Action Plan to determine strengths and areas for improvement within the operations of research laboratories with respect to sustainability and carbon neutrality. A standard template for this with required sections will be maintained and updated by the Sustainable Building and Laboratory Operations Working Group, and this plan will be updated every four years (2018, 2022, 2026 and so on).
6. Each campus will report annually on their Green Labs program progress, including the number of researchers directly and indirectly engaged by the program each year.

F. Zero Waste

1. The University will voluntarily comply with Chapter 18.5, the "State Agency Integrated Waste Management Plan," in California Public Resources Code Section 40196.3.
2. Waste reduction and recycling will be prioritized in seeking LEED credits for LEED- BD+C, LEED-ID+C, and LEED-O+M projects.
3. By the end of 2018, locations other than health locations will submit new waste management plans, including planned waste reduction strategies. Plans will include campus and regional waste management practices and options, evaluate progress towards Policy goals, and determine the associated costs of achieving Policy goals. Waste management plans will be updated and submitted to the Office of the President's Associate Vice President of Capital Programs, Energy and Sustainability every five years.
   a. The 2023 updates to locations' waste management plans will identify the next steps to take (including costs, responsible parties, etc.) towards eliminating non-essential single-use plastics by 2030 and assess other opportunities for eliminating other single-use products. The findings of these assessments will be used to recommend changes and additions to section III.F.4. of this Policy no later than July 1, 2024.
4. In line with the objective to minimize the use of single-use products (Section III.F.4), all locations will,
   a. Create a local implementation procedure that includes the delineation of an exception/exemption protocol (i.e., identifying campus authority, implementation authority, etc.) for cases where reasonable alternatives to plastic do not exist. Key stakeholders could include sustainability, dining, athletics, event services, and other departments that operate foodservice facilities. Local procedures may consider allowing plastic water bottles for emergency services, emergency water storage, and at events where alternatives are not practically available.
   b. Work to identify and reduce single-use plastics that are not identified in section III.F.4.
   c. Recognize that accessibility for and inclusion of the disability community is a priority and integrate best practices into their local implementation procedures to ensure this

DocuSign Envelope ID: 5DB6E2D2-8865-4C2F-9B02-F38647AC4CB4

Policy and its implementation do not create barriers to access or an unwelcoming environment. This includes providing reasonable alternatives to single-use plastic products. If reasonable alternatives are not available, a small stock of single-use plastics (including, but not limited to, plastic straws) should be maintained and made readily available for individuals who need them either at the point of service/cashier; or upon request at dine-in facilities.

5. Exceptions will be considered for entities that represent less than 1% of the overall campus solid waste tonnage.
6. Reduction, reuse, recycling and composting are the primary methods to be counted toward the municipal solid waste diversion from landfill goals. The goal is to strive for the highest form of resource recovery methods and the best use of the materials. The hierarchy for resource recovery is as follows:
    a. Source reduction: The reduction of waste is the highest form of resource recovery as it eliminates the products from being manufactured or transported in the first place.
    b. Reuse: Reuse materials in their original form (e.g., use lumber for lumber, mugs instead of single-use cups, reuse course readers in subsequent classes. These methods maintain the embodied energy in each material.)
    c. Composting and recycling: Composting is the recycling of organics such as animal waste, bedding, green waste, and food waste into compost and mulch. Recycling refers to the conversion of waste into basic materials so they can be made back into new products.
    d. The methods of reusing and recycling waste vary and will evolve over time as technologies improve. The Zero Waste Working Group – comprising waste and recycling professionals from each location – will continue to evaluate recycling methods and recommend their appropriateness for counting toward diversion goals.
7. Waste Reduction: For the purposes of measuring waste reduction, reporting will be in waste generated per capita per day. Waste generated includes municipal solid waste that goes to landfills and all waste that is diverted through recycling, organics or conversion technologies. Not included in waste reduction calculations are:
    a. Waste generated as part of major construction and demolition projects;
    b. Organic waste generated due to landscape management;
    c. Agricultural and animal-related waste.
8. Per capita metrics will be understood in the context of business operations and activities:
    a. Campuses will use Weighted Campus User
    b. LBNL will use Full-Time Equivalent

    Other locations should use the per capita metric that best supports their business operations.
9. Locations other than health locations will strive to achieve 90% diversion of municipal solid waste as soon as feasible through steps that include but are not limited to

DocuSign Envelope ID: 5DB6E2D2-8865-4C2F-9B02-F38647AC4CB4

partnering with local waste haulers to maximize diversion opportunities available and actively engaging with their local campus users to improve source separation. These locations will outline their strategy for maximizing diversion in their waste management plans and updates. Every year, locations will report to UCOP on their progress and next steps towards meeting this target and identify common barriers and opportunities.

10. The Zero Waste Working Group will coordinate the development of a systemwide best practices guide to outlining methods for quantifying waste generation and diversion at University locations. This guide will include recommendations on boundaries, calculation methodologies, contamination rates, tools, best practices for waste reduction and diversion, etc.

11. Where significant data methodology errors are found in benchmark years, an appropriate alternative methodology will be determined by agreement with UCOP and the Zero Waste Working Group.

12. Reporting of solid waste and recycling data will follow ULs Environmental Claim Validation Procedure for Zero Waste to Landfill (UL2799: 2017-03-22: 3rd Edition) and should be applied in principle to future standards/ editions. Where there are discrepancies between UC policy definitions and goals and UL2799 and subsequent editions, the Policy language will apply.

13. Campuses will be able to meet up to 10% of their diversion targets through combustion until the end of FY2021/22, after which the UC will no longer accept combustion as a form of diversion. No campus will increase the percentage of combustion reported as diversion from reported FY2015/16 levels. Up to 10% of total waste generated per campus may be disposed of through allowable thermal residual conversion after FY2021/22. To count, (non-combustion) waste converted through thermal processes must include an integrated materials recovery facility (MRF) or equivalent sorting system to recover recyclables and compostable material prior to conversion. The total value of converted materials counted as diversion from landfill is not to exceed 10%.

    a. Consistent with CalRecycle and the Southern California Conversion Technology Project, Allowable Thermal Residual Conversion includes: thermal, chemical, mechanical, and/or biological processes capable of converting post-recycled residual solid waste into useful products and chemicals, green fuels like ethanol and biodiesel, and clean, renewable energy. It does not include combustion. Examples include the transformation of post-recycled residual materials into usable heat or electricity through gasification, pyrolysis, distillation, or biological conversion other than composting. To count as allowable residual conversion, the process must include an integrated materials recovery facility (MRF) or equivalent sorting system to recover recyclables and compostable material prior to conversion. Materials that are otherwise landfilled or incinerated, including biomass conversion operations that exclusively incinerate organic materials, landfill-gas-to-energy (LFGTE) facilities, and other facilities that do not employ integrated materials recovery or equivalent sorting and recovery systems may not be considered as converted residual waste.

G. Sustainable Procurement

1. This section V.G. will be applied within the constraints of research needs and budgetary requirements and in compliance with applicable rules, regulations and laws.

DocuSign Envelope ID: 5DB6E2D2-8865-4C2F-9B02-F38647AC4CB4

2. The University will work to remove harmful chemicals from products brought onto campus by increasing the purchase of products and materials that disclose known hazards (e.g., in compliance with the requirements of LEED BD+C v4 "Building product disclosure and optimization - material ingredients" - or updated equivalent) and choosing products with reduced concentrations of chemical contaminants that can damage air quality, human health, productivity, and the environment.

3. The University will require suppliers to clearly identify products with UC-recognized certifications, as defined by the Guidelines, in both hosted and punch out catalog e- procurement environments.

    a. Commodity/Contract Managers will work with all contracted suppliers to ensure that contract items that meet the UC criteria for Green and Economically and Socially Responsible (EaSR) Spend as outlined in the Guidelines will be prioritized in all product searches.

    b. Unless locations request otherwise, products that do not meet the University's minimum criteria requirements will be blocked in all hosted catalogs and punch out catalogs upon contract award.

4. The University will require all strategically sourced suppliers to report annually on their sustainable business operations and quarterly on the University's sustainable purchasing activity. Quarterly spend reports will be collected by the appropriate University of California Procurement Services department. Quarterly spend reports must be filterable, include all products and services purchased, use an Excel-compatible software, include information on a single sheet, and include the following fields:

    - Campus
    - Department and/or delivery location
    - SKU and/or manufacturer number
    - Item description
    - 8-digit UNSPSC code
    - Product category/Title of UNSPSC code
    - Quantity
    - Unit of measure
    - Price
    - Third-party sustainability attribute or certification as recognized in the Guidelines

5. Locations, not including health locations or the Lawrence Berkeley National Lab, will report annually to the UC Office of the President (UCOP) their percent Preferred Level Green Spend and EaSR Spend for product and service categories defined in the Guidelines. For the first two years of reporting, reports on Preferred Level Green Spend will include, at minimum, a location's share of products purchased from systemwide strategically sourced suppliers, with reports to be provided by the suppliers to UCOP and locations. EaSR Spend reporting will be compiled at the campus level, with the

DocuSign Envelope ID: 5DB6E2D2-8865-4C2F-9B02-F38647AC4CB4

support of UCOP. Reports will be reviewed by each location for accuracy and signed by the location's Chief Procurement Officer, with reporting due 60 days after fiscal close.
Reporting procedures will be reviewed after two years of reporting under this Policy.

6. The University Standards for all packaging materials will be outlined in all solicitations. Suppliers will be required to demonstrate how their standards and practices for packaging materials meet the UC Standards.
   a. Additional consideration in bid evaluations will be given to suppliers who meet more than one criteria listed in 8 (a) - (e) for packaging, and with preference given to bids meeting 8 (b).
7. In accordance with section III.F.3., the University has disallowed the use of packaging foam after 2020. For implementation procedures, reference the University of California Sustainable Procurement Guidelines.
8. The University requires that all packaging be compliant with the Toxics in Packaging Prevention Act (AB 455) as to be free of any intentionally introduced lead, cadmium, mercury or hexavalent chromium, and containing no incidental concentrations of these regulated metals greater than 100 parts per million (ppm) by weight. In addition, the University requires that all packaging meet at least one of the criteria listed below:
   a. Uses bulk packaging;
   b. Uses reusable packaging (e.g., totes reused by delivery service for next delivery);
   c. Uses innovative packaging that reduces the weight of packaging, reduces packaging waste, or utilizes packaging that is a component of the product;
   d. Maximizes recycled content and/or meets or exceeds the minimum post-consumer content level for packaging in the U.S. Environmental Protection Agency Comprehensive Procurement Guidelines;
   e. Uses locally recyclable or certified compostable material.
9. Suppliers, when interacting with the University, will be prohibited from providing hard copies of presentations or other materials. Suppliers will be required to present all information in an electronic format that is easily transferable to University staff, who may choose to print their own copies in accordance with UC Policy if necessary. Materials may be provided if specifically required or requested by a UC representative.
10. All recyclers of the University's electronic equipment must be e-Steward certified by the Basel Action Network (BAN). In cases where the University has established take-back programs with a manufacturer, the University will encourage the manufacturer to become a BAN-certified e-Steward Enterprise (e-Stewards for Enterprises).
11. The responsible authority for granting exceptions to items III.G.5.a. and V.G.7. in the Sustainable Procurement section of this Policy will be the Chief Procurement Officer for a non-UC Health systemwide or Office of the President contract; the AVP, UC Health Procurement for a UC Health Systemwide contract; and otherwise by the Procurement/Supply Chain Director of the campus, medical center, or Laboratory.

H. Sustainable Foodservices

1. Campus and health location foodservice operations subject to this Policy will include

DocuSign Envelope ID: 5DB6E2D2-8865-4C2F-9B02-F38647AC4CB4

self-operated and contract-operated foodservices, as well as foodservices in leased locations.

2. Sustainable food is defined as food and beverage purchases that meet AASHE STARS' "sustainably and ethically produced" food for campuses and Practice Greenhealth's "sustainable food" for health locations, as outlined below:

   a. AASHE STARS 2.2 Sustainably and Ethically Produced for campuses;

   b. Practice Greenhealth Healthier Food Purchasing Standards for health locations.

3. Plant-based foods as defined by the Culinary Institute of America's Menus of Change program includes fruits and vegetables (produce); whole grains; beans; other legumes (pulses), and soy foods; nuts and seeds; plant oils; herbs and spices; simple combinations of these foods and their derivatives, and vegetarian/ vegan alternatives to meat and dairy.

   a. AASHE STARS provides additional guidance on processed food items.

   b. Animal products (i.e., meat, poultry, fish, seafood, eggs, and dairy) and their derivatives, drinking water, and most ultra-processed foods do NOT qualify as plant- based foods. Examples of ultra-processed foods include sweet or savory packaged snacks; chocolate and candies (confectionary); mass-produced packaged breads and buns; cookies (biscuits), pastries, cakes, and cake mixes; instant sauces; many ready to heat products, including pre-prepared pies and pasta and pizza dishes; powdered and packaged 'instant' soups, noodles and desserts; carbonated drinks; 'energy' drinks; 'fruit' drinks; and distilled alcoholic beverages such as whiskey, gin, rum, and vodka.

4. All foodservice operations should track and report annually the percentage of total annual food budget spent on sustainable food and plant-based products.

5. Each campus and health location procurement department will integrate sustainability into competitive solicitations. Procurement departments will allocate a minimum of 15% of the points utilized in solicitation evaluations to sustainability criteria. Additional guidelines for procurement are listed in III G and the UC Sustainable Procurement Guidelines.

6. The University prioritizes waste reduction in the following order: Reduce, reuse, and then recycle and compost. Campuses, health locations, and leased foodservice operations are encouraged to utilize compostable foodservice containers and packages that have recycled and/or sustainably harvested content wherever possible. Guidelines for compostable foodware are listed in the UC Sustainable Procurement Guidelines.

7. Each campus and health location is encouraged to maintain accessibility and affordability for all students, staff, and patrons. Campuses are encouraged to explore food recovery programs that can support campus basic needs programs.

I. Sustainable Water Systems

1. Reporting Methods

   a. Explicitly identify the geographic and operational areas comprising the scope of location water usage (e.g., the campus as defined by its Long Range Development

DocuSign Envelope ID: 5DB6E2D2-8865-4C2F-9B02-F38647AC4CB4

Plan boundary, excluding third-party operated facilities).

b. Locations with health locations may choose to report health locations data and progress toward the target separately from the main campus.

c. All locations will report water usage in a tabular format using the following methods:

i. Measure per capita water consumption by Weighted Campus User (WCU) for main campuses and Adjusted Patient Day (APD) for health locations. If necessary, WCU and APD may be combined using the following calculation: [(APD/360) * 1.5] + WCU;

ii. Potable water usage for a baseline period that is three consecutive fiscal years, including FY 2005/06, 2006/07, and FY 2007/08:

- Total location potable water usage, in gallons, for each of the three years comprising the baseline period,
- WCU, or APD, for each of the three years comprising the baseline period,
- Baseline Potable Water Usage: calculate the baseline metric as follows: Step 1: Divide each year's total water use in gallons by that years' WCU or APD population. Step 2: Average the three gallons/population calculations to derive the Baseline Potable Water Usage for the location,
- Multiply the Baseline Potable Water Usage figure by 0.64 to derive the location's 2025 Potable Water Usage Target, and
- Unless impracticable, provide average gallons of potable water usage per baseline year per gross square foot of location built space for which potable water consumption is being reported

iii. Potable water usage for the most recent fiscal year.

- If using only the most recent fiscal year, and not an average, list in the table the following:
  - Total location potable water usage, in gallons, for the most recent fiscal year,
  - WCU or APD for the most recent fiscal year,
  - Divide the gallons by the WCU or APD to derive the Current Potable Water Usage, and
- If feasible, provide average gallons of potable water usage per gross square feet for either the three most current fiscal years, if that is the method adopted, or for the single most current fiscal year, using the methodology described above.

iv. If data is available, total location non-potable water usage, in gallons, for the most recent fiscal year.

v. If data is available, report or estimate water usage in the following use categories at a minimum: buildings, landscape, and central plant including cooling towers, identifying the quantities of potable and non-potable used for these purposes.

2. Reporting Schedule

   a. Each location prepared a Water Action Plan as specified below and submitted it to the Office of the President by December 2013.

   b. Beginning the following year, each location will provide an annual progress report on implementing its Water Action Plan to include progress on its water usage reduction.

3. Water Action Plans

   a. Each Water Action Plan will include:

      i. Water usage and reduction strategies addressing major categories of usage such as irrigation and landscaping, potable water, non-potable water, industrial water, sterilized water, reclaimed water, wastewater, and any other water systems;

      ii. Stormwater management, including stormwater capture and reuse (or reference to the campus' separate stormwater management plan, if one exists);

      iii. Suggestions for implementation of innovative water-efficient technologies as part of capital projects and renovations (e.g., installation of WaterSense certified fixtures and appliances, greywater reuse, rainwater harvesting, and watershed restoration); and

      iv. Education and outreach on water conservation.

   b. Each Water Action Plan, and the water conservation and water efficiency strategies they contain, will also take into account relevant regional conditions and regulatory requirements, will recognize historical progress, and will acknowledge current location best practices implemented.

J. Sustainability at UC Health

1. The UC Health Sustainability Working Group, with input from relevant working groups for each subject area, will develop normalized data reporting protocols to track the implementation of sustainability programs at health locations. Annually, the UC Health Sustainability Working Group will report to the University of California Health Center Chief Operating Officer Group and the University of California Sustainability Steering Committee.

2. Health locations will participate in Practice Greenhealth's reporting program and report at a minimum metrics for energy, carbon, water, and waste. To meet the reporting requirements, reporting to Practice Greenhealth will reflect UC Health location boundaries and will use either adjusted patient encounters or adjusted patient days as appropriate to reflect non-licensed patient encounters. Reporting to Practice Greenhealth will be based on the most recently completed fiscal year.

3. Health locations may discretionarily submit additional facility-specific applications to Practice Greenhealth for award consideration in addition to a total site/campus application. The stated goal of achieving Practice Greenhealth Partner for Change Awards may be at the campus or facility level.

K. General Sustainability Performance Assessment

1. The rating must be for a current certified STARS report and under the current STARS

DocuSign Envelope ID: 5DB6E2D2-8865-4C2F-9B02-F38647AC4CB4

point allocations.

L. Health and Well-Being

1. The Healthy Campus Network will build a systemwide working group that will work closely with campuses, health locations and community stakeholders to build out and coordinate implementation of this section of the Policy.

VI. RELATED INFORMATION

AASHE STARS 2.2 Sustainably and Ethically Produced (Food and Beverage Purchasing) AASHE STARS guidance on processed food items

BFB-BUS-43 Purchases of Goods and Services; Supply Chain Management

BFB-BUS-38: Disposition of Excess Property and Transfer of University-Owned Property California Air Resources Board LCFS Pathway Certified Carbon Intensities

California Building Code, Title 24

California Energy Commission's Renewables Portfolio Standard Guidebook e-Stewards for Enterprise

Facilities Inventory Guide

Federal Trade Commission's (FTC) Green Guides

Practice Greenhealth Healthier Food Purchasing Standards for health locations Public Contract Code: Materials, Goods, and Services, Section 10507.8

Public Contract Code: Construction State Administrative Manual

The Climate Registry

Trademark Licensing Code of Conduct

UC Annual Report on Sustainable Practices

UC Flexible Work Arrangements and Telecommuting Website UC Sustainability Website

UC Sustainable Procurement Guidelines UC Sustainable Procurement Website

UC 2016 Whole-Building Quantitative Energy Performance Targets (2020 update)UL 2799 Environmental Claim Validation Procedure for Zero Waste to Landfill

U.S. Environmental Protection Agency Comprehensive Procurement Guidelines

VII. FREQUENTLY ASKED QUESTIONS

Not applicable.

DocuSign Envelope ID: 5DB6E2D2-8865-4C2F-9B02-F38647AC4CB4

VIII. REVISION HISTORY

March 10, 2022: Policy revised to update the following sections with new goals, procedures, and clarifications: Green Building Design, Climate Protection, Sustainable Transportation, Sustainable Water Systems, and Sustainability at UC Health. Added a Health and Well-Being Policy section. Made minor clarifications to water and procurement sections.

Updated the Green Building Design section to reference an updated list of whole building performance targets that include 100% Lab Space and include reporting on the energy efficiency policy requirement for new buildings.

Included new provisions establishing criteria for the purchase of carbon offsets to the Climate Protection sections, added a reference to climate justice in campus's Climate Action Plans, and clarified that GHG reductions should be maintained after the 2020 target date.

Replaced the fleet targets in the Sustainable Transportation section with ones that better reflect State policy and technological advances. Incorporated telecommuting into the Sustainable Transportation goals.

Updated the Sustainable Water Systems section to make it easier to read and removed expired dates and details that are already regulatory requirements.

Revised the water and waste goals for health locations so that the same targets are now applicable to each health location.

July 2020: Policy revised to update the following sections with new goals, procedures, and clarifications: clean energy, climate protection, sustainable building and laboratory operations for campuses, sustainable foodservice, zero waste, and UC Health. Policy expanded to add a section for general sustainability performance assessment. The following provides more details on the updates:

Added a new provision to the Climate Protection section to require that campuses formally assess options for reducing emissions from combined heat and power plants before capital renewal or major repairs.

Updated the Zero Waste section to integrate the waste diversion and minimization targets into a new zero waste goal and add a new Policy provision to begin phasing out single-use plastic bags and foodware items.

Replaced the 2020 goal in the Sustainable Food Services section, which has already been met, with a new 2030 goal that aligns with the Association for the Advancement of Sustainability in Higher Education's (AASHE) Sustainability Tracking, Assessment and Rating System (STARS) and Practice Greenhealth's requirements.

Added a General Sustainability Performance Assessment section that codifies participation by all undergraduate campuses in the AASHE STARS rating system and achieving a gold rating by 2023.

Updated the UC Health Policy Section to include new waste and water targets for UCI Health and to reference existing green building and sustainable food requirements.

Made other small formatting and wording changes to improve the clarity and readability of the Policy and to clarify which Policy sections apply to the Lawrence Berkeley National Laboratory.

DocuSign Envelope ID: 5DB6E2D2-8865-4C2F-9B02-F38647AC4CB4

January 2019: Policy revised to clarify the following sections: climate protection, zero waste, and sustainable procurement.
August 2018: Policy expanded to include UC Health. Changed the name of the Environmental Preferable Purchasing section to Sustainable Procurement. Policy revised to update the following sections with new goals and clarifying language: definitions, green building design, clean energy, zero waste, and sustainable procurement.

June 2017: Policy remediated for accessibility according to Web Content Accessibility Guidelines (WCAG) 2.0 Policy revised to reflect the University Carbon Neutrality Initiative, adding definitions of green lab assessment programs, "research group" as defined by the Laboratory Hazard Assessment Tool (LHAT), and the inclusion of the UC Green Laboratories Action Plan. Changes were also made to the sections for Sustainable Building Operations for Campuses.

June 2016: Policy revised to update the following sections with new goals and clarifying language: definitions, green building design, sustainable transportation, and sustainable water systems.

June 2015: Policy revised to update the following sections: sustainable building operations, sustainable foodservices practices, green building design, and clean energy.
July 2011: Policy revised to update the following sections: green building design, climate protection practices, sustainable operations, environmentally preferable purchasing, and sustainable foodservice practices.

September 2009: Policy expanded to include sustainable foodservice

March 2007: Policy expanded to include sustainable operations, waste reduction, and environmentally preferable purchasing; renovations guidelines added to green building section, climate protection section refined

January 2006: Policy expanded to include transportation and climate protection

June 2004: President formally issued the "Presidential Policy on Green Building Design and Clean Energy Standards." This Policy was subsequently renamed the Policy on Sustainable Practices

July 2003: The Regents approved sustainability policy principles (UCOP Sustainability)

DocuSign Envelope ID: 5DB6E2D2-8865-4C2F-9B02-F38647AC4CB4

APPENDIX B
UC BERKELEY FOOD AND BEVERAGE CHOICES POLICY

University of California, Berkeley Policy Issued: January 1, 2018

Effective Date: November 1, 2020

Supersedes: January 1, 2019

Next Review Date: November 1, 2025

Name of Policy:

Responsible Executive: Vice Chancellor-Student Affairs

Responsible Office: University Health Services

Contact: Kim Guess, kguess@berkeley.edu; (510) 457-5578

2. POLICY STATEMENT

The University of California, Berkeley ("Berkeley") is strongly committed to supporting the health and well-being of all members of the campus community by ensuring that healthy food and beverage choices are available in the foodservice operations identified in this policy. This policy establishes nutrition standards for retail foodservice and markets, vending machines, Athletic concessions, dining commons, and University-sponsored meetings and events to ensure accessibility of whole and fresh foods, provide healthier alternatives to sugar-sweetened beverages, and engage positive communications promoting healthier choices.

3. SCOPE OF POLICY

This policy applies to everyone at UC Berkeley, including visitors, prospective students, and others, except as specifically noted.

4. WHY WE HAVE THIS POLICY

A nutrition policy on the Berkeley campus is one of the most effective environmental strategies to address obesity, prevent diabetes, and improve the diet and health outcomes of the campus population. Health education alone—in the absence of change to the food environment—does not produce lasting behavioral change. This policy will help to ensure that healthy food and beverage choices are available in retail foodservice operations and markets, vending machines, Athletic concessions, and University sponsored meetings and events.

The nutrition standards set forth in this document are not intended to restrict choices but are designed to make healthy choices more accessible, appealing, and affordable. They are modeled on:

- The 2015 Dietary Guidelines for Americans, which recommend a healthy eating pattern with limited added sugar and saturated fat, less salt, and more fruits, vegetables and whole grains.
- The Culinary Institute of America's Menus of Change.
- UC's Global Food Initiative goal on Food Policy and Public Impact.
- Let's Get Healthy California with the goal to make California the healthiest state in the nation.
- The Centers for Disease Control (CDC) Improving the Food Environment Through Nutrition Standards.
- The National Alliance for Nutrition & Activity (NANA) call to action for public universities.
- Evidence-based public health recommendations.

Berkeley aspires to create a campus that will have agreements in place with:

- Foodservice operators serving the University who have the ability to provide high- quality, affordable food and beverages that align with these Bear Minimum Food and Beverage Standards not later than 2020.
- Foodservice operators serving the University who have the ability to provide a diverse selection of healthy beverage options not later than 2022.

5. *PROCEDURES*

Foodservice operations required to comply with this policy include:

- University-operated retail foodservice operations on campus, including Cal Dining and Cal Athletic Concessions.
- University-operated dining commons, including Cal Dining locations that are off-campus
- University-operated retail markets and convenience shops on campus.
- Third-party tenants with new leases or lease renewals on campus.
- Food and beverage vending machines.

The campus will recommend (but not require) adherence to the standards set forth in this policy by foodservice operated on University-controlled property by off-campus third-party vendors/tenants under lease or contract agreements, including but not limited to coffee shops, restaurants, and Mobile Food Facilities (i.e. "food trucks"). The standards set forth in this policy are recommended (but not required) to be included in the process for RFP's, lease negotiations, and lease contracts for off campus locations. Some preference in the selection of foodservice vendors/tenants will be given (when permitted by other policy or law) to third-party vendors/tenants that commit to be bound by specific operational standards consistent with this policy.

DocuSign Envelope ID: 5DB6E2D2-8865-4C2F-9B02-F38647AC4CB4

Exemptions:

- Foodservice operations subject to existing contracts and/or exclusive agreements are exempt until such time as those contracts and/or agreements are renewed or terminated.

These nutrition standards may be included in food and beverage contracts, exclusive contracts, and/or agreements established with the University.

All foodservice operations must be in compliance with current foodservice laws and applicable University of California and Berkeley campus policies including, but not limited to:

- University of California Zero Waste Policy
- Campus Policy Governing the Promotion of Alcoholic Beverages and Tobacco Products on the Campus and at Campus Sponsored Events
- State and federal legislation on Menu Labeling and ban on trans fats:
  - FDA Vending Machine Labeling Requirements
  - H.R. 2017 Common Sense Nutrition Disclosure Act of 2015 (requires all restaurant chains with 20 or more locations to post calories on menus)
  - FDA ruling by 2018 intended to ban all trans fats in food

A. NUTRITION STANDARDS FOR FOOD, SNACK VENDING, AND BEVERAGES

Bear Minimum Nutrition Standards for Food

Healthy Approach to Variety and Choice:

- Nutritious, fresh, whole foods that are minimally processed.
- Options and ingredients from various cuisines reflective of Berkeley's cultural diversity.
- Fresh vegetables and fruit featured across meals and snacks; and available as sides.
- Whole grain options provided; 100% whole grains are recommended as the standard choice.
- Plant-based protein/entrée always available when meat entrée is offered.
- Lean meats that are minimally processed.
- Trans-fat free plant oils.

Quality and Quantity:

- Options of small/moderately-sized portions of baked items, pastries, and desserts that are ideally whole grain and/or lower sugar.
- Options of healthier breakfast fare.
- Combination meals paired with sides of salad greens/non-fried vegetables or fresh fruit (instead of fries or chips) if combination meals are provided.
- Combination meals with half portion entrée paired with sides of salad greens/non-fried vegetables or fresh fruit if combination meals are provided.
- Cooking techniques prioritize grilling, roasting, stir-fry, baking, and poaching, with minimal deep-frying used as a preparation method.

5.1 Bear Minimum Nutrition Standards for Food at Dining Commons

On a regular business day during the school year, all dining commons must abide by these standards, which elaborate on the Bear Minimum Nutrition Standards for Food, which must also be followed.

- Offer a salad bar at lunch and dinner
- Offer a minimum of 1 cooked vegetable option at breakfast/brunch and a minimum of 2 cooked vegetable options at lunch and dinner, not including potatoes
- Offer fruit at all meals
- Offer a minimum of 2 whole grain options at all meals
- Offer a minimum of 1 plant-based protein option at each meal
- Offer a minimum of 1 lean meat option at lunch and dinner when meat is offered
- Use only oils that are trans-fat-free
- Include options that are lower in added salt and sugar
- Pre-portioned baked goods and desserts should be no larger than 2-ounce servings
- Offer no more than 2 deep fried options per meal at each dining common

The above nutrition standards are not applicable on days during which regular business is interrupted for any reason or when a simplified menu is deemed necessary by Cal Dining management.

5.2 Bear Minimum Nutrition Standards for Food Vending Machines

Healthier food items (snacks, entrées, and the entrée in a packaged meal) must have a fruit, vegetable, protein (including nuts and seeds), or whole grain as its first ingredient.

A minimum of 50% of the food offered in vending machines must meet the following Bear Minimum Nutrition Standards for vending:

Healthier Snacks must meet these nutrition standards per package:

- Not more than 250 calories with at least one option of 100 calories or less.
- Not more than 35% of total calories from fat (and less than or equal to 10 grams of total fat).
- Not more than 10% of total calories from saturated fat (and less than or equal to 3 grams of saturated fats).
- No trans fats.
- Not more than 35% total weight from sugar (and less than or equal to 20 grams of sugar).
- Not more than 360 milligrams of sodium.

Exemptions:

The following are exempt from all requirements except the calorie and sodium requirements, as long as they do not contain added sweeteners or fat: Nuts, nut butters (such as peanut butter),

DocuSign Envelope ID: 5DB6E2D2-8865-4C2F-9B02-F38647AC4CB4

seeds, legumes (beans), eggs, cheese, and fruits/vegetables, and combinations of these items (e.g. a fruit and nut mix).

Healthier Entrees must meet these nutrition standards per package:

- No more than 400 calories
- No more than 35% of calories from fat and no more than 16 grams of fat
- No more than 10% calories from saturated fat and no more than 5 grams of saturated fat
- No trans fat
- No more than 600 milligrams of sodium
- No more than 15 grams of total sugar
- The following are exempt from all requirements: Fresh or non-processed entrees, including entrees that are cooked or prepared on campus or in a commissary kitchen.

Healthier Meals must meet these nutrition standards per package:

- No more than 700 calories
- No more than 35% of calories from fat and no more than 28 grams of fat
- No more than 10% calories from saturated fat and no more than 8 grams of saturated fat
- No trans fat
- No more than 1000 mg of sodium
- No more than 35 grams of sugar
- The following are exempt from all requirements: Fresh or non-processed meals, including meals that are cooked or prepared on campus or in a commissary kitchen.

5.3 Bear Minimum Nutrition Standards for Beverages

<u>Healthier Beverages - Retail:</u>

- Retail operations selling bottles, cans, and packaged beverages will stock shelf space with "Healthier Beverages"* at 70% or greater, and "Sugar-Sweetened and Less Healthy Beverages"* at 30% or less.
- Retail operations selling bottles, cans, and/or packaged beverages will offer a variety of sizes from the smallest sizes available (4-8 ounces) to a maximum size of 20 ounces for sugar sweetened beverages; preferably in aluminum or glass packaging in support of UC's systemwide sustainability goals.
- Retail foodservice operations selling beverages from soda fountain dispensers recommended to provide a maximum cup size of 21 ounces, and preferably 16 ounces.
- Energy drinks and energy-type beverage products with caffeine as an additive ingredient sold in bottles, cans, and/or other packaging are limited to less than or equal to 71 milligrams of caffeine per 12 fluid ounces.

For made-to-order coffee/tea drinks sold by foodservice providers under University partnership/agreement, the University will work with the selected coffee/tea providers to address the goal to reduce added sugar. <u>Healthier Beverages – Vending Machines:</u>

- Vending machines selling bottles, cans, and packaged beverages will stock machines and have visible selection buttons with "Healthier Beverages"* at 70% or greater, and "Sugar-Sweetened and Less Healthy Beverages"* at 30% or less.

- Caloric beverages which do not follow healthier beverage standards must not exceed 20 fluid ounces in size.
- Energy drinks and energy-type beverage products with caffeine as an additive ingredient sold in bottles, cans, and/or other packaging are limited to less than or equal to 71 milligrams of caffeine per 12 fluid ounces.

Water:

- Bottled, canned, or otherwise packaged water, including sparkling or flavored water, will be priced equal to, or preferably lower than, sugar-sweetened and artificially sweetened beverages on a cost-per-serving basis.

In dining commons, water, including sparkling and infused water, will be available in at least 2 locations that are visible and easily accessible.

Exemptions:

Cal Athletic Concessions 32-ounce Souvenir Cups sold at football, and men's/women's basketball games. This exemption will be reviewed annually with an aim to be responsive to market demands while working towards selling smaller, alternative sizes in compostable cups with or without artwork.

*See Glossary

Consultation: University Health Services nutrition staff will be available to consult with foodservice operations and vending partners on healthy menu development, item placement, healthy item identification strategies, and consumer outreach and education.

B. MARKETING AND ADVERTISING

Marketing of healthy food and beverage options must be consistent with the following Marketing and Advertising Standards. Marketing of corporate brands and logos by vendors under exclusive agreements with the University are allowed and it is recommended to promote the diversity of a company's portfolio of brands, particularly healthier brands.

5.4 Marketing and Advertising Standards

Marketing Healthier Products:

- Marketing, promotional campaigns, displays, and posters of specific food and beverages should prioritize healthier food and beverage products meeting the Bear Minimum Nutrition Standards.

DocuSign Envelope ID: 5DB6E2D2-8865-4C2F-9B02-F38647AC4CB4

Menu Labeling:

- Healthy food and beverages may be identified with a healthy icon in partnership with campus programs such as Eat Well Berkeley.
- Coffee/tea providers under University partnership/agreements post calories on the menu, and will work with the University to promote point-of-service information on added sugar content for coffee/tea beverages and a healthier beverage menu.
- Dining commons will provide nutrition facts and ingredient lists that are easily accessible, which can include being available electronically via an app or on Cal Dining's website.

Product placement:

- Healthy food and beverages will be placed at check-out, eye level, above-the-knee, and/or at the best-selling position within the foodservice operation, vending machines, refrigerated containers, food carts, and/or other food venues; less nutritious offerings will be placed in less prominent locations.

Pricing:

- Pricing should be set to encourage the sale of healthier food or beverages.
- Discounts and promotions should be offered for customers using reusable food or beverage containers subject to proper health and sanitation practices.
- Pricing discounts for sugar-sweetened beverages and less healthy beverages, including but not limited to free refills or buy-one-get-one-free promotions, are not allowed.

Consultation: University Health Services nutrition staff will be available to consult with foodservice operations and vending partners on healthy menu development, item placement, healthy item identification strategies, and consumer outreach and education.

C. MEETINGS AND EVENTS

When University funds are used to purchase food or beverages for University meetings and events:

- Members of the University community ordering or purchasing food and beverages should include nutritious and sustainable choices as outlined in the UC Berkeley Healthy Meeting and Event Guide and supported by UHS -- Be Well at Work Wellness Program.
- Water, preferably served in bulk to minimize use of single-use water bottles, must be offered as a choice when beverages are offered.

D. ACCOUNTABILITY AND PERFORMANCE STANDARDS

- Foodservice operators and third-party vendors/tenants will designate at least one member of their staff to serve as liaison to the University representative responsible for this policy.

- Foodservice operators and third-party vendors/tenants will receive on an annual cycle the Berkeley Menu Assessment to complete and return to University Health Services.
- All foodservice operations will be subject to random assessments by the University.

For purposes of ensuring accountability with these nutrition standards, Berkeley will commit to the following:

- Partner nutrition experts with the University's Beverage Alliance.
- Provide assistance to foodservice operators and vendors as needed on these nutrition and marketing standards.
- Perform random assessments of foodservice operations and vending machines with the opportunity to provide experiential learning projects for students in Public Health and Nutritional Sciences & Toxicology courses.

6. RESPONSIBILITIES

6.1 Everyone in the Campus Community:

- Collaborates on this policy across multiple disciplines. Members of the campus community, including visitors, are encouraged to provide feedback to foodservice operators about adherence to the policy.

6.2 University Business Partnerships and Services:

- Identifies and partners with providers and vendors.
- Facilitates business partnerships leading to marketing through multiple campus venues.

6.3 Business Contracts and Brand Protection (BCBP):

- Reviews contract terms and ensures they include applicable references to the standards outlined in this policy.
- Documents agreements with third-party vendors on University-controlled properties.
- Collaborates with campus partners, as well as internal and external tenants and third- party vendors, on implementation of this policy.

6.4 Supply Chain Management:

- Ensures contracts are awarded to companies providing food, vending, catering, and food/beverage marketing/advertising that comply with the standards outlined in this policy.

6.5 Real Estate Development and Portfolio:

- Ensures the Request for Proposal (RFP) process for foodservice providers includes the standards outlined in this policy.

6.6 Foodservice Operations Serving the Campus Community:

- Retail foodservice operations owned and managed by the University or located in University controlled property operated by a third-party vendor under lease or contract that target as primary customers University students, faculty and/or staff, have primary responsibility to adhere to the standards outlined in this policy.

6.7 ASUC Student Union:

- Assumes primary responsibility for the management and administration of snack and beverage vending machines located on non-Residential & Student Service Programs (RSSP) campus property.

6.8 RSSP Vending Management:

- Assumes primary responsibility for the management and administration of snack and beverage vending machines in residence halls.

6.9 University Health Services:

- Collaborates with campus foodservice operators on implementation of this policy.
- Partners with and provides consultation to campus departments with responsibilities related to this policy, as well as campus event planners and academic programs.
- Engages students in the planning

7. WEB SITE ADDRESS FOR THIS POLICY

http://campuspol.berkeley.edu/policies/foodbeverage.pdf

For policy implementation assistance, see *uhs.berkeley.edu/foodbeveragepolicy*

8. GLOSSARY

Campus Community: those enrolled at or employed by the University of California, Berkeley, as well as individuals engaged in activities sponsored by the campus or an organization affiliated with the campus. More broadly, the campus community is a group of people who have the campus in common and are socially interdependent, participate together in discussion and decision-making, and share certain practices.

Employee: for purposes of this policy, any faculty, staff, post-doctorate, graduate, or undergraduate student who performs work for the University of California and is paid for it by the University of California.

DocuSign Envelope ID: 5DB6E2D2-8865-4C2F-9B02-F38647AC4CB4

Entrée: the primary food in a meal which contains two or more of the following groups: meat or meat alternatives, grains or bread, vegetables, or fruit. Examples include sandwiches, burritos, pasta, and pizza.

Foodservice: Retail sale of ready-to-consume food and beverages.

8.1 Healthier Beverages defined for bottled/canned/packaged beverages:

- Water: unflavored with no added sweeteners (caloric or non-caloric); includes still, carbonated, or mineral.
- Low-calorie beverages: zero-calorie and low-calorie diet and other beverages at less than or equal to 40 calories per 8 fluid ounces.
- Juice: 100% fruit or vegetable juice or juice blends, with no added sweetener (caloric or noncaloric), carbonated or still; and vegetable juices less than or equal to 230 milligrams of sodium.
- Juice beverage: 100% fruit or vegetable juice or juice blends diluted with water, with no added sweetener (caloric or non-caloric), carbonated or still.
- Milk: nonfat or low fat (1%), unflavored, and unsweetened.
- Plant-derived or non-dairy milk: (i.e. soy, rice, almond), unsweetened, and Vitamin D and calcium fortified.
- Supplemental protein/nutrition beverages with no added caloric sweeteners (e.g., protein drinks, nutrition shakes).
- Teas/coffees, unsweetened with only naturally occurring caffeine.

Marketing: encompasses the vast array of strategies designed to successfully influence consumer purchasing behavior of food and beverages, including but not limited to product placement, signage, pricing, samples, giveaways, and marketing/advertising through many different media.

Meal: a complete offering intended for breakfast, lunch, or dinner that includes an entrée with additional components packaged together. A packaged meal typically consists of the main entree, and one or two sides, such as a vegetable or fruit and a snack or dessert.

8.2 Sugar-Sweetened and Less Healthy Beverages defined for bottled/canned/packaged beverages:

- Sodas, including all carbonated beverages, with added sugar and exceeding the calorie limit under Healthier Beverages.
- Fruit juice drinks or elixirs including all fruit drinks, fruit juices, and fruit nectars with added sugar.
- All other drinks with added sugar and exceeding the calorie limit under Healthier Beverages, including but not limited to sports drinks, energy drinks, sweetened coffee/tea drinks, protein drinks, sugar-sweetened and flavored teas, waters, coconut waters, rice drinks, bean beverages, sugar cane beverages, horchata, and nonalcoholic wine/malt beverages.

University-controlled property on campus and off campus: for purposes of this policy, the "campus" means central campus property bordered by the north side of Bancroft Way, the east side of Oxford Street, the south side of Hearst Avenue, and the west side of Gayley Road, and "off campus" means any foodservice operation located elsewhere.

Vending Machines: machines that dispense food or beverages when some form of payment is inserted. Berkeley snack and beverage vending machines are managed under exclusive contract, partnership, and/or agreement, and oversight management is provided by the Associated Students of the University of California (ASUC) and Residential & Student Service Programs (RSSP).

9. *Related Documents and Policies*

- State of California Retail Food Code
- University of California Sustainable Foodservices Policy
- Berkeley Campus Zero Waste Guidelines
- Campus Policy Governing the Promotion of Alcoholic Beverages and Tobacco Products on the Campus and at Campus Sponsored Events
- Berkeley Alcoholic Beverages Policy
- Alcoholic Beverages and Tobacco Products, Promotion Of
- UC Entertainment Policy
- Mobile Food Facilities Policy
- UC Healthy Vending Guidelines

References

- Menu of Change, Culinary Institute of America
- 2015 Dietary Guidelines for Americans
- H.R. 2017 Menu labeling
- Herrera, 18 scientists urge FDA action on Monster, other caffeinated energy drinks, March 19, 2013
- Partnership for a Healthier America (PHA) Healthier Campus Initiative
- The Federal Health and Sustainability Guidelines for Federal Concessions and Vending Operations, Health and Human Services, General Services Administration, Centers for Disease Control and Prevention, Publication date: April 2012
- The National Alliance for Nutrition and Activity (NANA) Model Beverage and Food Vending Machine Standards, Center for Science in the Public Interest, 2013.
- Recommendations for Healthier Beverages developed by Healthy Eating Research, a program of the Robert Wood Johnson Foundation, March 2013.
