# Supplementary material for: Pouring Rights Contracts Between Soda Companies and Public Universities: An Institutional Barrier to Sugar-Sweetened Beverage Reduction
Source: Int J Health Policy Manag. 2025 Nov 5;14:8879. doi: 10.34172/ijhpm.8879 (PMC12958230; doi:10.34172/ijhpm.8879)
Supplement: Supplementary file 2 — Wave 1 Interview Questions for University Managers Who Work in Departments of Student Health, Dining, Athletics, and Contracts at University of California and California State University Campuses. [file ijhpm-14-8879-s002.pdf]

Article title: Pouring Rights Contracts Between Soda Companies and Public Universities: An Institutional Barrier to Sugar-Sweetened Beverage Reduction

Journal name: International Journal of Health Policy and Management (IJHPM)

Authors' information: Luc L. Hagenaars[1,2]*, Jennifer Falbe[3], Gwyneth M. Manser[4], Laura A. Schmidt[2]

[1]Department of Public and Occupational Health, Amsterdam UMC Location University of Amsterdam, Amsterdam, The Netherlands.

[2]Philip R. Lee Institute for Health Policy Studies, School of Medicine, University of California San Francisco, San Francisco, CA, USA.

[3]Department of Human Ecology, University of California Davis, Davis, CA, USA.

[4]Geography Graduate Group, University of California Davis, Davis, CA, USA.

***Correspondence to:** Luc L. Hagenaars; Email: l.l.hagenaars@amsterdamumc.nl

Citation: Hagenaars LL, Falbe J, Manser GM, Schmidt LA. Pouring rights contracts between soda companies and public universities: an institutional barrier to sugar-sweetened beverage reduction. Int J Health Policy Manag.2025;14:8879. doi:10.34172/ijhpm.8879

Supplementary file 2. Wave 1 Interview Questions for University Managers Who Work in Departments of Student Health, Dining, Athletics, and Contracts at University of California and California State University Campuses

1. What is your position within your department, and how long have you worked there?

2. How familiar are you with the University’s beverage contract?

3. In what ways, if any, does the University’s contract affect your unit?

4. What do you think about the University’s beverage contract?

5. What do you think is the primary benefit beverage companies seek from these contracts?

6. What are the primary benefits the University seeks from a beverage contract?

7. In what ways, if any, does the University’s exclusive beverage contract affect the selection of beverages and advertising?

8. Did you or your department participate in the RFP process, and can you elaborate on the process?

9. In your opinion, how would the campus and your unit be different if there was no exclusive beverage contract?

10. Is there anything else you would like to share with us?

11. Do you have any colleagues or contacts at other universities you recommend we talk to about beverage contracts?
