# Supplementary material for: Pouring Rights Contracts Between Soda Companies and Public Universities: An Institutional Barrier to Sugar-Sweetened Beverage Reduction
Source: Int J Health Policy Manag. 2025 Nov 5;14:8879. doi: 10.34172/ijhpm.8879 (PMC12958230; doi:10.34172/ijhpm.8879)
Supplement: Supplementary file 3 — Wave 2 Interview Questions for University Managers Who Work in Departments of Dining, Athletics, and Contracts and Who Are Involved in Negotiating and/or Implementing Pouring Rights Contracts at University of California and California State University Campuses. [file ijhpm-14-8879-s003.pdf]

Article title: Pouring Rights Contracts Between Soda Companies and Public Universities: An Institutional Barrier to Sugar-Sweetened Beverage Reduction

Journal name: International Journal of Health Policy and Management (IJHPM)

Authors' information: Luc L. Hagenaars[1,2]*, Jennifer Falbe[3], Gwyneth M. Manser[4], Laura A. Schmidt[2]

[1]Department of Public and Occupational Health, Amsterdam UMC Location University of Amsterdam, Amsterdam, The Netherlands.

[2]Philip R. Lee Institute for Health Policy Studies, School of Medicine, University of California San Francisco, San Francisco, CA, USA.

[3]Department of Human Ecology, University of California Davis, Davis, CA, USA.

[4]Geography Graduate Group, University of California Davis, Davis, CA, USA.

***Correspondence to:** Luc L. Hagenaars; Email: l.l.hagenaars@amsterdamumc.nl

Citation: Hagenaars LL, Falbe J, Manser GM, Schmidt LA. Pouring rights contracts between soda companies and public universities: an institutional barrier to sugar-sweetened beverage reduction. Int J Health Policy Manag.2025;14:8879. doi:10.34172/ijhpm.8879

Supplementary file 3. Wave 2 interview Questions for University Managers Who Work in Departments of Dining, Athletics, and Contracts and Who Are Involved in Negotiating and/or Implementing Pouring Rights Contracts at University of California and California State University Campuses

1. Description of campus its pouring right contract
    a. Can you talk a bit about how the pouring right contract at your campus works?

2. Role in campus its pouring right contract
    a. Can you talk a bit about your role in your campus its pouring right contract?

3. Purpose of campus its pouring right contract
    a. What is this contract's function, in your perception?
    b. What is its value, what is good about it?
    c. What does it mean to you and to the university to have a pouring right contract in place?
    d. Has your perspective on the function/meaning of these contracts changed over time? If so, how?

4. Opposition to pouring right contracts. If not discussed by participant yet, shortly describe there exists opposition to pouring right contracts from various angles (e.g., health equity, sustainability).
    a. How do students on your campus look at these issues?
    b. How do you perceive this opposition?
    c. Would you perceive this opposition differently if it were targeting other industries?
    d. Has your perspective on this opposition changed over time? If so, how?

5. Noticing there's a certain kind of tension related to pouring rights and various university goals including the university's and athletics department's finances, health, sustainability, etcetera. What's your vision on dealing with these tensions?

6. Is there anything else that you think I should ask/may be interested in?

7. Is there any person or organization that you advise me to also interview?
